# Profiling of promoter occupancy by the SND1 transcriptional coactivator identifies downstream glycerolipid metabolic genes involved in TNFα response in human hepatoma cells

**DOI:** 10.1093/nar/gkv858

**Published:** 2015-08-31

**Authors:** Enara Arretxe, Sandra Armengol, Sarai Mula, Yolanda Chico, Begoña Ochoa, María José Martínez

**Affiliations:** Department of Physiology, Faculty of Medicine and Dentistry, University of the Basque Country (UPV/EHU), 48940 Leioa, Bizkaia, Spain

## Abstract

The NF-κB-inducible Staphylococcal nuclease and tudor domain-containing 1 gene (*SND1*) encodes a coactivator involved in inflammatory responses and tumorigenesis. While SND1 is known to interact with certain transcription factors and activate client gene expression, no comprehensive mapping of SND1 target genes has been reported. Here, we have approached this question by performing ChIP-chip assays on human hepatoma HepG2 cells and analyzing SND1 binding modulation by proinflammatory TNFα. We show that SND1 binds 645 gene promoters in control cells and 281 additional genes in TNFα-treated cells. Transcription factor binding site analysis of bound probes identified motifs for established partners and for novel transcription factors including HSF, ATF, STAT3, MEIS1/AHOXA9, E2F and p300/CREB. Major target genes were involved in gene expression and RNA metabolism regulation, as well as development and cellular metabolism. We confirmed SND1 binding to 21 previously unrecognized genes, including a set of glycerolipid genes. Knocking-down experiments revealed that SND1 deficiency compromises the glycerolipid gene reprogramming and lipid phenotypic responses to TNFα. Overall, our findings uncover an unexpected large set of potential SND1 target genes and partners and reveal SND1 to be a determinant downstream effector of TNFα that contributes to support glycerophospholipid homeostasis in human hepatocellular carcinoma during inflammation.

## INTRODUCTION

Staphylococcal nuclease and tudor domain-containing 1 (*SND1*) is an evolutionarily conserved gene, usually present as a single copy in all organisms from fission yeast to humans (1–4) that is controlled by transcription factors Sp1 and Nuclear transcription factor Y (NFY) (5,6). The protein product of this gene, SND1 (also called Tudor-SN or p100), is a multidomain protein that appears to have diverse functions in mammalian cells. SND1 contains N-terminally four repeats of staphylococcal nuclease-like domains (SN1–SN4) followed by a tudor domain and a fifth truncated SN domain at the C-terminal end (7,8). This particular structure allows the protein to interact with nucleic acids, individual proteins and protein complexes in a promiscuous manner. For example, the SN3 and SN4 domains generate a compact basic surface implicated in the recognition and cleavage of double-stranded RNA (9), while the Tudor domain contains an aromatic cage that traps methyl groups and may be considered a methylation ‘reader’ (7,10) and the amino-terminal region interacts with proteins containing histone acetyltransferase activity (11). SND1 association with lipid droplets was found in hepatocytes (12) and milk-secreting cells (13) in steatogenic conditions, although the domain responsible for the recognition of partner surface components is not known.

Originally discovered as a transcriptional coactivator of the Epstein-Barr virus nuclear protein 2 (EBNA-2) (14), SND1 was further demonstrated to interact functionally with transcription factors STAT5 (15), STAT6 (11,16), c-Myb (17) and PPARγ (18). These transcription factors regulate relevant biological processes. For instance, STAT5 and STAT6 are critical mediators in the immune and inflammatory responses (19,20), c-Myb is involved in cell growth and differentiation (21) and PPARγ controls adipocyte differentiation in adipose tissue homeostasis (18,22). In most cases SND1 appears to act as a canonical coactivator, favoring increased access of the RNA polymerase II machinery to DNA in response to activating signals. It does this by bridging between the transcription factor and defined components of the basal transcription machinery (11,16,17).

New information emerging over the last decade has demonstrated that SND1 receives regulatory input through multiple stimuli, serving what are considered post-transcriptional regulatory functions to control important physiological events. SND1 is involved in RNA maturation, including but not limited to spliceosome assembly and pre-mRNA splicing (10,23,24), RNA stability (25), RNA editing and interference (9,26–28), and microRNA processing (29), response to environmental (30–32) and metabolic (12,13,33) stressors and lipoprotein lipid secretion (34). Importantly, SND1 has been proposed to serve oncogenic functions. Overexpression of SND1 has been observed in breast (35,36), prostate (27), colon (37) and brain (38) cancer. It also plays a relevant role in promoting hepatocellular carcinoma (HCC) initiation (39) and progression (40,41) and expansion and activity of tumor initiating cells in multiples types of cancers (36). Despite this, the role of SND1 in cancer development and the molecular mechanisms underpinning *SND1* gene promoter activation are far from being elucidated. Therefore, SND1 is an interesting protein and how it can be involved in multiple and seemingly unrelated processes is an important question yet to be answered.

TNFα is a proinflammatory cytokine that plays an essential role in the regulation of inflammation, immune regulation, cell death and cancer. TNFα is particularly important in the liver pathophysiology (42). Liver parenchymal cells are a target of TNFα—and other proinflammatory cytokines—produced by the adipose tissue and non-parenchymal liver cells (mainly activated resident macrophages and endothelial cells). In this way, proinflammatory cytokines in the portal circulation might actually modulate the functionality of hepatocytes and contribute to liver carcinogenesis (43) and other aberrancies associated with adipose tissue expansion (obesity) or insulin resistance (43,44). Recent reports have suggested the existence of a complex crosstalk between SND1 and the TNFα-induced NF-κB signaling in human hepatoblastoma cells. Our own work demonstrated that *SND1* is an inducible gene that responds to TNFα through a transcriptional network involving Sp1, NFY and NF-κB binding on the promoter (6). In response to stimuli such as TNFα, in brief, NF-κB inhibitors proteins are released and the resulting activated NF-κB dimers translocate within the nucleus and activate their target genes (45). In many cancer cells, NF-κB has a constitutively high level of activity which has been suggested to correlate with cancer development and progression (46). Santhekadur *et* *al*., in an elegant study, demonstrated that SND1 promotes angiogenesis in HCC by activating NF-κB, resulting in the induction of the oncomiR-221, angiogenin and CXCL16 (41). Consequently, the interplay between the SND1 and the NF-κB activation status might provide an important link in coordinating the liver response to tumorigenic and inflammatory stimuli.

Identification of the interaction networks required for the precise regulation of SND1 activity and of the partners and targets underpinning SND1's action in transcriptional regulation is fundamental for understanding this protein function. Here, we have performed a genome-wide search for endogenous SND1 binding sites by chromatin immunoprecipitation (ChIP)-chip assays on human hepatoma HepG2 cells in normal and TNFα-induced inflammatory conditions. This study represents the first large-scale attempt to address the nature of gene targets of SND1 at cellular level. We have identified a broad collection of potential SND1 target genes with functions in transcription regulation, development and regulation of cellular metabolism and validated a subset. Subsequent knocking-down experiments have revealed that SND1 is a determinant downstream effector of TNFα, helping to sustain glycerophospholipid homeostasis in human HCC cells.

## MATERIALS AND METHODS

### Cell culture, gene silencing and luciferase reporter assays

HepG2 cells (ATCC) (3.5 × 10^6^) were seeded in 10 cm diameter plates and grown in Eagle's minimal essential medium (EMEM) (ATCC) supplemented with 2 mM L-glutamine, 100 IU/ml penicillin, 100 μg/ml streptomycin (all from Sigma-Aldrich) and 10% (v/v) fetal bovine serum (ATCC) at 37°C and 5% CO_2_. Cultures were either left untreated or treated with TNFα (50 ng/ml) for 24 or 8 h before harvesting for ChIP-chip or gene expression assays, respectively.

For knockdown of endogenous SND1, siRNA oligonucleotide reverse transfection of cells was made in 6-well plates for mRNA and protein determination and in 96-well plates for the reporter assays. Predesigned SND1 siRNA (Life Technologies) at 10 nM final concentration and 5 or 0.25 μl lipofectamine (Life Technologies) were dissolved in Opti-MEM I Reduced Serum (Life Technologies). Then, 2–2.5 × 10^5^ HepG2 cells in 2.5 ml EMEM (6-well plates) or 1.2 × 10^4^ cells in 0.1 ml EMEM (96-well plates) were added to the siRNA-containing well and cultured for 48 h. A negative siRNA (Life Technologies) was used as a control. For mRNA and protein determination, 8 h before harvesting, cells were treated with TNFα (50 ng/ml). For luciferase activity assays, medium was replaced by 0.1 ml fresh EMEM medium with or without TNFα (50 ng/ml), and cells were transfected using 0.6 μl X-tremeGENE HP transfection reagent (Roche Applied Science) and received 0.1 μg of the appropriate CHPT1 (−1100, +1250), LPGAT1 (−1100, +450), LPIN1 (−2959, −2842 joined to −211, +900) or PTDSS1 (−800, +800) promoter cloned into the Firefly luciferase reporter vector pRP (VectorBuilder, Cyagen Biosciences) and 0.1 μg of Renilla luciferase pRL-TK (Promega), as internal control for transfection efficiency. Mock transfections with the empty vector pcDNA3 were carried out in all cases. After 24 h, cells were lysed and luciferase activity measured using the Dual-Luciferase Reporter Assay System (Promega) in a Synergy HT Multi-Detection Microplate Reader (BioTek Instrument Inc) (33). Firefly luciferase activity was normalized to Renilla luciferase activity. Assays were performed in triplicate and luciferase values were expressed as relative activity, setting to 1.0 the value in untreated cells expressing endogenous levels of SND1.

### Chromatin immunoprecipitation

ChIP was performed using the EpiTectChIP One-Day-Kit (SABiosciences) following the manufacturer's instructions. Briefly, exponentially growing 4–6 × 10^6^ HepG2 cells, control and TNFα-treated, were washed and protein–DNA complexes were cross-linked in 1% formaldehyde in phosphate buffered saline (PBS) for 10 min at 37°C. Cells were washed twice with PBS, harvested in protease inhibitor cocktail and pelleted by centrifugation at 800 x *g* for 10 min at 4°C. The cell pellet was resuspended in lysis buffer containing a protease inhibitor cocktail and incubated for 15 min. Cross-linked material was fragmented by sonication to shear chromatin to 800–1000 bp using a Soniprep 150 sonifier (MSE) at high power. The sonicated chromatin solution was precleared by incubating with Protein A beads for 50 min at 4°C, aliquoted and incubated overnight at 4°C with anti-SND1 antibody (34) or non-immune serum IgG (SABiosciences) as a negative control. Afterward, Protein A beads were added and the incubation was continued for 1 h. Precipitated complexes were reverse cross-linked and DNA fragments were purified on the immunoprecipitates along with the input material following the manufacturer's instructions. Purified DNA was used for polymerase chain reaction (PCR) and ChIP-chip analysis. ChIP experiments were run in triplicate.

### ChIP-chip assays and data analyses

Purified ChIP DNA was amplified with the GenomePlex Complete Whole Genome Amplification kit (Sigma-Aldrich) following the manufacturer's instructions. Using the Agilent Genomic DNA Enzymatic Labeling Kit (Agilent Technologies), the input samples were labeled with Cyanine-3 (Cy3) and the immunoprecipitated sample with Cyanine-5 (Cy5) according to Agilent instructions. Labeled nucleotides were hybridized to Agilent SurePrint G3 Human Promoter ChIP-chip Microarray 1 × 1M, Agilent Microarray Desing ID 027811 p/n G4873A. The microarray contains over 960 000 oligonucleotides covering the region −9/+2 kb from the transcription start site (TSS) of 21 000 well-defined genes along the human genome. The hybridation was performed in SureHyb hybridation chambers (Agilent Technologies), incubating 5 μg Cy3 (input sample) and 5 μg Cy5 (IP sample) in 490 μl during 40 h at 65°C and 20 rpm. Arrays were washed using the Stabilization and Drying Solution and the ozone-barrier slide covers in order to minimize the ozone-mediated Cy5 degradation, and finally scanned, all according to the manufacturer instructions. Labeling and hybridization was performed by the genomic and proteomic core facility (SGIKer) of the University of the Basque Country.

The information was extracted with the Feature Extraction Software (version 10.7.3.1), and the SND1-DNA binding events were recognized by the Genomic Workbench Lite Edition program (version 6.5). This program also calculates false discovery rate (FDR) for each peak.

### Data mining

The identification of over-represented motifs in peaks detected by ChIP-chip, and of bound probes and genes corresponding to the probes was done using the Cis-regulatory Element Annotation System (CEAS) server at http://ceas.cbi.pku.edu.cn/ (47). The identification of the enriched binding sites and motif analysis was done by CEAS and MEME (Multiple EM for Motif Elicitation) (http://meme.nbcr.net/meme/intro.html/) (48), and compared with the existing motif matrixes available in Jaspar and Transfac. The Gene Ontology (GO) analyses were performed by DAVID (Database for Annotation, Visualization and Integrated Discovery) at http://david.abcc.ncifcrf.gov/ (49), PinkThing at http://pinkthing.cmbi.ru.nl/l (50) and CEAS. The parameter used in this study was Gene Ontology Biological Process term, level 5. The involvement of SND1 target genes in metabolic and signaling pathways was determined using the KEGG (Kyoto Encyclopedia of Genes and Genomes) database at http://www.genome.jp/kegg/.

### Quantitative real-time PCR and gene expression analyses

The quantification of immunoprecipitate-enriched DNA sequences to validate ChIP-chip assays was performed by quantitative real-time PCR (qPCR) analyses on positive regions of representative genes. The double stranded DNA dye SYBR green (Life Technologies) methodology was used for the amplification reaction, using 5 μl of the immunoprecipitate material and 0.1 μM of the specific primer set in an ABI 7000 Sequence Detection System (Applied Biosystems). Sequences of PCR primers for ChIP-chip validation are listed in Supplementary Table S1. The PCR reactions were as follows: 94°C for 3 min; 40 cycles at 94°C for 20 s, 59°C for 30 s and 72°C for 30 s; and final extension at 72°C for 2 min. PCR reactions were performed in triplicate on all ChIP samples used for ChIP-chip. The results are given as the enrichment of the immunoprecipitation relative to the negative control.

Transcript expression of selected genes was determined by reverse transcription quantitative real-time PCR (RT-qPCR) in control and TNFα-treated HepG2 cells, both expressing endogenous levels of SND1 or residual SND1 levels after SND1 knocking-down by siRNA treatment. Sequences of PCR primers for expression analysis are listed in Supplementary Table S2. RNA extraction (3–5 × 10^6^ HepG2 cells) was performed with TRIzol reagent (Life Technologies) and quantified (ND-1000 spectrometer, NanoDrop Technologies). First strand cDNA was synthesized from 1 μg RNA of each cell sample using the Transcriptor First Strand cDNA Synthesis Kit (Roche Applied Science). The cDNA was then used as the template for individual PCR reactions, programmed as stated above. Normalization was performed using normalization factors computed by GeNorm for 18 s, β-actin, GAPDH and TATA box binding protein mRNAs, as detailed previously (51). The experiment was performed in triplicate.

### Immunoblot analysis

The level of SND1 protein was determined by western blot analysis in the nuclear and cytoplasmic fractions of control and TNFα-treated HepG2 cells expressing endogenous or residual levels of SND1 after SND1 knocking-down by siRNA treatment. Cells were lysed and the nuclear and cytoplasmic fractions were separated using a Nuclear Extraction Kit (Panomics), according to the manufacturer's indications. The levels of SND1, CHPT1 and LPGAT1 protein were also measured in whole cell lysates. Protein concentrations were determined using a commercially available kit (Bio-Rad). Ten to twenty micrograms of protein were loaded in each lane, fractionated on 9% sodium dodecyl sulphate-polyacrylamide gel electrophoresis at 170 V for 1 h and transferred to PVDF membranes (Bio-Rad) by semi-dry transference (1 h at 20 V). SND1, CHPT1 and LPGAT1 were detected by using anti-SND1 (34) (0.3 μg/ml), anti-CHPT1 (0.3 μg/ml, Thermo Scientific) and anti-LPGAT1 (0.3 μg/ml, Sigma-Aldrich) antibodies, respectively. Normalization was performed with β-tubulin (cytoplasm and whole cell lysates) or histone H3 (nucleus), using mouse anti-β-tubulin (Santa Cruz Biotechnology) and anti-H3 (Cell Signaling Technology) primary antibodies. Peroxidase-conjugated horse anti-mouse IgG and goat anti-rabbit IgG (Sigma-Aldrich) were used as secondary antibodies. Detection was performed by ECL (GE Healthcare Life Sciences) and quantification by optical densitometry using QuantityOne software (Bio-Rad). Results are expressed as fold-change relative to the protein level in control cells.

### Quantification of cellular lipids

The major glycerophospholipid classes (phosphatidylcholine (PC), phosphatidylserine (PS), phosphatidylethanolamine (PE) and phosphatidylinositol (PI)), free cholesterol (FC), triacylglycerol (TAG) and cholesteryl esters (CE) were quantified in control and TNFα-treated HepG2 cells expressing endogenous or residual levels of SND1 after SND1 knocking-down by siRNA treatment. Cells were lysed and lipids were extracted from cell lysates with a preparation of CHCl_3_/MeOH (52) and dried. The lipids were separated by thin-layer chromatography and quantified by optical density densitometry as described previously (53) using standards from Avanti Polar Lipids, Inc.

### Statistical analyses

Except for the hybridization binding in microarrays, results are reported as the mean ± SD and were analyzed by the two-tailed Student's *t*-test. Statistical significance is defined as *P* ≤ 0.05 unless otherwise stated.

### Data release

The ChIP-chip data have been submitted to GEO data set NCBI (GSE61539) and will be released upon publication.

## RESULTS

### ChIP-chip analysis identifies a broad set of SND1 binding regions and target genes in human hepatoma HepG2 cells

**Figure 1.**
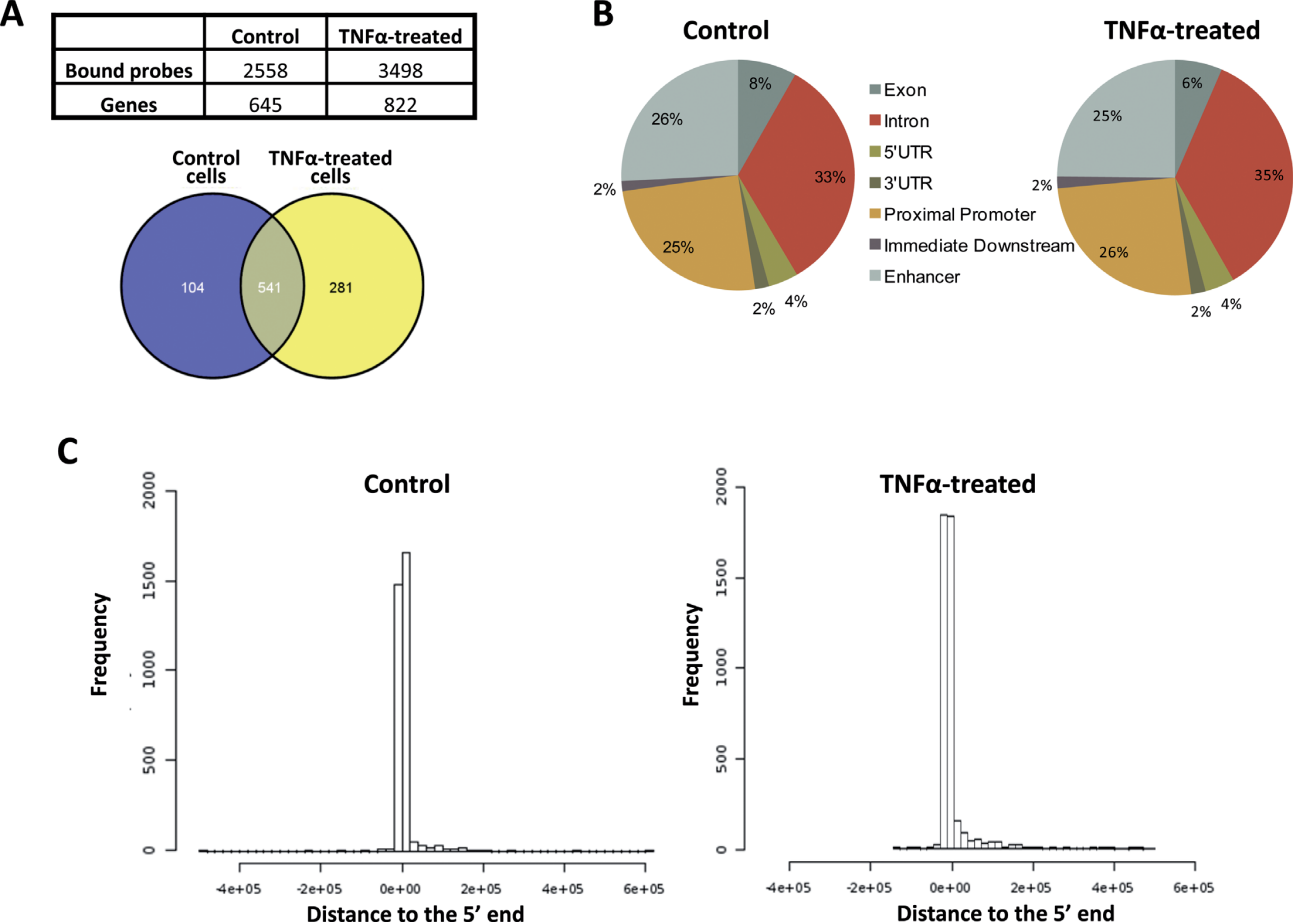
Analysis of SND1 binding regions. HepG2 cells were treated with TNFα (50 ng/ml, 24 h) or left untreated (control) before performing ChIP-chip analysis for SND1 binding. (**A**) Bound probes and Venn diagrams of the corresponding genes. (**B**) Identification of the genomic location of SND1 binding regions using PinkThing. (**C**) Positional distribution of the identified SND1 binding regions relative to the 5′ end in control and TNFα-treated cells. Graphs (B and C) are representative of three experiments with similar results.

In a previous report, we documented that *SND1* transcriptional activity is induced in HepG2 cells by TNFα treatment through NF-κB binding to its response element on the *SND1* promoter (6). Early studies established that SND1 acts as a transcription coactivator in the cellular response to inflammation (11,14–17). To gain insight into the role of endogenous SND1 in transcription regulation, we investigated the whole genome map of SND1 binding sites by ChIP followed by microarray hybridization (ChIP-chip) assays in untreated HepG2 cells and upon TNFα stimulation. Immunoprecipitated chromatin with an anti-SND1 antibody was amplified, fluorescence-labeled and then hybridized into Agilent microarrays that contained oligonucleotide probes covering promoter regions of −9 to +2 kb of the TSS of 21 000 human genes. The whole procedure was repeated three times. Using the Agilent Feature Extraction Software, we selected the SND1 binding sites identified in each of the three biological replicates at the level of significance*P*[Xbar] < 0.05. A total of 645 genes in control samples (Supplementary Table S3) and 822 in TNFα-treated cells were found to be bound to SND1 in the microarrays (Figure 1A). TNFα-treatment promoted SND1 binding to 281 additional genes (Supplementary Table S4) and the release of SND1 from 104 gene promoters (Supplementary Table S5), whereas there was effective binding to 541 genes in the two experimental groups. This implies that SND1 may bind the promoters of 926 gene candidates, depending on the cellular context. Protein–DNA crosslinking was performed by formaldehyde. This crosslinker also has the potential to connect with proteins that are not in direct contact to DNA (54). This means that the genes identified in this ChIP-chip study with anti-SND1 antibodies might be direct targets of SND1 or be bound through other partner proteins.

Using the online PinkThing tool to analyze the genomic distribution of SND1 binding sites, we found they were similarly distributed in the immunoprecipitates from TNFα treated and untreated cells. Roughly half of the binding sites were located upstream within the proximal and distal gene promoters (51–53%) and the others were located downstream mainly in the first exon and intronic regions of the target genes (Figure 1B). Most of these bindings occur close to TSS (Figure 1C). The more distal binding regions are not covered by the array and therefore binding regions were not expected beyond +2 kb of TSS and if found, indicate misclassification of the software. Bearing in mind the limitations of this array, we cannot discard the possibility that SND1 binds other genomic loci outside of the proximal promoters.

STAT5, STAT6, c-Myb and PPARγ are established partners of nuclear SND1. We hypothesized that binding motifs for these transcription factors would be expected to be over-represented in the promoters of the SND1-bound gene set identified by ChIP-chip. To test this hypothesis, we analyzed the SND1-bound probes of all promoters with the CEAS tool, which determines the number of binding motifs for a given transcription factor in a set of DNA sequences and compares the data with the number of motifs for the same factor in the whole genome. CEAS revealed enriched motifs for STAT5, STAT6 and c-Myb in the target promoters (Table 1), but also for novel transcription factors involved in stress response (i.e. HSF, HSF1, HSF2 and ATF), viral infection (i.e. STAT1 and STAT3), cell proliferation (i.e. MEIS1/AHOXA9, E2F, E2F1, STAT3, PAX2/3/8 and SPI1) and metabolic regulation (i.e. p300 and CREB) with *P*-values below 10^−5^ (Table 1). Confirmation of the identified motifs was done by MEME (data not shown). These findings support a role for SND1 as a transcriptional coregulator and extend the identity of transcription factors with which nuclear interaction is feasible.

**Table 1.** Transcription factors with over-represented binding motifs in SND1-bound probes, as determined by CEAS

Transcription factor	Targets (N)*	Fold enrichment	*P*-value
MEIS1/AHOXA9	2	1.72814	0.0
STAT5A	1163	2.28559	3.21E-130
HSF	799	2.94156	2.93E-142
STAT1	924	2.45670	1.53E-119
Pax-2	858	2.48767	1.51E-113
STAT6	952	2.34214	1.01E-112
ZF5	620	2.85921	1.33E-104
Pax-8	785	2.21451	9.50E-81
HSF2	850	2.01900	1.14E-69
Elk-1	403	2.88062	1.24E-67
STAT3	751	2.03191	3.43E-62
MYB	986	1.83014	3.92E-61
c-Myb	970	1.70816	2.17E-47
E2F	381	2.45083	5.72E-47
SPI-1	1040	1.58998	2.87E-38
Ncx	678	1.90196	3.59E-46
HSF1	742	1.81986	2.92E-44
CREB	279	2.69071	2.17E-40
GATA-1	754	1.65783	5.45E-32
GAGA	333	2.07745	3.66E-27
HOXA3	521	1.75211	3.87E-26
E74A	236	2.35228	3.32E-25
p300	745	1.56745	1.30E-24
Pax-3	126	3.17628	2.82E-23
E2F-1	93	3.98362	3.55E-22
c-ETS	712	1.51693	9.49E-20
Msx-1	557	1.54937	2.16E-16
SAP-1	34	4.96056	2.26E-08
Hairy	67	2.82969	9.02E-08
STAT	14	9.48605	6.54E-05
ATF	47	2.94466	7.73E-05

*Number of binding motifs for each transcription factor.

### Ontological analysis of SND1 target genes

Putative SND1 target genes were categorized according to the criteria of GO for biological processes and molecular functions by DAVID. In cells growing in a standard environment, this analysis revealed that the most over-represented biological process terms (level 5) were the regulation of gene expression, the regulation of transcription and DNA and RNA metabolism, cellular biosynthetic processes, organ morphogenesis and central nervous system development, with *P*-values for enrichments between 3.3 × 10^−4^ and 2.2 × 10^−11^ (Table 2). Notably, ‘fat cell differentiation’, ‘lactation’ and ‘response to heat’ are some of the few terms (level > 5) enriched in TNFα-treated but not in untreated HepG2 cells (data not shown). This suggests a potential intervention of SND1 in the cell reprogramming of lipid metabolism to manage stressful conditions.

**Table 2.** Biological process classification of SND1-bound genes in control HepG2 cells, as determined by DAVID

Category	GO term	ID	Genes (N)*	%	*P*-value	Benjamini
GOTERM_BP_5	Regulation of gene expression	GO:0010468	162	2.5	2.20E-11	2.90E-08
GOTERM_BP_5	Regulation of nucleobase, nucleoside, nucleotide and nucleic acid metabolic process	GO:0019219	159	2.5	5.30E-11	3.60E-08
GOTERM_BP_5	Regulation of transcription, DNA dependent	GO:0006355	150	2.3	6.00E-11	2.70E-08
GOTERM_BP_5	Regulation of cellular biosynthetic process	GO:0031326	163	2.5	1.40E-10	4.90E-08
GOTERM_BP_5	Organ morphogenesis	GO:0009887	48	0.8	3.20E-08	7.20E-06
GOTERM_BP_5	Regulation of RNA metabolic process	GO:0051252	104	1.6	2.80E-07	5.50E-05
GOTERM_BP_5	Neurogenesis	GO:0022008	47	0.7	5.40E-07	8.10E-05
GOTERM_BP_5	Central nervous system development	GO:0007417	36	0.6	2.60E-06	3.30E-04

*Number of genes that are included in each GO category. *P*-value and Benjamini corrections are shown.

Looking at the enriched terms for molecular functions, almost all were related to transcription and transcription factor activity (data not shown), which reinforces the view that SND1 plays a regulatory role of gene expression at the transcriptional level.

KEGG database allows the identification of pathways in which a set of target genes can have an impact. Application of KEGG to our lists of SND1 target genes extracted pathways involved in cancer (including the PI3K-AKT, MAPK and Ras signaling pathways, microRNAs and transcriptional misregulation in cancer), viral infection (HTLV-I, hepatitis B and Epstein-Barr), inflammation (chemokine and cytokine-cytokine receptor interaction), as well as metabolic pathways (Table 3). Because additional genes were incorporated to the same categories in the treated group, the landscape points to the idea that SND1 might participate in the above-mentioned processes both under control conditions and in response to TNFα-promoted inflammatory contexts.

**Table 3.** Classification of SND1-bound genes in control and TNFα-treated HepG2 cells, as determined by KEGG

CONTROL				TNFα-TREATED			
ID	KEGG pathway	Genes (N)*	Gene symbol	ID	KEGG pathway	Genes (N)*	Gene symbol
ko05200	Pathways in cancer	15	*BCL2, BRCA2, CDKN1B, JUP, KITLG, KRAS, LAMB1, MITF, MYC, NFKB2, PDGFRB, RET, RUNX1, TCF7L2, XIAP*	ko05200	Pathways in cancer	19	*BCL2, BRCA2, CCND1, CDK6, CDKN1B, JUP, KITLG, KRAS, LAMB1, MITF, MYC, NFKB2, PDGFRB, PTEN, RET, RUNX1, TGFBR1, TPR, XIAP*
ko04151	PI3K-Akt signaling pathway	12	*BCL2, CDKN1B, DDIT4, EIF4EBP1, GNB2, GNG12, KITLG, KRAS, LAMB1, MYC, PDGFRB, RELN*	ko04151	PI3K-Akt signaling pathway	18	*BCL2, BCL2L11, CCND1, CDK6, CDKN1B, DDIT4, EIF4B, GNB2, GNG12, ITGA1, KITLG, KRAS, LAMB1, LPAR4, MYC, PDGFRB, PTEN, RELN*
ko05166	HTLV-I infection	11	*ADCY8, BUB3, CREM, KRAS, MYC, NFATC2, NFKB2, PDGFRB, POLE3, VDAC3, XIAP*	ko05166	HTLV-I infection	16	*ADCY8, ATF1, BUB3, CCND1, CREM, ELK4, KRAS, MYC, NFATC1, NFATC2, NFKB2, PDGFRB, POLE3, TGFBR1, VDAC3, XIAP*
ko05206	MicroRNAs in cancer	10	*BCL2, BMF, CCNG1, CDKN1B, DDIT4, KRAS, MYC, PDGFRB, SPYR2, TRIM71*	ko05206	MicroRNAs in cancer	15	*BCL2, BCL2L11, BMF, CCND1, CCNG1, CDK6, CDKN1B, DDIT4, KRAS, MIR223, MYC, PDGFRB, PTEN, SPRY2, TRIM71*
ko01100	Metabolic pathways	9	*AGXT2L2, CHPT1, HAAO, HSD17B2, LPGAT1, MGLL, NMT, POLE3, PTDSS1*	ko04080	Neuroactive ligand-receptor interaction	14	*ADRA2A, CHRNB1, CRHR2, GRIA2, GRIA3, GRID2, GRPR, LPAR4, MC5R, NPFFR2, NPY1R, NPY5R, PARD3, TACR2*
ko04014	Ras signaling pathway	9	*FOXO4, GNB2, GNG12, KITLg, KRAS, PAK3, PAK6, PDGFRB, RAB5B*	ko01100	Metabolic pathways	13	*ACAA2, AGXT2L2, CHPT1, HSD17B2, LPGAT1, LPIN1, MGLL, NNT, NTPCR, PCCA, PIGY, POLE3, PTDSS1*
ko05202	Transcriptional misregulation in cancer	9	*CDKN1B, FLT1, JUP, MYC, NR4A3, RUNX1, SIX1, TLX1, TMPRSS2*	ko05202	Transcriptional misregulation in cancer	13	*ATF1, CDKN1B, ELK4, EYA1, FLI1, GRIA3, ID2, JUP, MYC, NR4A3, RUNX1, SIX1, TLX1*
ko04080	Neuroactive ligand-receptor interaction	8	*ADRA2A, CHRNB1, CRHR2, GRIA2, GRID2, MC5R, NPY1R, PARD3*	ko05161	Hepatitis B	12	*BCL2, CCND1, CDK6, CDKN1B, HSPG2, KRAS, MYC, NFATC1, NFATC2, PTEN, TGFBR1, VDAC3*
ko04022	cGMP-PKG signaling pathway	7	*ADCY8, ADRA2A, CACNA1D, GATA4, IRS4, NFATC2, VDAC3*	ko04510	Focal adhesion	12	*BCL2, CCND1, FLNA, ITGA1, LAMB1, PAK3, PAK6, PDGFRB, PTEN, RELN, SHC2, XIAP*
ko04062	Chemokine signaling pathway	7	*ADCY8, CXCR3, CXCR4, GNB2, GNG12, KRAS, PARD3*	ko04014	Ras signaling pathway	10	*FOXO4, GNB2, GNG12, KITLG, KRAS, PAK3, PAK6, PDGFRB, RAB5B, SHC2*
ko05161	Hepatitis B	7	*BCL2, CDKN1B, HSPG2, KRAS, MYC, NFATC2, VDAC3*	ko04010	MAPK signaling pathway	10	*DUSP3, ELK4, FLNA, GNG12, KRAS, MYC, NFATC1, NFKB2, PDGFRB, TGFBR1*
ko04010	MAPK signaling pathway	7	*CACNA1D, DUSP3, GNG12, KRAS, MYC, NFKB2, PDGFRB*	ko04060	Cytokine-cytokine receptor interaction	9	*CXCL12, CXCR4, EDA, EDA2R, IL13RA1, KITLG, PDGFRB, TGFBR1, TSLP*
ko04510	Focal adhesion	7	*BCL2, LAMB1, PAK3, PAK6, PDGFRB, REL, XIAP*	ko04068	FoxO signaling pathway	9	*BCL2L11, BNIP3, CCND1, CDKN1B, FOXO4, IRS4, KRAS, PTEN, TGFBR1*
				ko05203	Viral carcinogenesis	8	*CCND1, CDK6, CDKN1B, KRAS, NFKB2, UBE3A, UBR4, VDAC3*
				ko05220	Chronic myeloid leukemia	8	*CCND1, CDK6, CDKN1B, KRAS, MYC, RUNX1, SHC2, TGFBR1*
				ko04062	Chemokine signaling pathway	8	*ADCY8, CXCL12, CXCR4, GNB2, GNG12, KRAS, PARD3, SHC2*
				ko04144	Endocytosis	8	*CXCR4, NEDD4L, PARD3, PDCD6IP, RAB5B, RET, SH3KBP1, TGFBR1*
				ko05205	Proteoglycans in cancer	8	*CCND1, EIF4B, FLNA, GPC3, HSPG2, IGF2, KRAS, MYC*
				ko04380	Osteoclast differentiation	7	*FOSL2, JUNB, MITF, NFATC1, NFATC2, NFKB2, TGFBR1*
				ko05222	Small cell lung cancer	7	*BCL2, CCND1, CDK6, LAMB1, MYC, PTEN, XIAP*
				ko04360	Axon guidance	7	*CXCR4, KRAS, NFATC2, PAK3, PAK6, ROBO1, SLIT2*
				ko04810	Regulation of actin cytoskeleton	7	*DIAPH2, GNG12, ITGA1, KRAS, PAK3, PAK6, PDGFRB*

*Number of genes that are included in each KEGG category. Only pathways containing seven or more putative target genes are shown.

### Validation of ChIP-chip analysis

The ChIP-chip findings were validated by ChIP followed by quantitative PCR (ChIP-qPCR) in a collection of 41 representative genes of the enriched GO categories using primers complementary to positive binding regions. We used chromatin that has been immunoprecipitated with the anti-SND1 antibody or with the unspecific anti-IgG antibody from untreated and TNFα-treated cells and considered significant enrichment the specific/unspecific amplification ratios >2 (Table 4). ChIP-chip data were confirmed in 37 out of 82 (45.12%) cases. Significant differences in SND1 enrichment of chromatin were corroborated in HepG2 cells versus SND1-silenced cells (Supplementary Table S6). SND1 bound the promoters of 21 genes: *BRCA2*, *CCN1*, *CCND1*, *CD36*, *CDKN1B*, *CHPT1*, *CREM*, *FLNA*, *GK*, *HCFC1*, *HSD17B2*, *IRAK4*, *LPGAT1*, *LPIN1*, *MADD*, *PPARGC1A*, *PTDSS1*, *SCAP*, *TAF10*, *TRAF7* and *WNT7B*. Moreover, enrichment data in control and treated cells in ChIP-qPCR were coincident with those of ChIP-chip in 13 cases.

**Table 4.** Validation of SND1 binding to representative genes in control and TNFα-treated HepG2 cells

Name/Gene ID	Description	ChIP-chip		ChIP-qPCR		FDR
		Control	TNFα	Control	TNFα	
*ACAA2*	acetyl-CoA acyltransferase 2	No	Yes	nd	nd	0.0461
*ADAT1*	adenosine deaminase, tRNA-specific 1	Yes	No	No	No	0.0317
*ATF1*	activating transcription factor 1	No	Yes	nd	nd	0.0708
*AZI2*	5-azacytidine induced 2	No	Yes	nd	nd	0.0683
BRCA2**	breast cancer 2, early onset	Yes	Yes	5.51	3.84	0.0408
*CALM1*	calmodulin 1 (phosphorylase kinase, delta)	No	Yes	nd	nd	0.0446
CCNI**	cyclin I	No	Yes	No	2.05	0.0367
CCND1*	cyclin D1	No	Yes	2.71	2.97	0.0793
CD36*	CD36 molecule (thrombospondin receptor)	Yes	Yes	No	2.06	0.0302
CDKN1B**	cyclin-dependent kinase inhibitor 1B (p27, Kip1)	Yes	Yes	3.13	6.35	0.0432
*CHDH*	choline dehydrogenase	Yes	Yes	No	No	0.0483
CHPT1**	choline phosphotransferase 1	Yes	Yes	7.73	2.05	0.0461
CREM**	cAMP responsive element modulator	Yes	Yes	2.54	3.69	0.0456
*EIF4B*	eukaryotic translation initiation factor 4B	No	Yes	No	No	0.0335
*FADS2*	fatty acid desaturase 2	Yes	Yes	No	No	0.0341
FLNA**	filamin A, alpha	No	Yes	No	4.8	0.0318
GK*	glycerol kinase	Yes	Yes	3.97	No	0.1309
HCFC1*	host cell factor C1 (VP16-accessory protein)	Yes	Yes	No	2.73	0.0400
*HOXA3*	homeobox A3	Yes	Yes	No	No	0.0451
*HOXB9*	homeobox B9	Yes	Yes	No	No	0.0496
*HOXC9*	homeobox C9	Yes	Yes	No	No	0.0364
HSD17B2**	hydroxysteroid (17-beta) dehydrogenase 2	Yes	Yes	2.87	2.97	0.0305
IRAK4*	interleukin-1 receptor-associated kinase 4	Yes	Yes	12.55	No	0.3673
LPGAT1**	lysophosphatidylglycerol acyltransferase 1	Yes	Yes	4.86	12.74	0.0303
LPIN1**	Lipin 1	No	Yes	No	5.12	0.0525
*LRPAP1*	low density lipoprotein receptor-related protein associated protein 1	Yes	Yes	No	nd	0.0301
MADD**	MAP-kinase activating death domain	No	Yes	No	5.03	0.0444
*MBTPS*	membrane-bound transcription factor peptidase, site 2	No	Yes	No	No	0.0769
*MGLL*	monoglyceride lipase	Yes	Yes	No	No	0.0318
*NFKB2*	nuclear factor of kappa light polypeptide gene enhancer in B-cells 2 (p49/p100)	Yes	Yes	No	No	0.0322
*PPA2*	pyrophosphatase (inorganic) 2	Yes	Yes	nd	nd	0.0314
PPARGC1A**	peroxisome proliferator-activated receptor gamma, coactivator 1 α	No	Yes	No	31.89	0.0988
PTDSS1**	phosphatidylserine synthase 1	Yes	Yes	4.2	50.45	0.0311
*PTEN*	phosphatase and tensin homolog	No	Yes	nd	nd	0.0417
*RXRA*	retinoid X receptor, α	Yes	Yes	nd	nd	0.0335
SCAP*	SREBF chaperone	Yes	Yes	2.1	nd	0.0381
*SETD1A*	SET domain containing 1A	Yes	Yes	No	No	0.0673
TAF10**	TAF10 RNA polymerase II, TATA box binding protein (TBP)-associated factor, 30 kDa	Yes	No	2.05	No	0.0300
*TDRD3*	tudor domain containing 3	Yes	Yes	nd	nd	0.0303
TRAF7*	TNF receptor-associated factor 7	Yes	Yes	4.33	No	0.0317
WNT7B*	wingless-type MMTV integration site family, member 7B	Yes	Yes	No	4.92	0.0416

*Indicates that the positive or the negative SND1 binding found in ChIP-chip assays was confirmed by ChIP-qPCR in one condition (21 genes) or **in the two conditions (13 genes). Significant enrichment was considered a specific/unspecific amplification ratio >2. Amplification was not detected (nd) in eight genes. FDR, Benjamini–Hochberg false discovery rate.

### TNFα regulates the expression of a set of SND1 target genes

**Figure 2.**
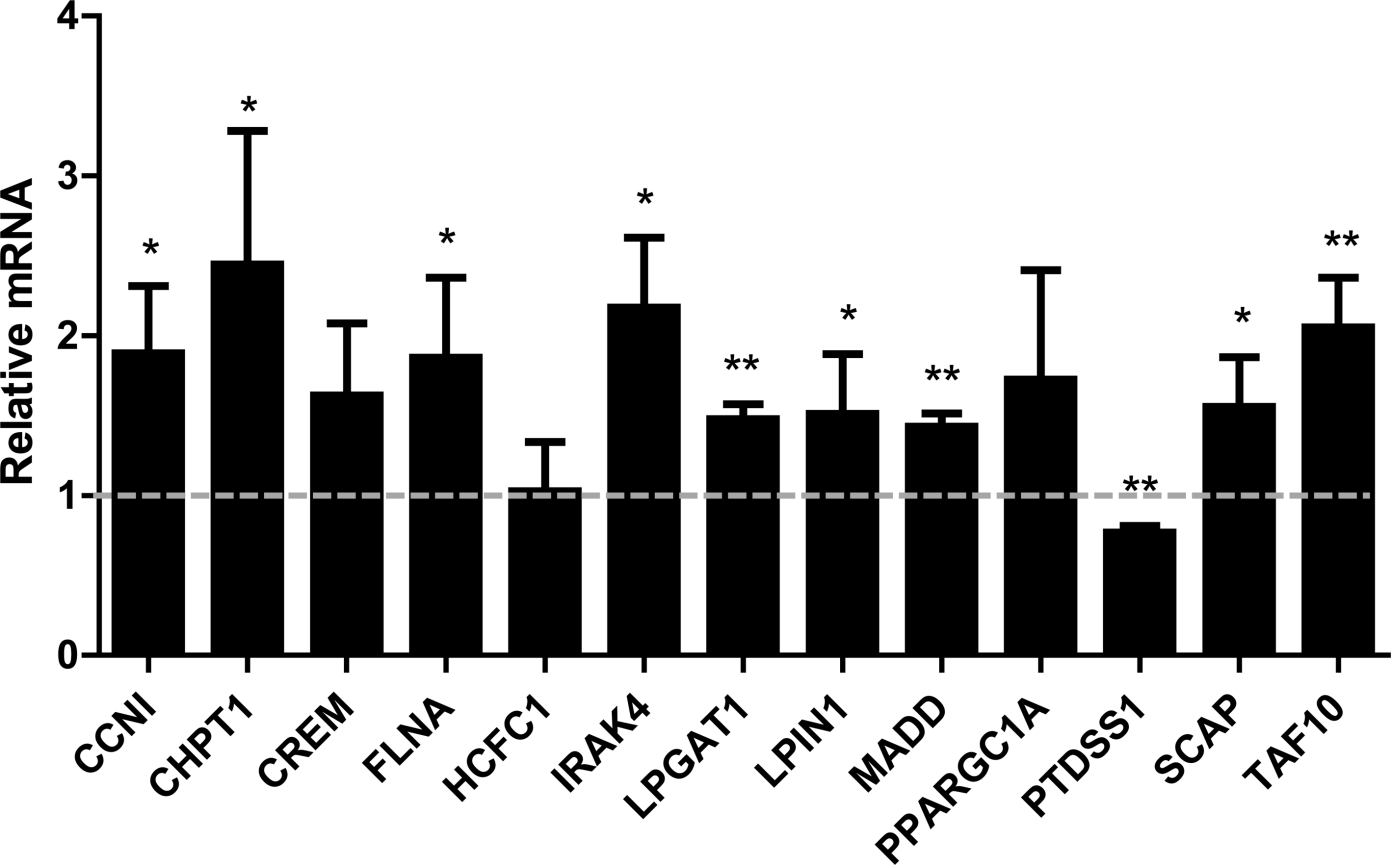
Effect of TNFα treatment on the transcript expression of selected SND1 target genes in HepG2 cells. Transcript expression was determined by reverse transcription quantitative real time PCR in control and TNFα-treated (50 ng/ml, 8 h) HepG2 cells. Results are reported as means ± SD of 3–4 experiments and are expressed relative to the level in control cells, which is shown as a gray grid line. *P ≤ 0.05, **P ≤ 0.01 versus control cells.

We reported in a previous work that extracellular TNFα increases *SND1* transcriptional activity and transcript expression in HepG2 cells (6). Here we hypothesized that if SND1 binding is followed by transcription activation of target genes, TNFα treatment might have an impact on their expression and that, whenever it is mediated by SND1, such an effect should be abolished if SND1 is depleted. To test this, we firstly measured the mRNA levels of a selection of validated SND1 target genes. As seen in Figure 2, the transcript expression of *CCNI*, *CHPT1*, *FLNA*, *IRAK4*, *LPGAT1*, *LPIN1*, *MADD*, *SCAP* and *TAF10* increased upon treatment, whereas that of *CREM*, *HCFC1* and *PPARGC1A* did not change and *PTDSS1* mRNA levels decreased due to TNFα stimulation of HepG2 cells as compared with levels in untreated cells. These findings indicate that SND1 might be implicated in the adaptive response of HepG2 cells to inflammatory stress.

Notably, a subset of four SND1 target genes is involved in glycerophospholipid metabolism. This set includes *CHPT1*, *LPIN1*, *LPGAT1* and *PTDSS1* (the metabolic roles of the gene products are highlighted in blue in Supplementary Figure S1). The *CHPT1* gene product catalyzes the final step of the *de* *novo* synthesis of phosphatidylcholine, *LPIN1* encodes a protein that acts both as a phosphatidate phosphohydrolase involved in diacylglycerol formation from phosphatidic acid and as a coactivator of PPARGC1A, PTDSS1 is involved in the base-exchange mediated conversion of phosphatidylcholine to phosphatidylserine and LPGAT1 catalyzes the reacylation of lysophosphatidylglycerol. SND1 binds the promoters of the four genes in treated and control cells, with the exception of *LPIN1*, which is bound solely during inflammation (Table 4). Overexpression of *CHPT1*, *LPIN1* and *LPGAT1* together with *PTDSS1* repression (Figure 2) is compatible with a change in glycerophospholipid content as part of the cellular response to TNFα. We thus sought to determine whether this was the case and if so, if it was mediated by SND1.

### SND1 is necessary for a normal response of hepatoma cells to TNFα

**Figure 3.**
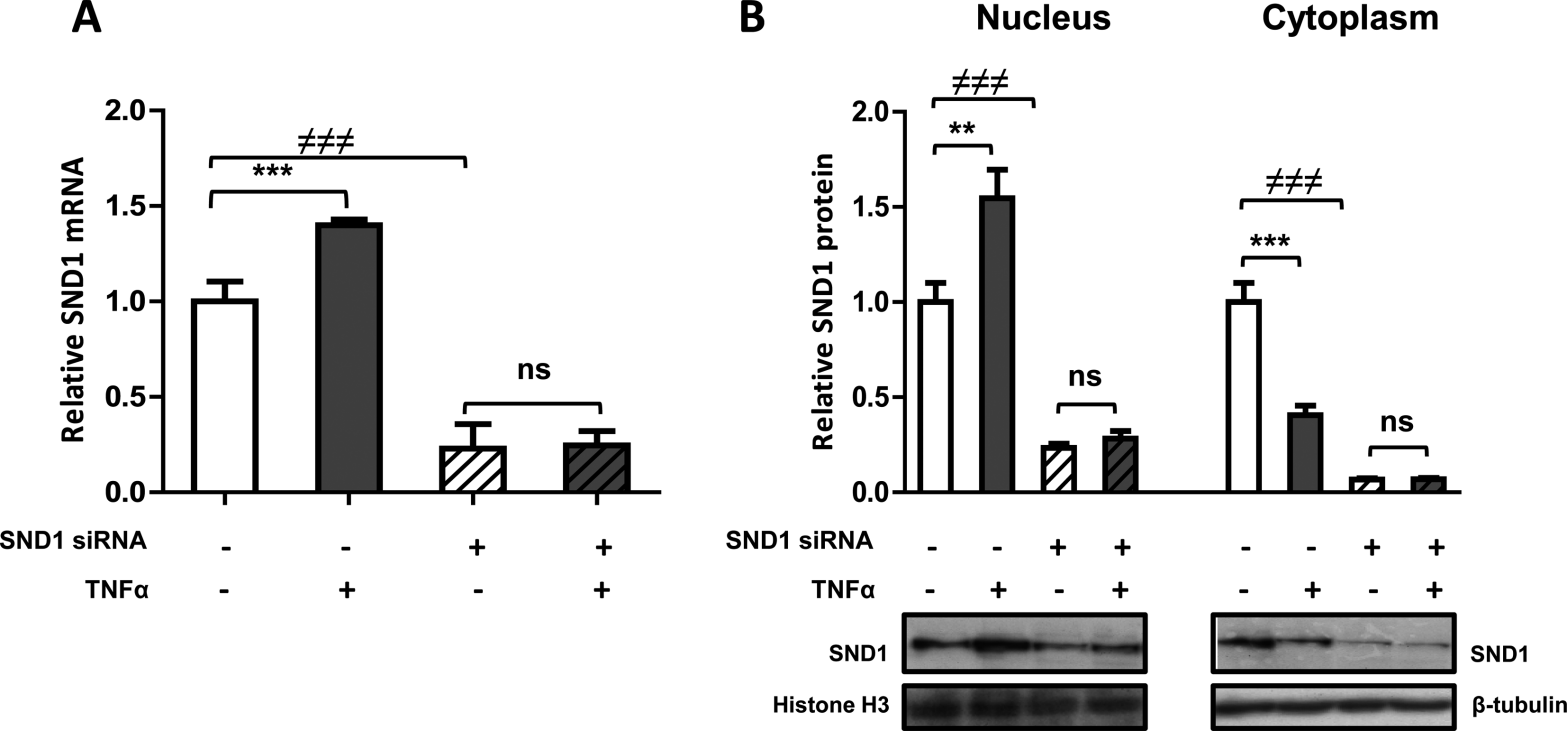
The TNFα-promoted increase in the SND1 mRNA level and the nuclear/cytoplasmic SND1 protein partitioning is not visible in SND1-silenced HepG2 cells. (**A**) The SND1 transcript level and (**B**) the SND1 protein content in nuclei and the cytoplasmic fraction were quantified in control (white bars) and TNFα-treated (50 ng/ml, 8 h) (dark bars) HepG2 cells, expressing basal (solid bars) or residual levels of SND1 after silencing endogenous SND1 (hatched bars). Aliquots of cells (3–5 × 10^6^ cells) were subjected to RNA isolation and individual PCR reactions. Other aliquots (3–5 × 10^6^ cells) were processed for the isolation of nucleus and cytoplasm and subjected to immunoblot analysis for SND1 and normalized with histone H3 and β-tubulin. Results are reported as means ± SD of three independent experiments and expressed relative to untreated cells expressing basal levels of SND1. **P ≤ 0.01, ***P ≤ 0.001 versus control cells; ≠≠≠*P* ≤ 0.001 versus cells expressing endogenous levels of SND1.

To evaluate whether SND1 activity has a role in helping to modulate lipid balance in HepG2 cells, we prepared HepG2 cells with siRNA-silenced SND1 and examined the response of HepG2-KO cells to TNFα, quantifying the intracellular concentration of cholesterol, TAG and the major glycerophospholipids in untreated and TNFα-treated HepG2-KO cells as well as the mRNA levels of expression of the above mentioned target genes involved in lipid metabolism. As shown in Supplementary Figure S2, HepG2-KO cells proliferated at a rate similar to that of cells treated with unspecific siRNA or cells expressing endogenous levels of SND1 in a period of 72 h. The results in Figure 3 show that the SND1-specific siRNA decreased the cellular level of SND1 mRNA by around 80% (Figure 3A) and the protein content both in the nucleus (80%) and cytoplasm (90%) (Figure 3B), as compared with those of a negative control. In earlier studies, we observed that TNFα increases the *SND1* promoter transcriptional activity and the cellular levels of SND1 mRNA in HepG2 cells (6) while protein expression levels were unaffected. Here, we examined if TNFα induced redistribution of the protein between the nuclear and the cytoplasmic compartments. We found that the nuclear protein level increased by ∼50% whereas SND1 protein decreased in the cytoplasm (Figure 3B), suggesting that TNFα promoted nuclear translocation of the SND1 transcriptional coactivator. However, TNFα had no noticeable effects on HepG2-KO cells exhibiting residual levels of SND1 mRNA (Figure 3A) and protein (Figure 3B).

**Figure 4.**
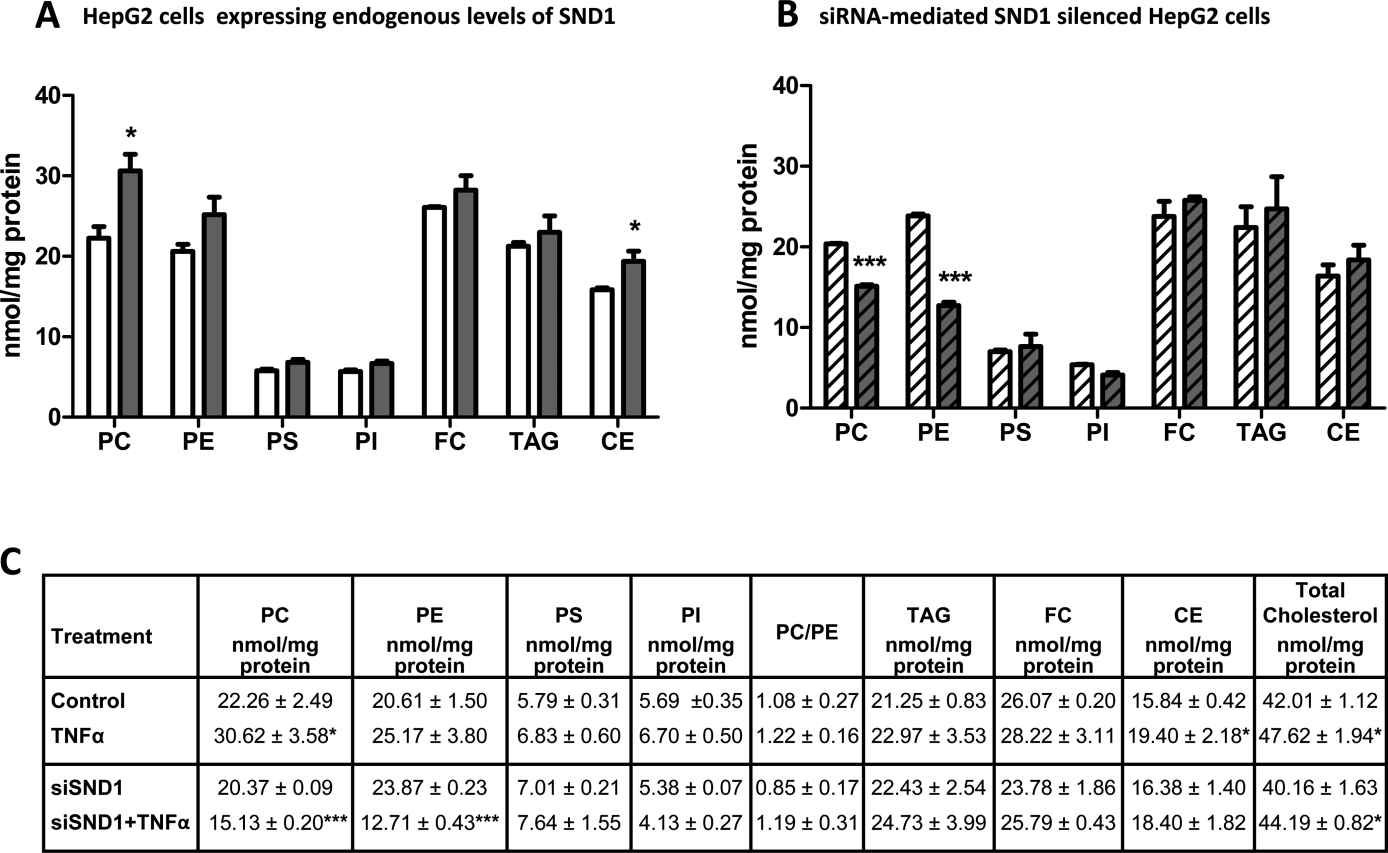
SND1 silencing impedes the adaptation of cellular lipid levels to TNFα stimulation. Phosphatidylcholine (PC), phosphatidylethanolamine (PE), phosphatidylserine (PS), phosphatidylinositol (PI), free cholesterol (FC), triacylglycerol (TAG) and cholesteryl esters (CE) were quantified in control (white bars) and TNFα-treated (50 ng/ml, 8 h) (dark bars) HepG2 cells, expressing either basal (solid bars) or residual levels of SND1 after silencing endogenous SND1 (hatched bars). Lipids were extracted from HepG2 cell lysates, separated by thin-layer chromatography and quantified by optical densitometry as described in ‘Materials and Methods’ section. Results are reported as means ± SD of four independent experiments and expressed as nmol/mg protein. *P ≤ 0.05, ***P ≤ 0.001 versus control cells.

As shown in Figure 4A, TNFα treatment of HepG2 cells increased considerably the cellular content of PC (37.5%), cholesteryl esters (CE) (22.5%) and total cholesterol (13.3%), in line with previous reports (55). The levels of other glycerophospholipid classes—PE, PS and PI—, and of unesterified cholesterol and TAG, tended to increase but rises were not statistically significant. Knockdown of SND1 impeded these changes and unexpected reductions in the cellular PC (22.7%) and PE (46.7%) levels were observed (Figure 4B). Neither TNFα-treatment nor SND1-silencing affected significantly the PC/PE ratio (Figure 4C), a marker of cell proliferation and disease, which is essentially identical as it is in untreated control cells.

**Figure 5.**
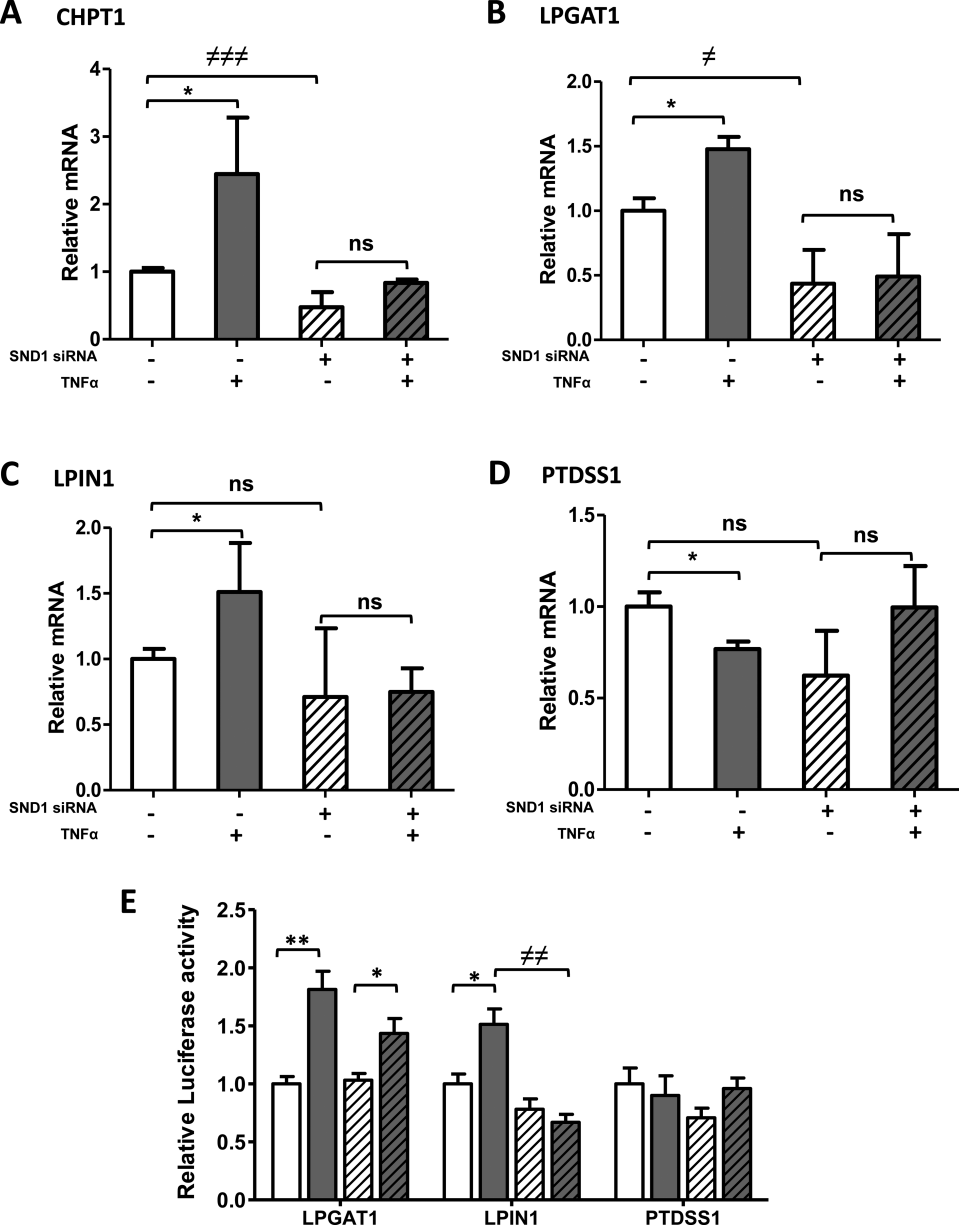
SND1-mediated modulation of the TNFα effect on transcript levels of glycerophospholipid metabolism genes. The transcript levels of CHPT1 (**A**), LPGAT1 (**B**), LPIN1 (**C**) and PTDSS1 (**D**) and the transcriptional activity of the gene promoters (**E**) were determined in control (white bars) and TNFα-treated (50 ng/ml, 8 h for A–D and 24 h for E) (dark bars) HepG2 cells, expressing either basal level of SND1 (solid bars) or after silencing endogenous SND1 (hatched bars). Results are reported as means ± SD of four independent experiments and expressed relative to the values in untreated cells expressing basal levels of SND1. *P ≤ 0.05, **P ≤ 0.01 versus control cells; ≠*P* ≤ 0.05, ≠≠*P* ≤ 0.01, ≠≠≠*P* ≤ 0.001 versus SND1 non silenced cells.

The analysis of the transcript levels of the selected SND1 target genes indicates that both the basal and the TNFα-stimulated mRNA expression of *CHPT1* and *LPGAT1* decreased on SND1 deficiency, suggesting that SND1 binding (Table 4) and activity is necessary for these genes to be expressed in the conditions tested (Figure 5, A and B). The changes in the transcripts did not directly correlate with changes in the amount of CHPT1 and LPGAT1 proteins that maintained unaltered their basal levels (Supplementary Figure S3). Notably, SND1 depletion did not affect the basal mRNA expression of *LPIN1* and *PTDSS1* (Figure 5C and D), with the first gene being unbound and the second one bound to SND1. Despite the fact that strong SND1 binding to these promoters is observed upon TNFα treatment, none of the TNFα-induced changes in transcript expression were observed in SND1-KO cells (Figure 5C and D). The lack of TNFα effect on the gene expression on SND1 deficiency is extensible to the rest of SND1 target genes as shown in Supplementary Figure S4. The data collectively indicate that in HepG2 cells, SND1 mediates the TNFα-induced effect on *CHPT1*, *LPGAT1*, *LPIN1* and *PTDSS1* transcription, contributing also to *CHPT1* and *LPGAT1* transcript expression in non-inflammatory conditions. To test the role of SND1 as transcriptional regulator, we examined the transcriptional activity of the *LPGAT1, LPIN1* and *PTDSS1* promoter vectors carrying the binding regions of SND1 detected by the ChIP-chip analysis (Supplementary Figure S5). We found that cells with endogenous expression of SND1 displayed the expected TNFα-induced stimulation in the LPGAT1 and LPIN1 promoter activity, whereas the activity of PTDSS1 promoter showed no significant modification (Figure 5E). Interestingly, on silencing SND1 expression, the basal luciferase activity of the promoters remained unchanged and the TNFα-induced stimulation of luciferase activity driven by LPIN1 promoter was reverted but not that driven by LPGAT1 promoter. These findings demonstrate that SND1 controls the LPIN1 gene transcription in response to TNFα signaling and suggest that SND1 silencing might be affecting other non-transcriptional roles of SND1 that impact on the expression of the target genes under inflammatory conditions.

## DISCUSSION

Accumulating evidence supports a role for SND1 in the regulation of essential cellular processes such as gene expression (8,15,25–28) and RNA and microRNA processing (9,23,29,32,37), acting at the transcriptional and the post-transcriptional regulatory levels as both a nuclease and a ligand. A number of studies have reported on the ability of SND1 to interact with a range of transcription factors downstream inflammatory signaling and activate transcription of inflammatory genes (11,14,16,56). Here, we report the first attempt to reveal the identity of gene targets of SND1 at whole-genome level by using ChIP-chip analysis, showing that SND1 binds a vast array of genes with functions in transcription regulation, development and regulation of cellular metabolism. The inflammatory environment delineates SND1 binding to a bigger number of genes and highlights the SND1 link with the adaptation of the lipid program to upstream TNFα signaling (Figure 1, Tables 2 and 3, and Supplementary Tables S3–S5). It is appreciative that around half (45%) of the binding sites selected from the ChIP-chip dataset were confirmed by ChIP-qPCR. ChIP-chip microarrays from commercial platforms have been reported to function significantly better at high enrichment values over six-fold, and at low (three to four-fold change) and very low (less than two-fold change) enrichment values, ∼50% of missed targets are detected (57). These findings support the moderate percentage of validation of SND1 binding to the genes as we are characterizing levels of enrichment around two to four-fold change in most of our ChIP samples.

Our findings give a global vision of the versatility of SND1 as an interacting protein with transcription factors and co-regulators. CEAS and MEME bioinformatic tools served to corroborate the over-representation of STAT6, STAT5 and c-Myb motives in the SND1 bound DNA fragments. These transcription factors are well established SND1 partners for activating gene expression in response to cytokines (11,16,17,21,56), prolactin (15) and a mechanical signal (58). We have identified in bound fragments enriched motives for other transcription factors not earlier reported as SND1 partners, including HSF, ATF, STAT1, STAT3, MEIS1/AHOXA9, SpI-1, CREB, p300, E2F1 (Table 1). Notably, most of these transcription factors and co-regulators are implicated in stress-activated mechanisms of protection or associated with oncogenic transformation, viral infection and metabolic regulation (59–61). For instance, p300 is a coactivator that has intrinsic histone acetyltransferase activity and interacts with the SND1-partner EBNA-2 for the LMP1 gene promoter activation (61). During the preparation of the manuscript, the first evidence for a role of SND1 in STAT3 activation and glioma progression has been reported (38). All these results further strengthen the role of SND1 as a transcriptional modulator essential for normal cell growth, differentiation and proliferation of cancer cells, and response to various types of cellular and environmental stresses. Protein–protein interactions are common in recruiting the transcriptional machinery toward selective genes. SND1 transcriptional activity and specificity is likely conferred by interactions with distinctive proteins on the promoters of alternate sets of target genes depending on cellular conditions. The particular multidomain structure of the SND1 protein favors interaction with a variety of nuclear and non-nuclear proteins and multiprotein complexes, explaining but not limited to the transcriptional regulatory function of SND1 but also the post-transcriptional functional aspects of SND1 (7–11,18,32,62).

TNFα is a pleotropic proinflammatory cytokine critical for liver function. Directly—by binding to membrane receptors TNFR1/2—or indirectly—by promoting the release of cytokines—, TNFα can induce a complex network of signaling pathways resulting in either cell damage or cell protection against the cytotoxic inflammatory reaction (63). The key transcription factor in the decision between the hepatocyte life or death is NF-κB, which upregulates the transcription of protective genes (64). One of these genes is SND1 (6), and whether it also has a protective role in the liver is an attractive possibility to be further explored.

Tight regulation of lipid amounts is crucial for cell and organism homeostasis. In fact, lipid and lipoprotein metabolism plays an important role in the immune response to infection or inflammation (55,65–67). TNFα is known to affect the mitochondrial cholesterol levels (68) and the transcriptomic profile of HepG2 cells, altering mainly the expression of genes involved in steroid metabolism and immune defense (69). We analyze the cellular composition of glycerophospholipid, TAG and cholesterol because lipids are necessary structural components and key mediators in proliferation and inflammation and their composition and localization must be finely tuned for the proper functioning of a challenged cell. A remarkable finding is that the SND1 expression level affects to the cellular phospholipid homeostasis but not that of cholesterol.

The connection of SND1 and lipid pathways was firstly evidenced in the prolactin-stimulated lactating mammary gland for the milk lipid bodies formation (1,13). In recent years, our studies with primary hepatocytes have provided significant insights into the association of SNDp102, the rat homolog of human SND1, with large lipid bodies under oleate-induced steatogenic conditions (12) and the existence of a direct relationship between the expression level of SNDp102 and the amount of phospholipids secreted in *d* < 1.015 g/ml and 1.015 < *d* < 1.24 g/ml apoB-containing lipoproteins. Oversecretion affected all phospholipid classes and did not disturb cellular phospholipid homeostasis (34). A reasonable conclusion from these studies would be that the SND1 family of proteins may have a role in the partitioning of phospholipids between lipid bodies and lipoproteins or affects certain steps in the formation of the involved organelles’ phospholipidic surface.

All major homeostatic processes are subject to regulation by a plethora of transcriptional, post-transcriptional and post-translational regulatory events. Phospholipid metabolism is a paradigm of process with multiple layers of regulation. In the mammalian liver, PC can be formed from CDP-choline and diacylglycerol via the Kennedy pathway, from PE via the PE methylation reaction, and from the remodeling pathway driven by an array of phospholipases and acyltransferases (Supplementary Figure S1). Analogous reactions are responsible for PE synthesis. In addition, PC and PE are converted in PS via PTDSS1 and PTDSS2 catalyzed base-exchange reactions (70). An important caveat to bear in mind regarding the hepatic PC homeostasis is that it is also relevant the uptake of PC carried in lipoproteins—mainly in HDL—and the PC output as a bile component and VLDL particles (70). Inflammation and TNFα may impact many of these processes, as part of the physiological events culminating in infection or inflammation resolution. Our findings reveal that four genes encoding enzymatic components of this network—*CHPT1*, *PTDSS1*, *LPGAT1* and *LPIN1*—are under SND1 modulation and suggest that a threshold level of the SND1 protein is required for a proper response of the cell to TNFα regarding the global PC levels. CHPT1 and PTDSS1 participate in the novo synthesis of PC and PS. The physiological role of LPGAT1 is not completely elucidated, but is important in preventing the accumulation of harmful lysophospholipids and in selecting the specific reacylation of the phosphatydilglycerol (71,72). Phosphatydilglycerol is a precursor for cardiolipin biosynthesis and its remodeling may have an impact on mitochondrial membrane properties (73). Lipins are multifunctional lipid metabolism proteins (74). They exhibit phosphatidate phosphohydrolase activity that converts phosphatidate into diacylglycerol and, by controlling the cellular phosphatidate/diacylglycerol ratio, have a role in the net glycerolipid biosynthetic pathway (74). Notably, lipin 1 also functions as a transcriptional coactivator of the hepatic peroxisome proliferator-activated receptor gamma coactivator 1-α (PPARGC1A) which is a key participant in the control of cellular energy and metabolic pathways (75). Because PPARGC1A coactivators are known to be inducible metabolic regulators (75), by compromising TNFα-promoted *LPIN1* overexpression, it is tempting to speculate that SND1 may have a role in energy metabolism and glycerophospholipid biosynthesis.

The impact of SND1 on the transcription of *CHPT1*, *LPGAT1*, *LPIN1* and *PTDSS1*, is provided by silencing the *SND1* gene by interference RNA and determining the expression change of the SND1 targets caused by TNFα. Binding of SND1 to *CHPT1*, *LPGAT1* and *PTDSS1* occurs independently of TNFα stimulation. Our findings suggest that under non-inflammatory conditions, SND1 appears to coactivate *CHPT1* and *LPGAT1* gene expression, as transcript levels significantly diminished (50%) when SND1 was silenced (Figure 5A and B). This is not the case for *PTDSS1*, whose basal expression was unaffected by SND1 silencing. It is quite usual that regions identified by ChIP methods do not have any effect on the expression of the bound gene, as happens in the *PTDSS1* gene. The relevance of the SND1 coactivator is manifested in cells that handle an inflammatory stress. We noted that the glycerolipid gene expression adaptation to TNFα is impaired upon SND1 silencing, given that the changes in the *CHPT1*, *LPGAT1*, *LPIN1* and *PTDSS1* transcripts are abolished upon SND1 depletion (Figure 5). Our results also indicate that SND1 does contribute to sustaining the intracellular content, not only of PC but also of PE in TNFα-treated cells. Knockdown of SND1 resulted in a reduced amount of both PC and PE (Figure 4). This was an unexpected finding that, however, did not impact the PC/PE molar ratio or compromise the rate of cell proliferation. It is plausible that SND1 acts as a co-regulator of unidentified genes and the depletion of SND1 may facilitate the attraction of co-repressor to PE genes tending to decrease the cellular PE levels.

**Figure 6.**
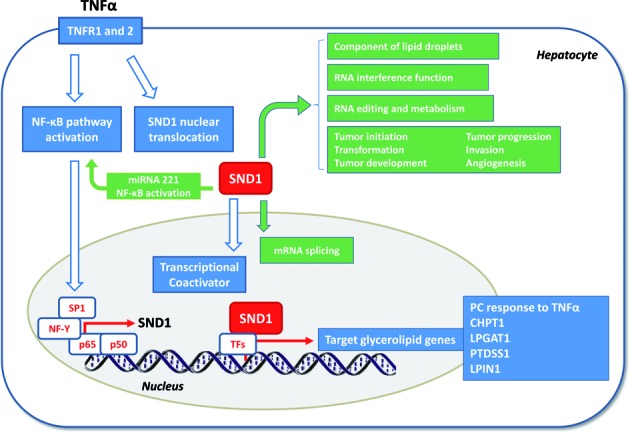
Implication of SND1 in the glycerolipid metabolic response of HepG2 cells to TNFα. *SND1* is an NF-κB responsive gene. Proinflammatory cytokine TNFα activates several signaling pathways by binding to receptors TNFR1 and TNFR2, leading to increased SND1 transcriptional activity via binding of downstream transcription factor NF-κB (6). TNFα promotes the nuclear translocation of the transcriptional coactivator SND1, which binds to a set of target genes involved in glycerophospholipid homeostasis. When SND1 is silenced, SND1 protein does not accumulate into the nucleus and neither the TNFα-induced expression change of target genes nor the increase in cellular phosphatidylcholine levels is elicited. Other SND1 functions are displayed in green. A recent study demonstrated that SND1 initiates a molecular cascade that activates NF-κB, onco-miR-221 and angiogenic factor expression in human HCC (41), whereby the interplay between SND1 and NF-κB activation states might provide an amplification loop mediating the response of hepatocytes to tumorigenic and inflammatory stimuli.

Elucidation of whether or not the gene expression and PC homeostasis are directly related with SND1 binding on promoter targets and the identity of the partner transcription factors requires further study. Nevertheless, the hypothesis (illustrated in Figure 6) is consistent with the observation that TNFα leads to nuclear translocation of the SND1 protein (Figure 3), and increases *SND1* promoter activity via transcription factor NF-κB binding at −116 and −174 sites (6). On the other hand, SND1 seems to initiate a molecular cascade that activates NF-κB, onco-miR-221 and angiogenic factor expression in human HCC (41). It is speculated about the SND1 translocation into the nucleus upon TNFα treatment for interacting with NF-κB and CBP/p300 (76,77). Our results evidence the redistribution of the SND1 protein upon TNFα stimulation (Figure 3), though the interaction of SND1 and NF-κB or CBP/p300 in the nucleus remains to be demonstrated. On the basis of experimental evidence of nuclear interaction between NF-κB and CBP/p300 (60) and the over-representation of the p300 motif we found in the SND1-bound regions, we hypothesize about a potential link between SND1, p300 and NF-κB, and other specific cellular coactivators cooperating for the transcriptional activation of target genes during cell response to inflammatory stimuli (Supplementary Figure S5).

In conclusion, we have identified a broad collection of potential SND1 target genes with functions in transcription regulation, development and regulation of cellular metabolism and validated a subset. Though the exact function of SND1 in mammals remains to be revealed, our findings place SND1 within the TNFα signaling pathway (see Figure 6 for a graphical representation) and postulate that SND1 serves as a modulator of the transcriptional program of glycerophospholipid metabolism during the response to inflammation in human hepatocarcinoma cells.

## Supplementary Material

SUPPLEMENTARY DATA
