# Supplementary material for: Profiling of promoter occupancy by the SND1 transcriptional coactivator identifies downstream glycerolipid metabolic genes involved in TNFα response in human hepatoma cells
Source: Nucleic Acids Res. 2015 Aug 31;43(22):10673–88. doi: 10.1093/nar/gkv858 (PMC4678849; doi:10.1093/nar/gkv858)
Supplement: SUPPLEMENTARY DATA [file supp_43_22_10673__index.html]

Profiling of promoter occupancy by the SND1 transcriptional coactivator identifies downstream glycerolipid metabolic genes involved in TNFα response in human hepatoma cells — SUPPLEMENTARY DATA 

# Profiling of promoter occupancy by the SND1 transcriptional coactivator identifies downstream glycerolipid metabolic genes involved in TNFα response in human hepatoma cells

## SUPPLEMENTARY DATA

- SUPPLEMENTARY DATA
