# Supplementary material for: Profiling of promoter occupancy by the SND1 transcriptional coactivator identifies downstream glycerolipid metabolic genes involved in TNFα response in human hepatoma cells
Source: Nucleic Acids Res. 2015 Aug 31;43(22):10673–88. doi: 10.1093/nar/gkv858 (PMC4678849; doi:10.1093/nar/gkv858)
Supplement: SUPPLEMENTARY DATA [file supp_gkv858_nar-00502-v-2015-File012.pdf]

Supplementary data

Profiling of promoter occupancy by the SND1 transcriptional coactivator identifies downstream glycerolipid metabolic genes involved in TNFα response in human hepatoma cells

Enara Arretxe[1], Sandra Armengol[1], Sarai Mula[1], Yolanda Chico[1], Begoña Ochoa[1] and María José Martínez[1,*]

[1]Department of Physiology, Faculty of Medicine and Dentistry, University of the Basque Country (UPV/EHU), 48940 Leioa, Bizkaia, Spain.

*To whom correspondence should be addressed. Tel: +34 946012832; Fax: +34 946015662; Email: mariajose.martinez@ehu.es.

Supplementary Material and Methods

Cell proliferation was assessed by crystal violet staining. HepG2 cells (9,000/well) were grown in 96 well plates and SND1 or Sp1 siRNA-treated as described in Materials and methods. After 8, 24, 48 or 72 h, cells were washed with phosphate buffered solution, fixed with 4% formaldehyde and stained with 0.25% crystal violet. After drying, 150 µl of 33% acetic acid was added to each well and the absorbance at 590 nm was measured in a Synergy HT Multi-Detection Microplate Reader (BioTek Instruments). Results are reported as means ± SD of 8 replicates from a representative experiment.

Supplementary Results

Supplementary Figure S1. Scheme of the major metabolic pathways to generate glycerolipids in mammalian liver.

This scheme highlights in blue the metabolic roles of the enzymes encoded by the SND1 target genes. *CHPT1, LPGAT1 and LPIN1* transcript upregulations are shown in red and *PTDSS1* downregulation in green.

Supplementary Figure S2. Proliferation of HepG2 cells is not affected by siRNA-mediated SND1 silencing over 72 h.

Proliferation was assessed by crystal violet staining in HepG2 cells grown over 72 h. SND1-silenced cells (▲) showed a proliferation pattern similar to that of cells treated with unspecific siRNA (●) or cells expressing endogenous levels of SND1 (○). Sp1 siRNA treatment (■)

suppressed cell proliferation. Data represent the means ± SD of 8 replicates from a representative experiment.

Supplementary Figure S3. TNFα treatment does not modify the expression of CHPT1 and LPGAT1 protein in cells with basal or residual levels of SND1.

SND1, CHPT1 (45 kDa and 28 kDa isoforms) and LPGAT1 protein levels were determined in the whole cell lysates from control (white bars) and TNFα-treated (50 ng/ml, 8 h) (dark bars) HepG2 cells, expressing either basal (solid bars) or residual levels of SND1 after silencing endogenous SND1 (hatched bars). Results are reported as means ± SD of 3 independent experiments and expressed relative to untreated cells expressing basal levels of SND1. ≠≠≠ $P \leq 0.001$ versus cells expressing endogenous levels of SND1. Representative blots are shown below the graphic.

Supplementary Figure S4. Lack of the TNFα-induced changes on the expression of target genes in SND1 deficient cells.

Transcript expression was determined by reverse transcription quantitative real time PCR in untreated (white bars) and TNFα-treated (50 ng/ml, 8 h) (dark bars) in SND1 silenced HepG2 cells. Results are reported as means ± SD of 3 independent experiments and are expressed relative to the level in untreated cells expressing endogenous levels of SND1, which is shown as a grey grid line. * $P \leq 0.05$, versus cells expressing endogenous levels of SND1.

Supplementary Figure S5. Schematic representation of *CHPT1*, *LPGAT1*, *LPIN1* and *PTDSS1* gene promoters indicating the location of the SND1 binding probes identified by ChIP-chip (light grey) and the region validated by q-PCR (dark grey) from the anti-SND1 immunoprecipitated chromatin. Dotted lines indicate the NF-κB canonical motifs and the solid lines enclose the promoter occupancy annotated in the UCSC Genome Browser (https://genome.ucsc.edu/) for a set of transcription factors overrepresented in the SND1 bound DNA fragments.

Supplementary Table S1. Oligonucleotides used for SND1-binding validation**.**

Supplementary Table S2. Oligonucleotides used for mRNA quantification.

Supplementary Table S3. List of 645 putative SND1 binding genes in control HepG2 cells.

Immunoprecipitated chromatin of control HepG2 cells by anti-SND1 IgG was amplified, labeled and then hybridized into Agilent human promoter microarrays that contained 960,000 oligonucleotide probes covering promoter regions of -9 kb to +2kb of the transcription start site of 21,000 genes. Using the Agilent Feature Extraction Software (ver. 10.7.3.1), we selected the SND1 binding sites that were identified in each of the three replicas. A total of 2,558 probes corresponding to 645 genes were found to be bound to SND1 in the microarrays.

Supplementary Table S4. TNFα-treatment of HepG2 cells promoted SND1 binding to 281 additional genes.

Immunoprecipitated chromatin of TNFα-treated HepG2 cells by anti-SND1 IgG was amplified, labeled and then hybridized into Agilent human promoter microarrays that contained 960,000 oligonucleotide probes covering promoter regions of -9 kb to +2kb of the transcription start site of 21,000 genes. Using the Agilent Feature Extraction Software (ver. 10.7.3.1), we selected the SND1 binding sites that were identified in each of the three replicas. 281 additional genes were found to be bound to SND1 in the microarrays after TNF*α*-treatment.

Supplementary Table S5. TNFα-treatment of HepG2 cells promoted SND1 binding displacement in 104 genes.

Immunoprecipitated chromatin of TNFα-treated HepG2 cells by anti-SND1 IgG was amplified, labeled and then hybridized into Agilent human promoter microarrays that contained 960,000 oligonucleotide probes covering promoter regions of -9 kb to +2kb of the transcription start site of 21,000 genes. Using the Agilent Feature Extraction Software (ver. 10.7.3.1), we selected the SND1 binding sites that were identified in each of the three replicas. The SND1 binding to 104 genes that was detected in control cells was not found after TNFα treatment.

Supplementary Table S6. Lack of SND1 binding to representative target genes in siRNA SND1-treated HepG2 cells.

Immunoprecipitated chromatin of siRNA SND1-treated HepG2 cells by anti-SND1 antibody or non-immune serum IgG was used for qPCR analysis on representative target genes. The SND1 binding to target genes was not detected after silencing SND1 by siRNA treatment with the exception of WNT7B gene (*).

Supplementary Figure S1

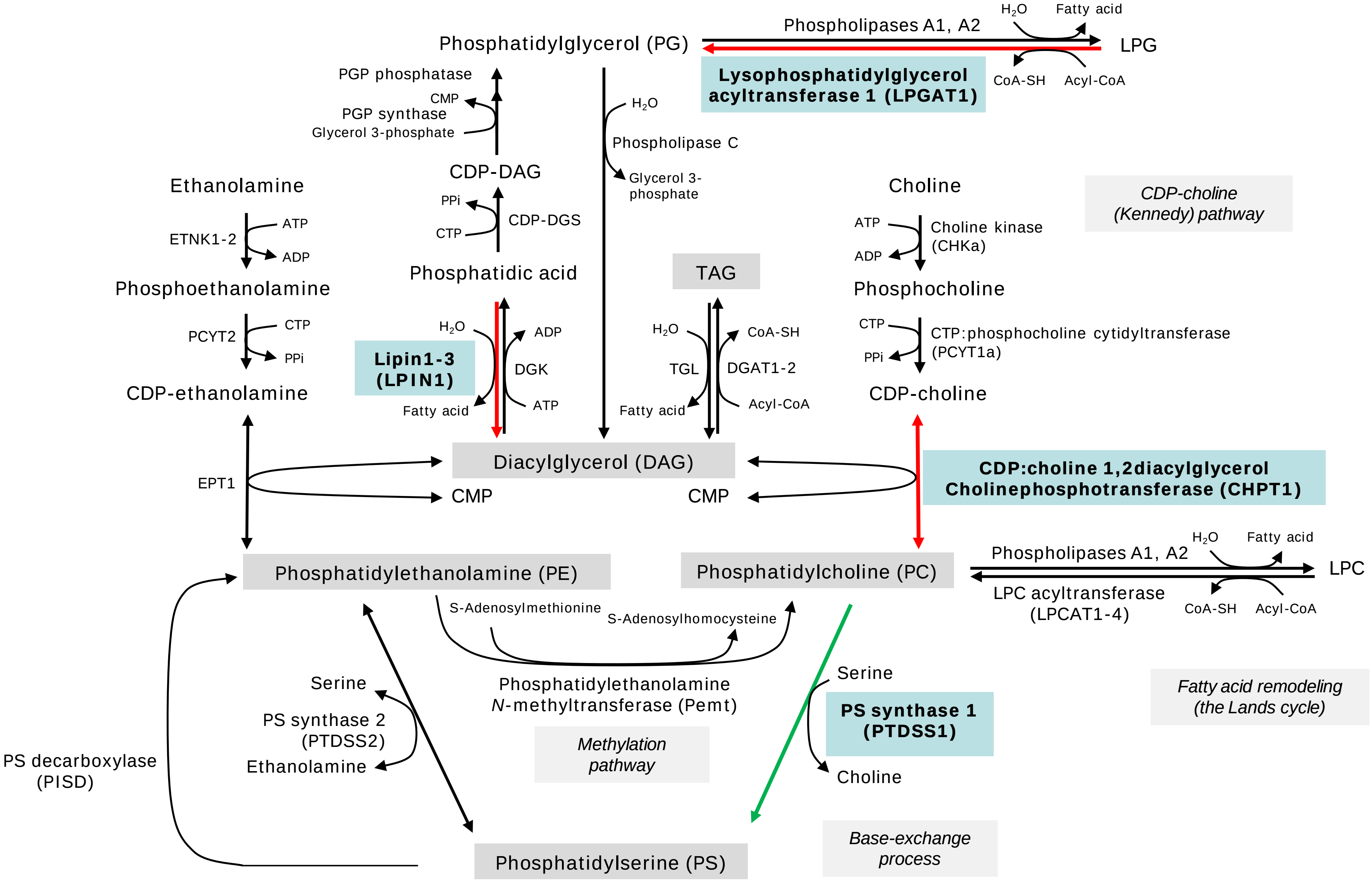

Supplementary Figure S2

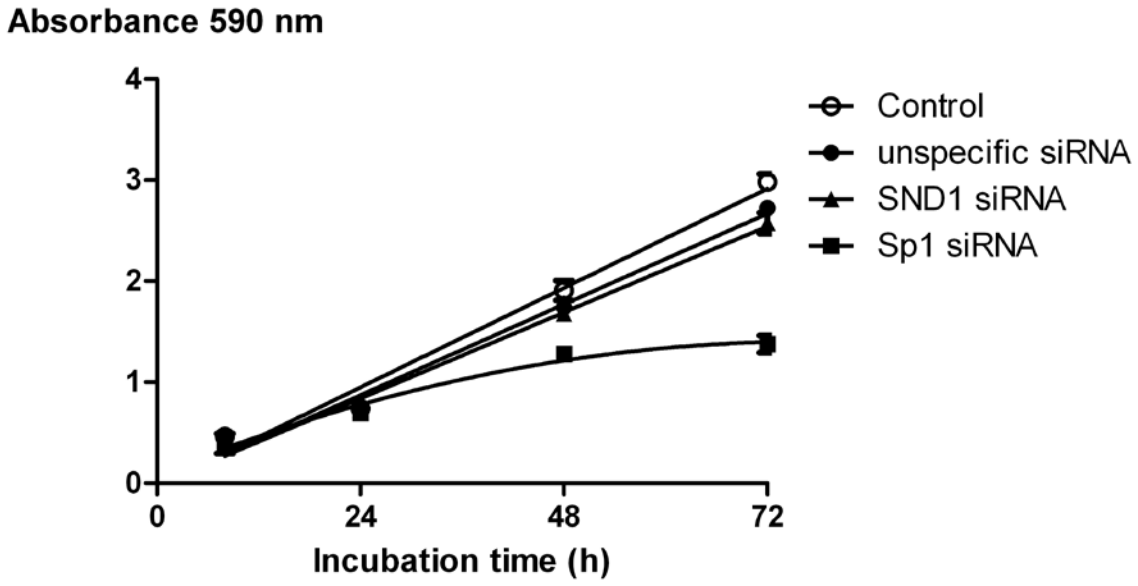

Supplementary Figure S3

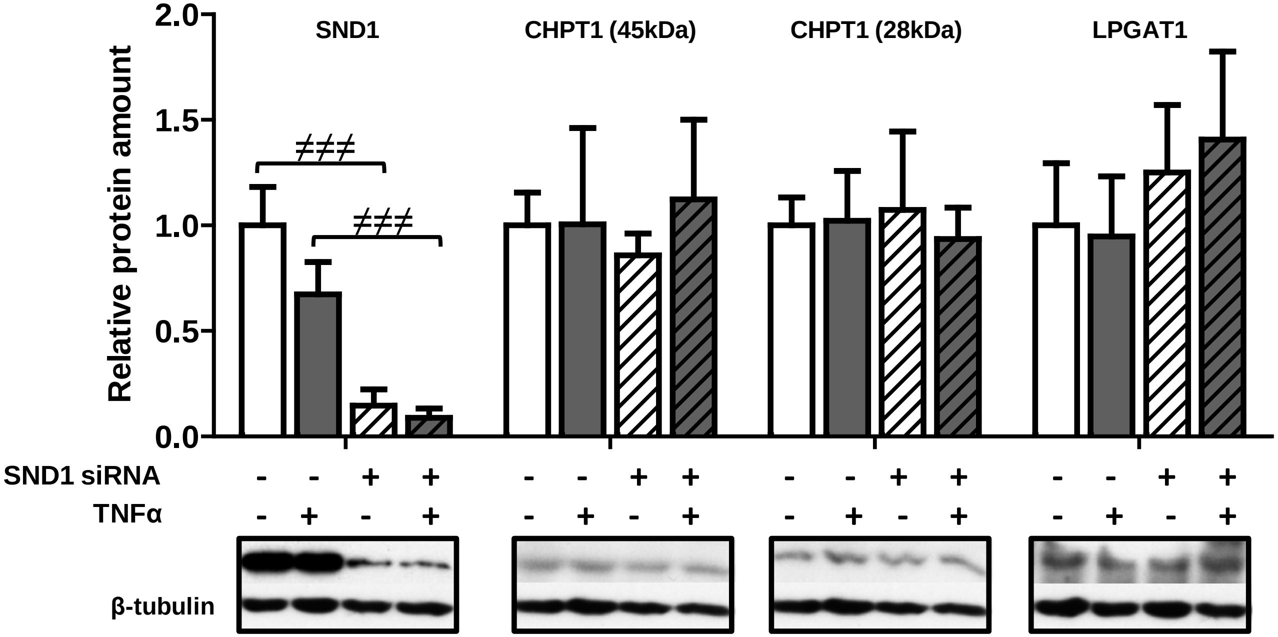

Supplementary Figure S4

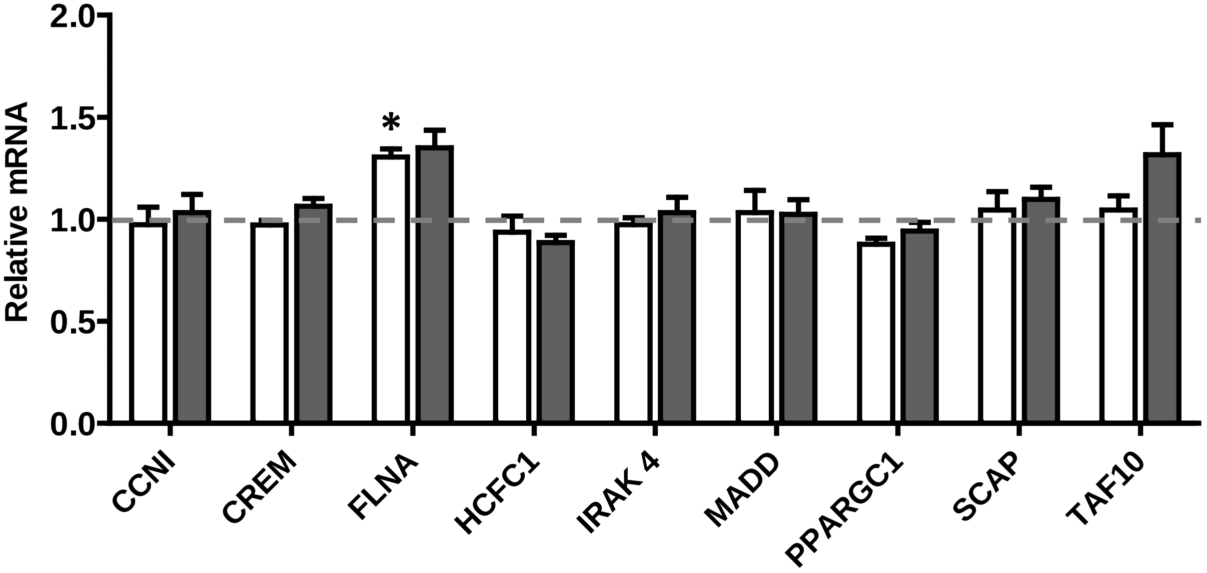

Supplementary Figure S5

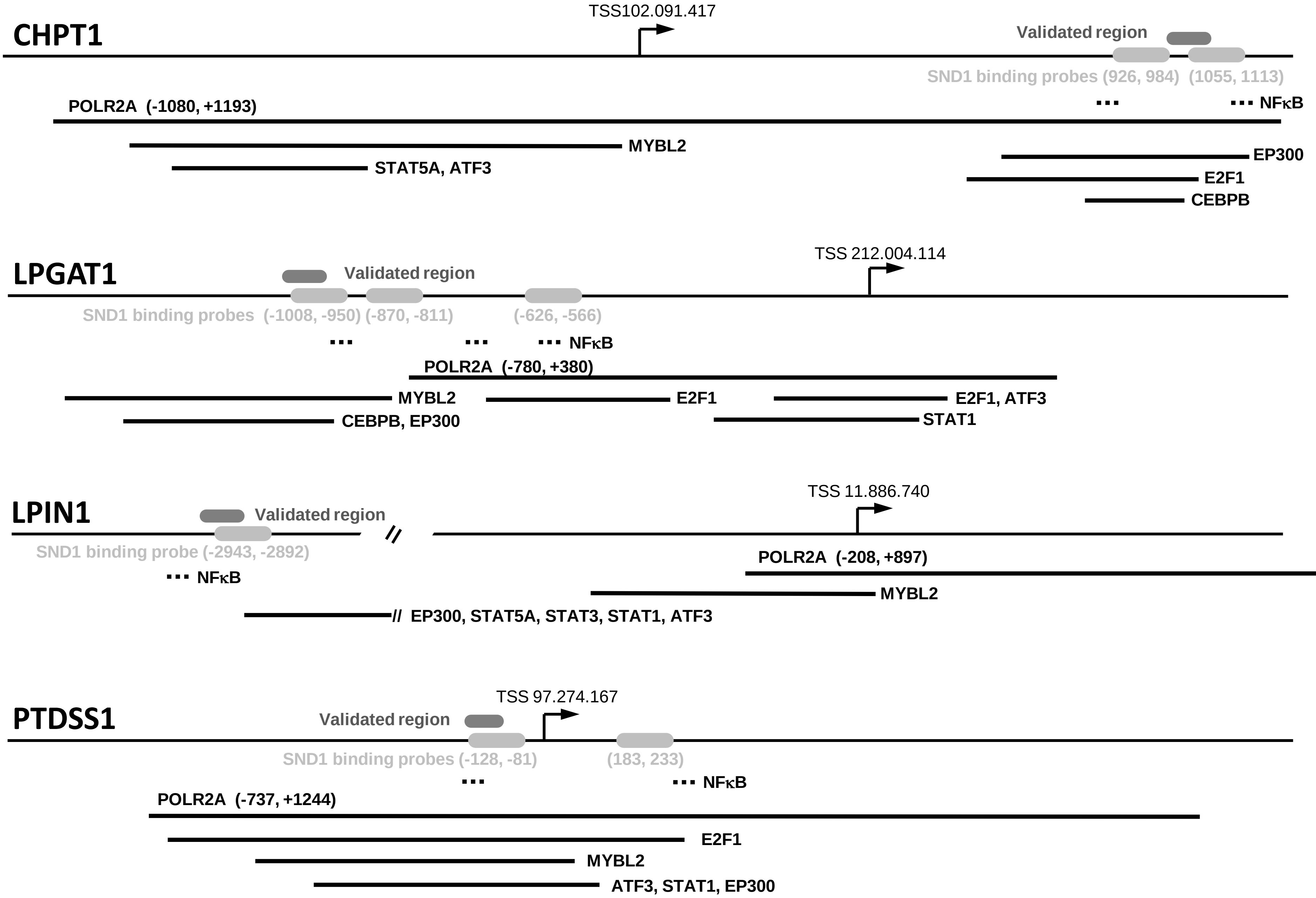

Supplementary Table S1. Oligonucleotides used for SND1-binding validation

GENE NAME	SEQUENCE
ACAA2.s	AAGGTGGACCAAAAGGAGGT
ACAA2.as	ACACAACGGCAAGATGAACA
ADAT1.s	AATGGATCAGGCGCTCAC
ADAT1.as	CGGCCAGTTACAGGATAGGA
ATF1.s	GGCCACATCGTTATGTCTCA
ATF1.as	ATGTTGCTCAGGCTGGTCTT
AZI2.s	TCTCATTTTCCCTGGACCTG
AZI2.as	GCAAAAATTAATATGAAATCACAAGC
BRCA2.s	CCATGCCTGAGAGAAAGGTC
BRCA2.as	GACAGCTGGGAGGGAAGTTA
CALM1.s	GCCTTCTGCCACTAGAGGTG
CALM1.as	TGAAACCTGTAGCACGGTCA
CCND1.s	CTTTCTCCTGACCGACCATC
CCND1.as	TTGGTGACCATTTGGAGACA
CCNI.s	GGCCGCACTTCTCTGTACTT
CCNI.as	CTAGACTCGCCCACTCCTTG
CD36.s	TCAAAGCAGACAGAGCTAGGC
CD36.as	TGGCTCCCTCTCTAGACCTG
CDKN1B.s	GCCTCTAAAAGCGTTGGATG
CDKN1B.as	CCTCCCTTCCCCAAAGTTTA
CHDH.s	CTGTGGCCCTTGAGAACATT
CHDH.as	TGCACTCATCCTCACTCCAG
CHPT1.s	TGCTTCATCGACTTTTTCAAGA
CHPT1.as	AACTCCACTCTGCCCCCTAT
CREM.s	AGTGCACTTCCTCTGCAATG
CREM.as	AGGTTGCACTGAGCCAAGAT
EIF4B.s	CCTGTCTCCGTCCTCACCT
EIF4B.as	CATGTTGGGAGAGGGAAAGA
FADS2.s	ACCTTCAGCTGGGAGGAGAT
FADS2.as	GTGCTGGATGGACCATTTG
FLNA.s	GATGTGGAACAGGGAGAGGA

FLNA.as	TTGGTCCCTCACTTTCTTGG
GK.s	GTTTGTGGCAGTGGCATTC
GK.as	GCCTGGAAAGTGAAAGGCTA
HCFC1.s	AGCCGCTGTTCACTCCTCT
HCFC1.as	CTCCCCGCAAAAGTCTCTC
HOXA.s	CTCCACCCAACTCCCCTATT
HOXA.as	GGGTACGACTTCGAATCACG
HOXB.s	GTAGCTGGGGCTGAGGTTAC
HOXB.as	AAGTTTTTCCGACTGCCTGA
HOXC.s	CCTTGTCGCTTGGAGAAGAC
HOXC.as	ACCCCATATTGGTCCAGACA
HSD17B2.s	CATGCCGTCATTATGTTACCC
HSD17B2.as	GACGTTTGTCTTCCCACTCC
IRAK4.s	GTTCGGCTGGTTCTTCTGTC
IRAK4.as	TGTCAAAGGAACCCACTTCC
LPGAT1.s	AGAGCAAACGCTGTGATGAA
LPGAT1.as	TTTGTAAATGGAGGGGATGG
LPIN1.s	TGGTGCCAGGGATAAAAGTC
LPIN1.as	CCAGGACTCTGAGCATGGTT
LRAP1.s	GATGGGATCGGTTTAGAGCA
LRAP1.as	CGCACCAATTAGGAAGCTGT
MADD.s	AAGTCTCAGGGGCCGATTAT
MADD.as	GGACCCACAGCAGTACCTTC
MBTPS.s	GCACCCCACTTCTTCACACT
MBTPS.as	AAAGCTTGCCTGAAGCATGT
MGLL.s	GCGGAGCTAGTTTCCCAGTT
MGLL.as	CGCTGCGATTCTCCACTACT
NFKB2.s	TTCAGAGAAAGCCAAGCGTTA
NFKB2.as	GAGAGGGGACAGGCTCACTC
PPA2.s	GCTATGGCCCTGTACCACAC
PPA2.as	GGAAGTAAGGTGGCCTGGAC
PPARGC1A.s	ATGCAGGACTGTGTGTGGAG
PPARGC1A.as	CCATTGTGCTGAATGCAAAC
PTDSS1.s	GGGCTACGTGCTCTGAAAAG
PTDSS1.as	TGAGGCATCTCTACCACTCG

PTEN.s	TGCTGCCAGTGTAAAAGTTTG
PTEN.as	CAGTTGCCTTTCCCATGTTA
RXRA.s	CCCTGGGAGGGAAGATAATTT
RXRA.as	TGGGATCAGAATGCTCACAG
SCAP.s	CATTACACAAAGACAAGCAACCA
SCAP.as	AAAGTTTTCTACAAAAAGCACACATT
SETD1A.s	GCGGGCTATTCTCTCACTTG
SETD1A.as	GCCTTGGTGGAGTCAAGAAA
TAF10.s	GGCTGAAACTCCTCCTCCTC
TAF10.as	AGACCTGGCTTCGACGTTTA
TDRD3.s	TTTGACATAAATGGCAGGTTGA
TDRD3.as	AGTTATTCCATTAGACTGTGCAACTG
TRAF7.s	TAGCTGAGGGCCTGAGATTC
TRAF7.as	GATCTTGGGACGAACTGAGC
WNT7B.s	GCTGCTCCGAGTCGTATTTC
WNT7B.as	GTTTCTCCACCCCCACTTC

Supplementary Table S2. Oligonucleotides used for mRNA quantification

GENE NAME	SEQUENCE	AMPLICON SIZE
18S.s	TTCGAACGTCTGCCCTATCAAC	82 bp
18S.as	GAACCCTGATTCCCCGTCAC	
Actin.s	GAGCACAGAGCCTCGCCTTTGCC	84 bp
Actin.as	CGAGCGCGGCGATATCATCATCC	
CCNI.s	ATCTTCACACAGCCACACCA	154 bp
CCNI.as	TTGCAGGCCATACAGTGAAG	
CHPT1.s	CGCTGCAGCTCTACTGGACC	123 bp
CHPT1.as	GACAGTAGGAGATGAGCACGAG	
CREM.s	TGCTACCATGGCAGTACCAA	114 bp
CREM.as	CCCTGAACACCATCAGATCC	
FLNA.s	TGGTGACGAGATCCCCTTCT	95 bp
FLNA.as	CGTGACCTCCGATTGACACT	
GADPH.s	GGTGAAGCAGGCGTCGGAGG	127 bp
GADPH.as	GAGGGCAATGCCAGCCCCAG	
HCFC1.s	CACCACCATCCAGGTCTTG	98 bp
HCFC1.as	CGGTCACTTTGAGAACAGCA	
IRAK4.s	GGCCTGGAGCAGTATCTAGC	103 bp
IRAK4.as	ACATTGAGGCAGCGCACATA	
LPGAT1.s	TGGTGAAAGCACTGATGAGG	117 bp
LPGAT1.as	ACCGCTTACTGTCCAGCACT	
LPIN1.s	GAGCTTCGAGAATGGAATGC	165 bp
LPIN1.as	GGCTGTCCAGCTGTGACTTT	
MADD.s	GAAGTCAAGTGCCCTTCTGC	166 bp
MADD.as	GAATCGAGATGGGTCTGGAA	
PPARGC1A.s	CCTTGCAGCACAAGAAAACA	100 bp
PPARGC1A.as	CTGCTTCGTCGTCAAAAACA	
PTDSS1.s	GCTGGTGGGATCAAGTCATT	108 bp
PTDSS1.as	TTGCCCAGTGGTAAGTCCTC	
SCAP.s	GAGGTCCAGCAGAGGTTGTC	140 bp
SCAP.as	CCTCTTGGCCAGTGTGATGT	

SND1.s	GTGATCAGATACCGGCAGGATG	82 bp
SND1.as	TCTTAATAGCTCTGGCCTCTGCAG	
TAF10.s	GGCCATATCTAACGGGGTTT	132 bp
TAF10.as	ACTGCATCTGGGATCGTAGG	
TBP.s	TTGCAGTGACCCAGCAGCATCAC	136 bp
TBP.as	AACCCTTGCGCTGGAACTCGTC	

Supplementary Table S3

Primary Annotation	Name	P-value	P[Xbar]	FDR	Primary Annotation Type
A1CF	chr10:52618343-52618402	0,07246333	0,009082869	0,039177442	ag_LACat|INSIDE
ABCA3	chr16:2374613-2374659	0,04803557	0,04455205	0,046946541	ag_LACat|INSIDE
ABCF3	chr3:183903332-183903385	0,041898254	0,028792351	0,037482195	ag_LACat|PROMOTER
ABCG1	chr21:43640569-43640621	0,022812633	0,041051604	0,044789861	ag_LACat|INSIDE
ABHD10	chr3:111710497-111710556	0,03299471	0,021059535	0,033314228	ag_LACat|INSIDE
ACAP2	chr3:194999923-194999982	0,040595897	0,03570169	0,041619808	ag_LACat|INSIDE
ACBD5	chr10:27529588-27529635	0,029513996	0,011593597	0,030246199	ag_LACat|INSIDE
ACSL4	chrX:108925024-108925083	0,032663308	0,011445126	0,030473237	ag_LACat|INSIDE
ADAM3A	chr8:39378447-39378506	0,022333205	0,03286093	0,040190967	ag_LACat|INSIDE
ADAMTS8	chr11:130297928-130297972	0,031003352	0,031365566	0,039026002	ag_LACat|INSIDE
ADAMTSL4	chr1:150533361-150533420	0,020923512	0,017928751	0,03101578	ag_LACat|INSIDE
ADAT1	chr16:75657031-75657081	0,08851115	0,0103496	0,031735503	ag_LACat|INSIDE
ADCY8	chr8:132052119-132052168	0,032337856	0,02752075	0,036789102	ag_LACat|INSIDE
ADRA2A	chr10:112835922-112835973	0,030064054	0,033684894	0,040812971	ag_LACat|PROMOTER
ADRBK1	chr11:67048044-67048093	0,028033614	0,025303006	0,035206548	ag_LACat|INSIDE
AFF2	chrX:147584056-147584115	0,035410333	0,014620131	0,029560077	ag_LACat|INSIDE
AFG3L1P	chr16:90038107-90038166	0,027217066	0,034134638	0,040898353	ag_LACat|PROMOTER
AGXT2L2	chr5:177658479-177658526	0,0236862	0,01964594	0,032508755	ag_LACat|INSIDE
AK094279:-3506	chr10:92923399-92923455	0,039088223	0,03932605	0,043868887	ag_LACat|PROMOTER
ALAS1	chr3:52224080-52224139	0,047706373	0,04731461	0,048746103	ag_LACat|PROMOTER
ALB	chr4:74267470-74267529	0,031504016	0,008122012	0,054738977	ag_LACat|PROMOTER
AMACR	chr5:34008504-34008563	0,020578813	0,006937409	0,071246089	ag_LACat|PROMOTER
ANO6	chr12:45610091-45610143	0,023702312	0,010987847	0,030775485	ag_LACat|INSIDE
ANXA10	chr4:169042376-169042435	0,043245558	0,014085828	0,029303957	ag_LACat|INSIDE
AP1S2	chrX:15869734-15869793	0,019492682	0,008104097	0,055193166	ag_LACat|INSIDE
APBB2	chr4:41015100-41015159	0,04965503	0,04697664	0,048630218	ag_LACat|INSIDE
APCDD1L	chr20:57089622-57089666	0,02153372	0,03057029	0,038331352	ag_LACat|INSIDE
ARHGAP22	chr10:49731526-49731570	0,09276065	0,006277597	0,101540125	ag_LACat|INSIDE
ARHGAP31	chr3:119013019-119013063	0,034130353	0,012750607	0,029998701	ag_LACat|PROMOTER
ARHGAP4	chrX:153193402-153193446	0,03215451	0,020260448	0,033186101	ag_LACat|PROMOTER
ARHGAP6	chrX:11684580-11684625	0,04424365	0,019482384	0,032403862	ag_LACat|PROMOTER
ARHGEF3	chr3:56809598-56809653	0,04023816	0,045241274	0,047518026	ag_LACat|INSIDE
ARL11	chr13:50202603-50202647	0,024010217	0,010583344	0,031701035	ag_LACat|INSIDE
ARMCX3	chrX:100876918-100876977	0,023936115	0,008529835	0,045990027	ag_LACat|DIVERGENT_PROMOTER
ARX	chrX:25036141-25036200	0,05959045	0,008539112	0,044554883	ag_LACat|PROMOTER
ASPH	chr8:62601521-62601580	0,046136994	0,027271096	0,036530847	ag_LACat|INSIDE
ASS1	chr9:133349297-133349349	0,0822119	0,010435211	0,031697566	ag_LACat|INSIDE
ATF7IP	chr12:14569122-14569181	0,027041446	0,013636745	0,029706983	ag_LACat|INSIDE
ATP10A	chr15:26108598-26108648	0,033085406	0,00639436	0,091936687	ag_LACat|PROMOTER
ATP4B	chr13:114311298-114311345	0,02877109	0,03858863	0,043496243	ag_LACat|INSIDE
ATP6AP2	chrX:40437595-40437652	0,034495953	0,011868252	0,029878439	ag_LACat|PROMOTER
ATP6V1B2	chr8:20055053-20055101	0,045496754	0,021678872	0,03347549	ag_LACat|INSIDE
AUTS2	chr7:69064121-69064165	0,029390978	0,028011143	0,037061778	ag_LACat|INSIDE
B3GNT2	chr2:62424509-62424568	0,035546344	0,011807266	0,030194866	ag_LACat|INSIDE
BACH2	chr6:91004704-91004763	0,025324564	0,011539372	0,030349487	ag_LACat|INSIDE
BATF	chr14:75981098-75981157	0,04336463	0,04643702	0,048225926	ag_LACat|PROMOTER
BBX	chr3:107428713-107428772	0,04204012	0,010141482	0,032007507	ag_LACat|INSIDE
BCL11A	chr2:60778734-60778786	0,028295247	0,026956616	0,036335272	ag_LACat|INSIDE
BCL2	chr18:60986874-60986930	0,024018427	0,008907365	0,039745277	ag_LACat|PROMOTER
BCL2	chr18:60986874-60986930	0,030922253	0,004358191	0,402821331	ag_LACat|PROMOTER
BCOR	chrX:39940795-39940849	0,036410138	0,02277726	0,033645861	ag_LACat|INSIDE
BIVM	chr13:103450976-103451030	0,06595763	0,009449317	0,036175788	ag_LACat|PROMOTER
BLVRA	chr7:43798378-43798422	0,024060423	0,008867925	0,040691826	ag_LACat|INSIDE
BMF	chr15:40401196-40401240	0,024482986	0,008783496	0,041786191	ag_LACat|PROMOTER
BNC1	chr15:83953895-83953939	0,060343813	0,005696027	0,136493684	ag_LACat|PROMOTER
BNIP3	chr10:133796114-133796158	0,03803513	0,047070824	0,048649877	ag_LACat|PROMOTER
BOC	chr3:112930874-112930931	0,025653811	0,030118065	0,038208604	ag_LACat|PROMOTER
BRCA2	chr13:32889942-32889986	0,043698233	0,034170397	0,040865521	ag_LACat|INSIDE
BTG3	chr21:18984309-18984360	0,026062045	0,006408481	0,090136671	ag_LACat|INSIDE
BUB3	chr10:124910841-124910893	0,044930976	0,04320785	0,046283906	ag_LACat|PROMOTER
C10orf137	chr10:127408096-127408140	0,02981193	0,011367738	0,030645527	ag_LACat|INSIDE
C11orf48	chr11:62439215-62439259	0,028836994	0,00675323	0,074056601	ag_LACat|INSIDE
C11orf73	chr11:86013236-86013281	0,047857974	0,0410926	0,044759111	ag_LACat|PROMOTER
C16orf55	chr16:89724320-89724365	0,039239783	0,043943338	0,046685287	ag_LACat|INSIDE
C16orf91	chr16:1479366-1479419	0,04650722	0,03371502	0,040773118	ag_LACat|PROMOTER
C17orf104	chr17:42734022-42734066	0,09013072	0,00830579	0,049301341	ag_LACat|INSIDE
C18orf18	chr18:5237613-5237672	0,039915692	0,007250368	0,065152612	ag_LACat|INSIDE

C19orf73	chr19:49621665-49621724	0,022057211	0,021814786	0,033366824	ag_LACat|INSIDE
C1orf86	chr1:2137036-2137080	0,028824946	0,005035379	0,217192681	ag_LACat|INSIDE
C20orf7	chr20:13765612-13765662	0,03344415	0,019253675	0,032188961	ag_LACat|DIVERGENT_PROMOTER
C21orf128	chr21:43526251-43526295	0,032100037	0,017580485	0,030909168	ag_LACat|INSIDE
C3orf21	chr3:194992200-194992258	0,023895402	0,011900174	0,029727462	ag_LACat|PROMOTER
C3orf67	chr3:58900335-58900394	0,028017709	0,008276141	0,051487146	ag_LACat|INSIDE
C4orf3	chr4:120221708-120221757	0,0320106	0,045772612	0,047920518	ag_LACat|INSIDE
C4orf38	chr4:184017753-184017808	0,04159637	0,0351692	0,041147328	ag_LACat|DOWNSTREAM
C4orf42	chr4:1243579-1243630	0,016046504	0,006709201	0,074842297	ag_LACat|INSIDE
C5orf42	chr5:37248925-37248984	0,045520995	0,036754888	0,042313901	ag_LACat|INSIDE
C8orf51	chr8:144450533-144450590	0,02452466	0,022088232	0,033468586	ag_LACat|INSIDE
C9orf3	chr9:97766804-97766848	0,09216571	0,008063145	0,056095213	ag_LACat|INSIDE
CA13	chr8:86159097-86159156	0,029742062	0,006944086	0,070200369	ag_LACat|INSIDE
CACNA1D	chr3:53530139-53530194	0,041120674	0,024248652	0,034786869	ag_LACat|INSIDE
CAMTA1	chr1:6844201-6844260	0,027679777	0,00939662	0,03684614	ag_LACat|PROMOTER
CAPN1	chr11:64979120-64979170	0,040126763	0,017386254	0,030903589	ag_LACat|INSIDE
CAPRIN1	chr11:34074692-34074747	0,029478349	0,010912895	0,031104154	ag_LACat|INSIDE
CARS	chr11:3078611-3078655	0,034524515	0,037413087	0,042691829	ag_LACat|INSIDE
CASP2	chr7:142985949-142986008	0,030748868	0,007484688	0,062084531	ag_LACat|INSIDE
CBLB	chr3:105402762-105402821	0,06604489	0,011472405	0,030296514	ag_LACat|INSIDE
CCDC12	chr3:47018483-47018533	0,098604895	0,010266527	0,032089096	ag_LACat|DIVERGENT_PROMOTER
CCDC160	chrX:133371926-133371983	0,042426668	0,016302172	0,030572479	ag_LACat|INSIDE
CCNG1	chr5:162862277-162862336	0,03769612	0,005577383	0,150356939	ag_LACat|PROMOTER
CD164	chr6:109702819-109702878	0,04330256	0,017655356	0,030956681	ag_LACat|INSIDE
CD36	chr7:80259160-80259219	0,04613304	0,012672744	0,030255592	ag_LACat|PROMOTER
CD59	chr11:33746008-33746065	0,044582535	0,03510319	0,0411445	ag_LACat|PROMOTER
CDC14A	chr1:100817099-100817153	0,030096717	0,016399004	0,030228363	ag_LACat|PROMOTER
CDC42EP5	chr19:54976273-54976317	0,04371079	0,030549303	0,038379416	ag_LACat|INSIDE
CDH10	chr5:24592128-24592187	0,056093298	0,008543015	0,044218643	ag_LACat|INSIDE
CDH8	chr16:62069907-62069959	0,04203058	0,007239659	0,06597267	ag_LACat|INSIDE
CDKN1B	chr12:12874155-12874214	0,024011975	0,008619822	0,043232751	ag_LACat|INSIDE
CELSR1	chr22:46933252-46933301	0,020521816	0,00368876	1,193313731	ag_LACat|PROMOTER
CHDH	chr3:53878830-53878885	0,023994617	0,008363069	0,048311658	ag_LACat|INSIDE
CHPT1	chr12:102092342-102092400	0,025981164	0,042958774	0,046169978	ag_LACat|INSIDE
CHRNB1	chr17:7348613-7348657	0,04507865	0,015056664	0,029974343	ag_LACat|INSIDE
CHST8	chr19:34175381-34175431	0,027439266	0,01317939	0,029711029	ag_LACat|PROMOTER
CLCN5	chrX:49823835-49823894	0,023033844	0,038263626	0,04328071	ag_LACat|PROMOTER
CLDN2	chrX:106158065-106158124	0,028245648	0,044384442	0,046922768	ag_LACat|PROMOTER
CLK4	chr5:178054523-178054576	0,03217118	0,011403924	0,030615514	ag_LACat|PROMOTER
CLSTN1	chr1:9884671-9884723	0,024647618	0,009353048	0,037125289	ag_LACat|PROMOTER
CNOT8	chr5:154237614-154237661	0,022417719	0,003970654	0,85633767	ag_LACat|DIVERGENT_PROMOTER
CNPY1	chr7:155326394-155326453	0,029927526	0,006046685	0,12225642	ag_LACat|INSIDE
COL11A1	chr1:103574302-103574346	0,044275004	0,029570015	0,03795992	ag_LACat|PROMOTER
COL13A1	chr10:71561624-71561669	0,02321344	0,01382067	0,029609184	ag_LACat|INSIDE
COL14A1	chr8:121137499-121137543	0,0404396	0,02303761	0,033798943	ag_LACat|INSIDE
COL23A1	chr5:178017381-178017427	0,03595299	0,015256506	0,030002916	ag_LACat|INSIDE
COL28A1	chr7:7574944-7575003	0,021349391	0,03707819	0,042534732	ag_LACat|INSIDE
COPE	chr19:19013726-19013776	0,02402181	0,045952607	0,048031239	ag_LACat|INSIDE
CREM	chr10:35427394-35427453	0,03563259	0,008531002	0,045616184	ag_LACat|INSIDE
CRHR2	chr7:30734131-30734184	0,03684843	0,047546707	0,048752329	ag_LACat|INSIDE
CXCR3	chrX:70842806-70842850	0,032510165	0,038956314	0,043682383	ag_LACat|PROMOTER
CXCR4	chr2:136875284-136875332	0,030371906	0,019813491	0,032702369	ag_LACat|PROMOTER
CXorf22	chrX:35932716-35932775	0,01936389	0,004867182	0,242235884	ag_LACat|PROMOTER
CXorf30	chrX:36320348-36320407	0,03916404	0,016171964	0,03059433	ag_LACat|INSIDE
CXorf36	chrX:45059737-45059793	0,030698046	0,033274554	0,040391438	ag_LACat|INSIDE
CXorf41	chrX:106449741-106449792	0,03184843	0,022442674	0,033380253	ag_LACat|DIVERGENT_PROMOTER
CXorf59	chrX:36077064-36077123	0,058989942	0,00960261	0,034708875	ag_LACat|INSIDE
CYBB	chrX:37634810-37634869	0,041925684	0,016144315	0,030631589	ag_LACat|PROMOTER
CYorf15A	chrY:21749977-21750036	0,056193102	0,005624868	0,145571571	ag_LACat|INSIDE
CYorf15B	chrY:21753518-21753577	0,032864597	0,009041568	0,039261037	ag_LACat|PROMOTER
CYP26C1	chr10:94819936-94819984	0,0425245	0,038724467	0,043573444	ag_LACat|PROMOTER
DACH2	chrX:85404189-85404248	0,047673482	0,010314406	0,031778194	ag_LACat|INSIDE
DALRD3	chr3:49056025-49056070	0,02451198	0,011071353	0,030611818	ag_LACat|PROMOTER
DCAF12L2	chrX:125302938-125302997	0,09572153	0,012733601	0,030068029	ag_LACat|PROMOTER
DCAF6	chr1:167919746-167919805	0,042417813	0,049356177	0,049663214	ag_LACat|INSIDE
DDIT4	chr10:74033012-74033056	0,020253154	0,013159398	0,029874142	ag_LACat|PROMOTER
DDX26B	chrX:134656046-134656100	0,025518803	0,006356204	0,095638703	ag_LACat|INSIDE
DDX3Y	chrY:15013384-15013443	0,040368643	0,007525357	0,061631725	ag_LACat|PROMOTER
DENND1B	chr1:197706953-197707011	0,04526412	0,017702626	0,030872235	ag_LACat|INSIDE

DISC1	chr1:231762284-231762337	0,026255071	0,016434848	0,030122795	ag_LACat|PROMOTER
DKK1	chr10:54071713-54071772	0,0479382	0,02036743	0,033026885	ag_LACat|PROMOTER
DLEU1	chr13:50655482-50655530	0,04838088	0,009809598	0,032715515	ag_LACat|PROMOTER
DLEU2	chr13:50704687-50704741	0,045507085	0,028264416	0,037168856	ag_LACat|PROMOTER
DMD	chrX:31287021-31287080	0,035750262	0,017830295	0,030928152	ag_LACat|PROMOTER
DMRT2	chr9:1045552-1045604	0,020004146	0,035202872	0,041112379	ag_LACat|PROMOTER
DNAJC24	chr11:31393874-31393933	0,02252908	0,01594927	0,030530112	ag_LACat|INSIDE
DNTTIP2	chr1:94344390-94344449	0,031855505	0,036637455	0,042253892	ag_LACat|INSIDE
DOCK9	chr13:99739595-99739639	0,048225064	0,009546915	0,035296309	ag_LACat|PROMOTER
DOPEY2	chr21:37529681-37529725	0,044461947	0,040637616	0,044563623	ag_LACat|PROMOTER
DPF3	chr14:73243642-73243698	0,04252558	0,020891173	0,033374294	ag_LACat|INSIDE
DUSP16	chr12:12715065-12715109	0,033522762	0,016903128	0,030463298	ag_LACat|INSIDE
DUSP3	chr17:41856570-41856619	0,05044129	0,005939766	0,12810096	ag_LACat|DIVERGENT_PROMOTER
DYRK1A	chr21:38736879-38736938	0,04020938	0,021279374	0,033175313	ag_LACat|PROMOTER
DZIP3	chr3:108308160-108308205	0,039718382	0,00981458	0,032564273	ag_LACat|PROMOTER
EBF3	chr10:131769001-131769046	0,03836768	0,022481363	0,033361105	ag_LACat|PROMOTER
EDA	chrX:68835300-68835347	0,033086997	0,010517666	0,031798738	ag_LACat|PROMOTER
EEF2	chr19:3982404-3982448	0,031477984	0,021622073	0,033467658	ag_LACat|INSIDE
EFNA2	chr19:1282850-1282906	0,015969427	0,020278417	0,033131656	ag_LACat|PROMOTER
EFNB2	chr13:107186339-107186387	0,023208827	0,01595162	0,030444537	ag_LACat|INSIDE
EGFLAM	chr5:38257950-38257994	0,022697378	0,006510623	0,087757773	ag_LACat|PROMOTER
EIF1AD	chr11:65769870-65769929	0,031721793	0,014676041	0,029580681	ag_LACat|PROMOTER
EIF1AY	chrY:22737477-22737526	0,03478578	0,012677788	0,030156356	ag_LACat|PROMOTER
EIF2A	chr3:150264488-150264532	0,023577757	0,008536395	0,044902826	ag_LACat|DIVERGENT_PROMOTER
EIF4EBP1	chr8:37889419-37889473	0,049636595	0,030481648	0,038368923	ag_LACat|INSIDE
EIF4G3	chr1:21332595-21332654	0,04142666	0,012670535	0,030362356	ag_LACat|INSIDE
ELL	chr19:18555258-18555302	0,09518721	0,010266325	0,032244234	ag_LACat|INSIDE
EMX2OS	chr10:119304642-119304701	0,036396362	0,03585342	0,041571976	ag_LACat|PROMOTER
ENKUR	chr10:25305029-25305074	0,04169073	0,020996843	0,033378274	ag_LACat|DIVERGENT_PROMOTER
ENST00000516501:156	chr7:148638706-148638765	0,047123674	0,014425485	0,029442551	ag_LACat|DOWNSTREAM
ENST00000516501:285	chr7:148638835-148638894	0,030306205	0,038006604	0,043140829	ag_LACat|DOWNSTREAM
ENTPD5	chr14:74485274-74485324	0,0385141	0,039160464	0,04375962	ag_LACat|INSIDE
EPB41L4A	chr5:111755094-111755138	0,09437824	0,009846219	0,032337582	ag_LACat|DIVERGENT_PROMOTER
EPB49	chr8:21911790-21911834	0,028496712	0,019504352	0,03235722	ag_LACat|PROMOTER
ERCC5	chr13:103498085-103498135	0,042103585	0,013141591	0,029938765	ag_LACat|PROMOTER
ESR2	chr14:64761605-64761654	0,038777966	0,009232198	0,038046064	ag_LACat|PROMOTER
ESRP2	chr16:68271322-68271366	0,056313027	0,007425144	0,062390496	ag_LACat|DIVERGENT_PROMOTER
ESX1	chrX:103498766-103498810	0,023614116	0,033129107	0,040366351	ag_LACat|INSIDE
ETV6	chr12:11802308-11802357	0,021324625	0,004577379	0,329062669	ag_LACat|PROMOTER
EVX2	chr2:176948271-176948317	0,033519376	0,022441871	0,033455969	ag_LACat|INSIDE
EXOG	chr3:38537831-38537875	0,025604306	0,00464893	0,300785771	ag_LACat|INSIDE
EYS	chr6:66290468-66290527	0,029087491	0,031428706	0,039029506	ag_LACat|PROMOTER
FADS2	chr11:61595970-61596015	0,036005046	0,009661585	0,034158719	ag_LACat|INSIDE
FAM122C	chrX:133938956-133939015	0,034503307	0,008819241	0,041348181	ag_LACat|DIVERGENT_PROMOTER
FAM123A	chr13:25744623-25744682	0,04515108	0,022997772	0,033817178	ag_LACat|INSIDE
FAM125B	chr9:129088411-129088470	0,029782966	0,023591159	0,034454808	ag_LACat|PROMOTER
FAM133A	chrX:92936324-92936383	0,04473383	0,022321548	0,033508217	ag_LACat|INSIDE
FAM155A	chr13:108519151-108519209	0,038938552	0,028646788	0,037367887	ag_LACat|INSIDE
FAM155B	chrX:68725560-68725609	0,019397844	0,021004507	0,033308618	ag_LACat|INSIDE
FAM164C	chr14:75536070-75536122	0,029747346	0,01230171	0,02980976	ag_LACat|DIVERGENT_PROMOTER
FAM195A	chr16:696194-696250	0,014780334	0,03017434	0,038205084	ag_LACat|INSIDE
FAM46D	chrX:79590672-79590722	0,044652753	0,049798828	0,049798828	ag_LACat|PROMOTER
FAM53B	chr10:126432398-126432457	0,039710507	0,0380302	0,043092013	ag_LACat|INSIDE
FAM5C	chr1:190425252-190425311	0,034133807	0,007686773	0,060650514	ag_LACat|INSIDE
FAM82B	chr8:87493220-87493279	0,030064778	0,00784557	0,058345791	ag_LACat|INSIDE
FAM84B	chr8:127569345-127569389	0,041859593	0,010622143	0,031670629	ag_LACat|INSIDE
FANCB	chrX:14884214-14884273	0,035662826	0,009182161	0,038328117	ag_LACat|INSIDE
FAT4	chr4:126235929-126235973	0,028033614	0,009781428	0,033133947	ag_LACat|PROMOTER
FCHO2	chr5:72250796-72250855	0,02228814	0,029259399	0,03778609	ag_LACat|PROMOTER
FGF13	chrX:137820451-137820510	0,027962266	0,013716111	0,029482803	ag_LACat|INSIDE
FGF14	chr13:102568822-102568866	0,029891199	0,048638895	0,049558055	ag_LACat|INSIDE
FGL1	chr8:17752869-17752928	0,024102053	0,047251202	0,048758417	ag_LACat|INSIDE
FHL1	chrX:135225915-135225971	0,02924275	0,01869242	0,031494781	ag_LACat|PROMOTER
FKTN	chr9:108319832-108319891	0,0351471	0,033254907	0,040443468	ag_LACat|PROMOTER
FLI1	chr11:128559854-128559912	0,025677571	0,015730986	0,030381934	ag_LACat|PROMOTER
FLJ34503	chr6:114224292-114224349	0,027944727	0,004219223	0,454972913	ag_LACat|PROMOTER
FLJ45983	chr10:8091779-8091824	0,08078633	0,007278086	0,064505773	ag_LACat|DOWNSTREAM
FMR1-AS1	chrX:147007320-147007379	0,046706174	0,014156735	0,029170088	ag_LACat|PROMOTER
FNDC3A	chr13:49579206-49579265	0,020814057	0,012829664	0,029751945	ag_LACat|INSIDE

FOSL2	chr2:28616068-28616121	0,022381598	0,016672632	0,030216227	ag_LACat|INSIDE
FOXB1	chr15:60296424-60296476	0,029621547	0,015171181	0,029926079	ag_LACat|INSIDE
FOXE1	chr9:100610171-100610222	0,021421976	0,03902308	0,043681545	ag_LACat|PROMOTER
FOXL2	chr3:138663662-138663721	0,022275187	0,033836905	0,040692337	ag_LACat|INSIDE
FOXO4	chrX:70316821-70316870	0,045185897	0,02242508	0,033508145	ag_LACat|INSIDE
FOXP1	chr3:71104270-71104329	0,04511938	0,029207693	0,037794755	ag_LACat|INSIDE
FSCB	chr14:44974478-44974527	0,032185897	0,006054474	0,118704376	ag_LACat|INSIDE
FST	chr5:52771640-52771699	0,029152729	0,032252125	0,039746905	ag_LACat|PROMOTER
FXR2	chr17:7518355-7518400	0,028443545	0,008185005	0,053491901	ag_LACat|PROMOTER
G3BP2	chr4:76598920-76598979	0,025619414	0,006417799	0,088347148	ag_LACat|PROMOTER
GABRA1	chr5:161278165-161278224	0,034027457	0,010944141	0,0310564	ag_LACat|INSIDE
GAD2	chr10:26506883-26506932	0,035656232	0,02128541	0,033104953	ag_LACat|INSIDE
GALNT9	chr12:132905265-132905309	0,028607054	0,028399946	0,037271329	ag_LACat|INSIDE
GAS2	chr11:22681080-22681139	0,048647616	0,009535808	0,035457861	ag_LACat|PROMOTER
GATA4	chr8:11554911-11554968	0,030600473	0,04907969	0,049616499	ag_LACat|PROMOTER
GCAT	chr22:38203618-38203662	0,03988873	0,009902396	0,032357829	ag_LACat|PROMOTER
GDAP1	chr8:75262767-75262819	0,027253052	0,005729938	0,132402496	ag_LACat|INSIDE
GEMIN8	chrX:14039822-14039881	0,031229274	0,02110659	0,033226189	ag_LACat|INSIDE
GFOD1	chr6:13488126-13488173	0,031897347	0,009798369	0,033018462	ag_LACat|PROMOTER
GGT6	chr17:4462386-4462431	0,028165706	0,021500045	0,033358583	ag_LACat|INSIDE
GK	chrX:30733357-30733416	0,021742936	0,005871069	0,130985574	ag_LACat|INSIDE
GNB2	chr7:100272299-100272343	0,023794748	0,027090447	0,036364148	ag_LACat|INSIDE
GNG12	chr1:68299331-68299377	0,021173216	0,03161768	0,039188964	ag_LACat|PROMOTER
GOLGA3	chr12:133405508-133405552	0,02135587	0,016172003	0,030505207	ag_LACat|PROMOTER
GOLGA4	chr3:37285024-37285070	0,026763659	0,024190057	0,034779926	ag_LACat|INSIDE
GPC3	chrX:133118320-133118374	0,048406996	0,008293618	0,05014926	ag_LACat|INSIDE
GPC4	chrX:132547985-132548041	0,025458556	0,006620516	0,0764906	ag_LACat|INSIDE
GPR174	chrX:78425724-78425783	0,035782937	0,018528588	0,031547359	ag_LACat|PROMOTER
GRIA2	chr4:158138421-158138480	0,027512748	0,00782909	0,058900247	ag_LACat|PROMOTER
GRID2	chr4:93219782-93219841	0,022931257	0,029465618	0,037901103	ag_LACat|PROMOTER
GRM8	chr7:126892397-126892454	0,09885625	0,011467909	0,030408757	ag_LACat|PROMOTER
GRPEL1	chr4:7069291-7069335	0,029339923	0,007374064	0,062776568	ag_LACat|INSIDE
GSC	chr14:95236613-95236669	0,02083274	0,005355266	0,164993208	ag_LACat|PROMOTER
GSN	chr9:124061742-124061801	0,024333937	0,008330782	0,049000145	ag_LACat|PROMOTER
GTDC1	chr2:145089435-145089479	0,0660386	0,006974782	0,06942591	ag_LACat|INSIDE
GTF2A1	chr14:81686956-81687014	0,03355828	0,023891244	0,034658374	ag_LACat|INSIDE
GUCA2A	chr1:42629113-42629158	0,04167965	0,024865657	0,035050283	ag_LACat|INSIDE
GUCY2F	chrX:108717303-108717362	0,06622255	0,005170552	0,176070902	ag_LACat|INSIDE
H2AFJ	chr12:14932078-14932137	0,034531265	0,035963677	0,041625222	ag_LACat|DOWNSTREAM
H2AFY	chr5:134734502-134734557	0,02229549	0,005047513	0,204108807	ag_LACat|INSIDE
HAAO	chr2:43020171-43020215	0,036847025	0,008543624	0,043870831	ag_LACat|PROMOTER
HCFC1	chrX:153214604-153214663	0,022073846	0,00890524	0,040011738	ag_LACat|INSIDE
HERC3	chr4:89513155-89513200	0,04421265	0,022192603	0,033548164	ag_LACat|PROMOTER
HEY2	chr6:126068754-126068802	0,057572436	0,008197822	0,053039908	ag_LACat|PROMOTER
HHAT	chr1:210502816-210502870	0,048918534	0,00767106	0,061273776	ag_LACat|INSIDE
HIST2H2AB	chr1:149859172-149859217	0,057187717	0,005051586	0,19225742	ag_LACat|INSIDE
HLX	chr1:221052531-221052575	0,02541694	0,025059722	0,035094459	ag_LACat|PROMOTER
HMGN5	chrX:80377530-80377578	0,036701303	0,013609287	0,029747327	ag_LACat|INSIDE
HMX3	chr10:124895184-124895242	0,038526345	0,008532772	0,045251668	ag_LACat|PROMOTER
HNF1B	chr17:36103906-36103950	0,026951043	0,008028018	0,057078326	ag_LACat|INSIDE
HNRNPH2	chrX:100663150-100663194	0,061603416	0,008471527	0,04684682	ag_LACat|INSIDE
HOXA3	chr7:27150234-27150281	0,02942115	0,041732956	0,045152546	ag_LACat|INSIDE
HOXB3	chr17:46628587-46628646	0,039206915	0,024048995	0,034731473	ag_LACat|INSIDE
HOXB9	chr17:46703841-46703892	0,047205105	0,04927051	0,049654237	ag_LACat|PROMOTER
HOXC10	chr12:54378317-54378374	0,032723434	0,024667948	0,035000356	ag_LACat|PROMOTER
HOXC9	chr12:54390939-54390988	0,036504716	0,009409591	0,036455122	ag_LACat|PROMOTER
HSD17B2	chr16:82065015-82065074	0,020538813	0,016306477	0,030492169	ag_LACat|PROMOTER
HSPA4L	chr4:128704022-128704066	0,042910542	0,021245915	0,033283552	ag_LACat|INSIDE
HSPG2	chr1:22181188-22181232	0,023236094	0,018610807	0,031439144	ag_LACat|INSIDE
HTN3	chr4:70894498-70894557	0,018654928	0,03499745	0,041244718	ag_LACat|INSIDE
HTR2C	chrX:113949784-113949843	0,029674536	0,048882842	0,049650234	ag_LACat|INSIDE
ID3	chr1:23886397-23886456	0,024611255	0,040331293	0,04437814	ag_LACat|PROMOTER
IFNA8	chr9:21402295-21402354	0,029245283	0,018549228	0,031499608	ag_LACat|PROMOTER
IGBP1	chrX:69352421-69352480	0,06820797	0,009122834	0,038327751	ag_LACat|PROMOTER
IGF2	chr11:2165284-2165343	0,038026225	0,01039689	0,031730131	ag_LACat|PROMOTER
IGSF1	chrX:130429313-130429372	0,04356686	0,028583545	0,037360714	ag_LACat|PROMOTER
IL1RAPL1	chrX:28600454-28600511	0,045337588	0,01539786	0,030189138	ag_LACat|PROMOTER
ILF3	chr19:10764734-10764781	0,035334487	0,044477258	0,046944186	ag_LACat|DIVERGENT_PROMOTER
IPO11	chr5:61708607-61708651	0,018437799	0,01471067	0,029558396	ag_LACat|PROMOTER

IPO5	chr13:98627854-98627913	0,03188177	0,041967474	0,045330477	ag_LACat|INSIDE
IQCB1	chr3:121553693-121553737	0,026116366	0,008643957	0,04269191	ag_LACat|INSIDE
IRAK4	chr12:44152820-44152864	0,05096707	0,00454197	0,367331824	ag_LACat|INSIDE
IRF2BP2	chr1:234746108-234746152	0,033752356	0,040158525	0,044263315	ag_LACat|PROMOTER
IRF6	chr1:209961574-209961633	0,04128171	0,041197214	0,044722479	ag_LACat|INSIDE
IRS4	chrX:107986687-107986746	0,026137764	0,00537952	0,158206793	ag_LACat|PROMOTER
IRX2	chr5:2743358-2743409	0,023362309	0,034440584	0,040961503	ag_LACat|DOWNSTREAM
IRX3	chr16:54318007-54318054	0,02990489	0,009383455	0,037018874	ag_LACat|INSIDE
ISLR2	chr15:74425248-74425292	0,030064054	0,03072371	0,038374981	ag_LACat|INSIDE
IZUMO4	chr19:2099303-2099347	0,035389163	0,031230424	0,038932725	ag_LACat|INSIDE
JPH3	chr16:87635582-87635627	0,040773373	0,020282095	0,032971144	ag_LACat|PROMOTER
JUNB	chr19:12903363-12903407	0,028592788	0,02991905	0,038030698	ag_LACat|INSIDE
JUP	chr17:39943366-39943410	0,02374166	0,007822165	0,059540479	ag_LACat|PROMOTER
KCNC2	chr12:75602975-75603026	0,041984703	0,039389007	0,04386349	ag_LACat|INSIDE
KCNQ1DN	chr11:2889652-2889711	0,029002812	0,006118516	0,116431751	ag_LACat|PROMOTER
KCNS3	chr2:18060348-18060394	0,03141217	0,006561023	0,086632283	ag_LACat|INSIDE
KCTD15	chr19:34286169-34286215	0,051581834	0,008444581	0,047100379	ag_LACat|PROMOTER
KDM5D	chrY:21906414-21906473	0,06909526	0,010977311	0,030879653	ag_LACat|INSIDE
KIAA1704	chr13:45563722-45563775	0,025487553	0,026040828	0,035620329	ag_LACat|INSIDE
KIAA2026	chr9:5930609-5930668	0,049145393	0,009709749	0,033594693	ag_LACat|INSIDE
KIF7	chr15:90198321-90198367	0,029060796	0,017359734	0,030941454	ag_LACat|INSIDE
KITLG	chr12:88972585-88972644	0,040129	0,011924652	0,029560344	ag_LACat|INSIDE
KLHL13	chrX:117080143-117080202	0,03126633	0,04948231	0,049712818	ag_LACat|INSIDE
KLHL14	chr18:30352323-30352371	0,03909523	0,015786657	0,030308508	ag_LACat|INSIDE
KLHL2	chr4:166243090-166243149	0,045817558	0,007016653	0,067757828	ag_LACat|INSIDE
KLHL4	chrX:86764458-86764517	0,027939253	0,046280507	0,048218177	ag_LACat|PROMOTER
KLHL9	chr9:21334894-21334953	0,025100652	0,00565061	0,140613257	ag_LACat|INSIDE
KRAS	chr12:25403911-25403955	0,06180022	0,004753203	0,279574758	ag_LACat|PROMOTER
KTI12	chr1:52498223-52498282	0,042990215	0,013908952	0,029408797	ag_LACat|INSIDE
L3MBTL3	chr6:130341544-130341600	0,07766257	0,006588224	0,083580018	ag_LACat|INSIDE
LAMB1	chr7:107643274-107643324	0,020829586	0,00849532	0,046188841	ag_LACat|INSIDE
LAPTM4A	chr2:20251246-20251290	0,04268867	0,006242867	0,106293018	ag_LACat|INSIDE
LARS2	chr3:45430035-45430083	0,046121273	0,008905062	0,040290735	ag_LACat|PROMOTER
LBH	chr2:30455545-30455589	0,037389707	0,02078179	0,033364313	ag_LACat|INSIDE
LCOR	chr10:98591469-98591528	0,030657113	0,011924867	0,029448049	ag_LACat|PROMOTER
LCTL	chr15:66859822-66859870	0,021437922	0,023624232	0,034425401	ag_LACat|PROMOTER
LMNB1	chr5:126111981-126112040	0,029358976	0,00923199	0,038289087	ag_LACat|PROMOTER
LOC100132831	chrX:40691467-40691515	0,045099042	0,008353467	0,048690929	ag_LACat|INSIDE
LOC100329135	chrX:111126799-111126858	0,082765445	0,008856885	0,040931461	ag_LACat|INSIDE
LOC158572	chrX:49641685-49641737	0,04678282	0,013287589	0,029645069	ag_LACat|INSIDE
LOC286094	chr8:136246391-136246450	0,036554333	0,011908302	0,029633352	ag_LACat|INSIDE
LOC286467	chrX:130963436-130963495	0,061737042	0,009565505	0,034965434	ag_LACat|INSIDE
LOC441204	chr7:26438015-26438069	0,044785965	0,027083473	0,036430368	ag_LACat|PROMOTER
LOC642006	chr7:57247594-57247638	0,043123443	0,02508302	0,035051218	ag_LACat|INSIDE
LOC90834	chr22:50181118-50181165	0,025079485	0,030469256	0,038428087	ag_LACat|PROMOTER
LOC92249	chrX:62781259-62781318	0,032052267	0,0094019	0,036644755	ag_LACat|PROMOTER
LONP1	chr19:5719703-5719747	0,037355434	0,008700458	0,042645427	ag_LACat|INSIDE
LPGAT1	chr1:212004925-212004984	0,041128144	0,012596597	0,030297391	ag_LACat|PROMOTER
LPXN	chr11:58344825-58344884	0,029915402	0,006934054	0,072360206	ag_LACat|PROMOTER
LRCH1	chr13:47125433-47125492	0,0492101	0,009334753	0,037747407	ag_LACat|PROMOTER
LRP1B	chr2:142888254-142888299	0,04392528	0,009556762	0,03513196	ag_LACat|INSIDE
LRPAP1	chr4:3533394-3533444	0,037901316	0,016473204	0,030107805	ag_LACat|INSIDE
LRRN1	chr3:3833485-3833544	0,04900549	0,013364397	0,029612208	ag_LACat|PROMOTER
LYPD1	chr2:133428908-133428953	0,024360929	0,013913301	0,029322169	ag_LACat|PROMOTER
MAP1S	chr19:17830393-17830437	0,040368643	0,012942676	0,029800395	ag_LACat|INSIDE
MARS	chr12:57881685-57881741	0,019400656	0,013283019	0,029737416	ag_LACat|DIVERGENT_PROMOTER
MATR3	chr5:138642650-138642709	0,032403044	0,049731288	0,049808271	ag_LACat|INSIDE
MBNL1	chr3:151985257-151985316	0,046867695	0,033793863	0,040716256	ag_LACat|PROMOTER
MBNL3	chrX:131546359-131546406	0,0469266	0,013040189	0,029918448	ag_LACat|INSIDE
MBP	chr18:74785056-74785115	0,03565538	0,007690392	0,059947992	ag_LACat|INSIDE
MC5R	chr18:13824274-13824333	0,028357966	0,048095733	0,049159462	ag_LACat|PROMOTER
MCF2	chrX:138728184-138728243	0,045189615	0,022737402	0,033663842	ag_LACat|PROMOTER
MCF2L	chr13:113718767-113718814	0,034120556	0,01642249	0,030185656	ag_LACat|INSIDE
MCM6	chr2:136633822-136633866	0,025837816	0,005197506	0,168139332	ag_LACat|INSIDE
MCM8	chr20:5931322-5931374	0,020710036	0,003675479	2,378035042	ag_LACat|INSIDE
MCPH1	chr8:6263874-6263925	0,021575488	0,012892195	0,029790179	ag_LACat|PROMOTER
MED14	chrX:40510712-40510771	0,035525657	0,008046689	0,056589212	ag_LACat|INSIDE
MEG3	chr14:101292569-101292613	0,033611666	0,015486024	0,030270264	ag_LACat|INSIDE
MEGF6	chr1:3453172-3453216	0,048335154	0,017132802	0,030706158	ag_LACat|INSIDE

MEIS2	chr15:37391492-37391549	0,034932047	0,026043324	0,035548588	ag_LACat|PROMOTER
MGLL	chr3:127541393-127541449	0,04429777	0,018972723	0,031801429	ag_LACat|INSIDE
MIAT	chr22:27053419-27053463	0,04377369	0,04485102	0,047184732	ag_LACat|PROMOTER
MID1	chrX:10549503-10549562	0,046186578	0,02175016	0,033426018	ag_LACat|PROMOTER
MID1IP1	chrX:38654470-38654529	0,0147698	0,03214058	0,039685029	ag_LACat|PROMOTER
MIER3	chr5:56242415-56242474	0,025700094	0,013362044	0,029708737	ag_LACat|INSIDE
MIR124-3	chr20:61807101-61807145	0,09744925	0,010735963	0,031573491	ag_LACat|PROMOTER
MIR1298	chrX:113941921-113941980	0,023395026	0,04916321	0,049623396	ag_LACat|PROMOTER
MIR130A	chr11:57405464-57405508	0,092344105	0,009698527	0,033736274	ag_LACat|PROMOTER
MIR1911	chrX:113991635-113991694	0,037414398	0,03453216	0,04099506	ag_LACat|PROMOTER
MIR3124	chr1:249120378-249120437	0,028469816	0,039534245	0,043949582	ag_LACat|DIVERGENT_PROMOTER
MIR448	chrX:114057148-114057206	0,04051406	0,024577519	0,034948692	ag_LACat|PROMOTER
MIR505	chrX:139006244-139006303	0,09687852	0,007738547	0,05960524	ag_LACat|DOWNSTREAM
MIR514-2	chrX:146369750-146369803	0,041718166	0,016685309	0,030154734	ag_LACat|PROMOTER
MITF	chr3:69788791-69788841	0,029756183	0,020413594	0,033018988	ag_LACat|INSIDE
MLL5	chr7:104656401-104656460	0,049398355	0,011607285	0,030160295	ag_LACat|INSIDE
MMGT1	chrX:135055920-135055964	0,057959806	0,009290425	0,037804434	ag_LACat|INSIDE
MORF4L2	chrX:102935201-102935260	0,019452294	0,041102882	0,044695067	ag_LACat|INSIDE
MOSPD1	chrX:134032257-134032316	0,035234194	0,022877188	0,033716493	ag_LACat|INSIDE
MPDZ	chr9:13176743-13176802	0,015941989	0,008879929	0,040459958	ag_LACat|INSIDE
MSL3	chrX:11770194-11770253	0,025006399	0,008998328	0,039337285	ag_LACat|PROMOTER
MSMP	chr9:35756835-35756884	0,033933375	0,009604462	0,034331972	ag_LACat|PROMOTER
MUM1L1	chrX:105406708-105406767	0,039459445	0,014611849	0,029729139	ag_LACat|PROMOTER
MYC	chr8:128749447-128749506	0,02676875	0,034195095	0,040819606	ag_LACat|INSIDE
MYCN	chr2:16080627-16080677	0,03361255	0,009346967	0,037330171	ag_LACat|PROMOTER
MYNN	chr3:169489468-169489527	0,050414976	0,00625672	0,103797374	ag_LACat|DIVERGENT_PROMOTER
MYO10	chr5:16935471-16935520	0,03732752	0,015154412	0,029984418	ag_LACat|INSIDE
NADK	chr1:1688667-1688713	0,028715316	0,026034225	0,035686745	ag_LACat|INSIDE
NAP1L3	chrX:92922004-92922063	0,027760044	0,025278047	0,035247622	ag_LACat|DOWNSTREAM
NCRNA00183	chrX:73163782-73163841	0,04569644	0,009718342	0,033445571	ag_LACat|PROMOTER
NDP	chrX:43821532-43821591	0,047868624	0,033979423	0,040787916	ag_LACat|INSIDE
NFATC2	chr20:50158678-50158722	0,03494818	0,02530426	0,035132739	ag_LACat|INSIDE
NFIA	chr1:61542598-61542642	0,04926532	0,014397588	0,029572189	ag_LACat|PROMOTER
NFKB2	chr10:104153957-104154001	0,023731668	0,009999988	0,032189016	ag_LACat|PROMOTER
NGLY1	chr3:25831465-25831509	0,03799917	0,0187712	0,031545367	ag_LACat|PROMOTER
NHS	chrX:17652475-17652532	0,04095015	0,019909281	0,032776857	ag_LACat|PROMOTER
NINJ1	chr9:95896459-95896503	0,03615347	0,045297984	0,047500479	ag_LACat|INSIDE
NIPAL2	chr8:99306732-99306785	0,032720294	0,009674487	0,034018441	ag_LACat|PROMOTER
NKX2-2	chr20:21494309-21494361	0,0389405	0,008284113	0,051045915	ag_LACat|INSIDE
NKX2-8	chr14:37051699-37051754	0,019798838	0,048538342	0,049533608	ag_LACat|INSIDE
NLRP12	chr19:54313894-54313946	0,05854107	0,007178996	0,066354433	ag_LACat|PROMOTER
NNT	chr5:43602921-43602971	0,026839348	0,016325383	0,030439547	ag_LACat|PROMOTER
NOL4	chr18:31803554-31803613	0,048781954	0,029888254	0,038141421	ag_LACat|PROMOTER
NOL8	chr9:95063683-95063742	0,03981939	0,037158445	0,042551352	ag_LACat|INSIDE
NOP58	chr2:203130420-203130465	0,048847318	0,0337381	0,040724908	ag_LACat|PROMOTER
NPPC	chr2:232798987-232799031	0,020888897	0,028446898	0,037257375	ag_LACat|PROMOTER
NPR3	chr5:32708234-32708293	0,029073762	0,009478378	0,036073592	ag_LACat|PROMOTER
NPY1R	chr4:164253318-164253369	0,029034877	0,004923357	0,227529427	ag_LACat|INSIDE
NR0B1	chrX:30327202-30327246	0,02268691	0,017183287	0,030711565	ag_LACat|INSIDE
NR2E1	chr6:108479088-108479142	0,03625607	0,015769107	0,030364917	ag_LACat|PROMOTER
NR2F2	chr15:96865146-96865204	0,04093612	0,009249177	0,037874794	ag_LACat|PROMOTER
NR4A3	chr9:102580291-102580350	0,03568325	0,049598325	0,049752118	ag_LACat|PROMOTER
NTRK3	chr15:88798600-88798657	0,037535805	0,037287164	0,042623313	ag_LACat|INSIDE
NUFIP1	chr13:45563603-45563649	0,047340956	0,01364489	0,029624979	ag_LACat|DIVERGENT_PROMOTER
NUP62CL	chrX:106447167-106447226	0,043812644	0,008301584	0,049732637	ag_LACat|INSIDE
ODC1	chr2:10589346-10589405	0,035439536	0,005490134	0,154439856	ag_LACat|PROMOTER
OGFOD1	chr16:56485368-56485414	0,04980368	0,015061465	0,029891926	ag_LACat|DIVERGENT_PROMOTER
OGT	chrX:70752954-70753006	0,013132322	0,008203622	0,052551915	ag_LACat|INSIDE
OLFM1	chr9:137978338-137978383	0,032804247	0,006383817	0,093871127	ag_LACat|INSIDE
OMA1	chr1:59011669-59011725	0,03739124	0,010098334	0,032185331	ag_LACat|INSIDE
OR51A4	chr11:4968503-4968562	0,03343527	0,006856756	0,072726579	ag_LACat|PROMOTER
OR52L1	chr11:6014083-6014142	0,04477865	0,013700052	0,029546445	ag_LACat|PROMOTER
OR52N4	chr11:5772188-5772247	0,040020764	0,036849197	0,042347123	ag_LACat|PROMOTER
OSBPL5	chr11:3157196-3157240	0,032765612	0,033107284	0,040415873	ag_LACat|INSIDE
OSTF1	chr9:77703784-77703833	0,030240646	0,034395095	0,040982738	ag_LACat|INSIDE
OTP	chr5:76941234-76941293	0,03326653	0,017513162	0,030874702	ag_LACat|PROMOTER
OXSM	chr3:25831668-25831717	0,029146226	0,040792126	0,04465737	ag_LACat|INSIDE
PABPC5	chrX:90683336-90683395	0,018510994	0,039893772	0,044197381	ag_LACat|PROMOTER
PAK3	chrX:110340250-110340294	0,09942334	0,013171727	0,029797578	ag_LACat|INSIDE

PAK6	chr15:40509327-40509386	0,029661845	0,019304285	0,032190393	ag_LACat|PROMOTER
PAPPA2	chr1:176521858-176521907	0,031948045	0,02536128	0,035136506	ag_LACat|INSIDE
PARD3	chr10:34667812-34667871	0,032793663	0,027902078	0,037145359	ag_LACat|INSIDE
PAX2	chr10:102503830-102503874	0,03624974	0,04006079	0,044306549	ag_LACat|PROMOTER
PCBD2	chr5:134241167-134241226	0,034578416	0,02973166	0,03809185	ag_LACat|INSIDE
PCDH11X	chrY:4868597-4868656	0,03969371	0,029888585	0,038066761	ag_LACat|INSIDE
PCDH11Y	chrY:4868786-4868831	0,026135884	0,006206465	0,108529258	ag_LACat|INSIDE
PCDH17	chr13:58206273-58206332	0,032372884	0,021747086	0,033500868	ag_LACat|INSIDE
PCDH19	chrX:99664127-99664171	0,088905185	0,008493331	0,046569366	ag_LACat|INSIDE
PCDH7	chr4:30716667-30716726	0,03411404	0,008779135	0,042074817	ag_LACat|PROMOTER
PCMTD1	chr8:52809989-52810048	0,048896402	0,016061798	0,030564657	ag_LACat|INSIDE
PCYT1B	chrX:24644519-24644578	0,027112935	0,008639041	0,042995843	ag_LACat|INSIDE
PDE12	chr3:57541818-57541877	0,030095432	0,026088012	0,035534618	ag_LACat|PROMOTER
PDE4D	chr5:58514339-58514398	0,0737928	0,009104155	0,039009194	ag_LACat|INSIDE
PDGFRB	chr5:149500818-149500864	0,031463906	0,00800172	0,057523476	ag_LACat|INSIDE
PDZRN3	chr3:73674097-73674141	0,06917291	0,006015661	0,125552667	ag_LACat|PROMOTER
PENK	chr8:57359837-57359883	0,05012536	0,006619286	0,079308856	ag_LACat|PROMOTER
PFN1	chr17:4850584-4850635	0,06814562	0,008987511	0,039557276	ag_LACat|INSIDE
PHACTR4	chr1:28696708-28696757	0,03895352	0,008155284	0,053841515	ag_LACat|INSIDE
PHF12	chr17:27277255-27277299	0,030011617	0,011837066	0,029916335	ag_LACat|INSIDE
PHF15	chr5:133861317-133861376	0,022329517	0,011275545	0,030652427	ag_LACat|PROMOTER
PHF17	chr4:129733820-129733869	0,024187244	0,035040595	0,041145671	ag_LACat|INSIDE
PHKA1	chrX:71934246-71934302	0,023221966	0,0241026	0,034731363	ag_LACat|PROMOTER
PHKA2	chrX:19001588-19001642	0,045027867	0,01010774	0,032057391	ag_LACat|INSIDE
PIK3R5	chr17:8819557-8819609	0,02033657	0,029328171	0,037799455	ag_LACat|PROMOTER
PIM3	chr22:50352318-50352366	0,047818005	0,010796173	0,031183589	ag_LACat|PROMOTER
PITX2	chr4:111562521-111562566	0,031303022	0,021131516	0,033184687	ag_LACat|INSIDE
PJA1	chrX:68385618-68385677	0,048279222	0,026516207	0,035891184	ag_LACat|PROMOTER
PLS3	chrX:114794115-114794174	0,03125043	0,023058947	0,033753707	ag_LACat|PROMOTER
PNMAL2	chr19:46997566-46997610	0,02772622	0,02468036	0,034941341	ag_LACat|INSIDE
POF1B	chrX:84633109-84633168	0,063773155	0,009975625	0,032271147	ag_LACat|INSIDE
POLE3	chr9:116172886-116172931	0,0839219	0,007890985	0,057364801	ag_LACat|INSIDE
POLR2A	chr17:7387649-7387693	0,027669104	0,017959984	0,030986959	ag_LACat|DIVERGENT_PROMOTER
PON2	chr7:95064481-95064540	0,02332004	0,02449736	0,034988503	ag_LACat|PROMOTER
POU3F2	chr6:99282338-99282382	0,036061913	0,027725264	0,036986074	ag_LACat|PROMOTER
POU3F4	chrX:82764734-82764793	0,03523648	0,025016073	0,035109326	ag_LACat|INSIDE
PPA1	chr10:71992316-71992373	0,024079192	0,016563216	0,030102249	ag_LACat|INSIDE
PPA2	chr4:106394596-106394640	0,031321257	0,010739302	0,0314404	ag_LACat|INSIDE
PPFIA2	chr12:82152407-82152461	0,025281353	0,009120201	0,038567124	ag_LACat|INSIDE
PPIC	chr5:122372482-122372526	0,04825134	0,009417946	0,036270302	ag_LACat|PROMOTER
PPID	chr4:159644552-159644596	0,042505257	0,009798744	0,032848639	ag_LACat|PROMOTER
PPM1L	chr3:160472735-160472780	0,033354186	0,004123451	0,533574521	ag_LACat|PROMOTER
PPP1R1B	chr17:37774591-37774636	0,05106427	0,00881964	0,041052569	ag_LACat|PROMOTER
PQLC3	chr2:11294752-11294811	0,048761357	0,014138164	0,029224895	ag_LACat|PROMOTER
PRAME	chr22:22899992-22900036	0,04281526	0,014119091	0,029279012	ag_LACat|INSIDE
PRDM12	chr9:133535753-133535802	0,048788503	0,046377197	0,048241232	ag_LACat|PROMOTER
PRDM16	chr1:2987684-2987728	0,022801146	0,020735804	0,033456522	ag_LACat|INSIDE
PRELID2	chr5:145203773-145203832	0,03641192	0,04871617	0,049558745	ag_LACat|INSIDE
PRICKLE3	chrX:49040936-49040980	0,034829427	0,009603377	0,034518803	ag_LACat|INSIDE
PRKACB	chr1:84606731-84606790	0,065794885	0,008398368	0,048086231	ag_LACat|PROMOTER
PRKCD	chr3:53203823-53203869	0,03981037	0,00855088	0,043562357	ag_LACat|INSIDE
PRR16	chr5:119953785-119953844	0,048664037	0,031981725	0,03956439	ag_LACat|INSIDE
PRR7	chr5:176881527-176881571	0,03598398	0,012821981	0,029841085	ag_LACat|INSIDE
PRRG1	chrX:37200375-37200434	0,054313764	0,007861916	0,057802951	ag_LACat|PROMOTER
PSIMCT-1	chr20:30135001-30135053	0,020205552	0,008617814	0,043560357	ag_LACat|PROMOTER
PSMD10	chrX:107332245-107332304	0,042617626	0,014961666	0,029877153	ag_LACat|INSIDE
PTDSS1	chr8:97273985-97274032	0,021295547	0,010878008	0,031141908	ag_LACat|DIVERGENT_PROMOTER
PTPN13	chr4:87515616-87515660	0,047933042	0,027928153	0,037103727	ag_LACat|INSIDE
PTPRG	chr3:61971035-61971094	0,029731853	0,02612091	0,035430249	ag_LACat|INSIDE
PUS7L	chr12:44147804-44147863	0,026250456	0,006803442	0,073363783	ag_LACat|INSIDE
RAB23	chr6:57086950-57087009	0,03446622	0,013840557	0,029456712	ag_LACat|PROMOTER
RAB33A	chrX:129304032-129304091	0,04046954	0,042121556	0,045421078	ag_LACat|DIVERGENT_PROMOTER
RAB5B	chr12:56379023-56379082	0,040219594	0,047325704	0,048680017	ag_LACat|INSIDE
RAB6B	chr3:133614906-133614965	0,024249936	0,006707313	0,076133886	ag_LACat|PROMOTER
RAP2B	chr3:152880909-152880953	0,025658054	0,006284389	0,099170724	ag_LACat|INSIDE
RBBP5	chr1:205091243-205091302	0,048449002	0,011790915	0,030272706	ag_LACat|PROMOTER
RBBP7	chrX:16888926-16888976	0,029136606	0,018143263	0,031219923	ag_LACat|PROMOTER
RBM15	chr1:110881973-110882032	0,020155057	0,017685309	0,030925392	ag_LACat|INSIDE
RCL1	chr9:4793459-4793509	0,020090308	0,010973362	0,031003342	ag_LACat|INSIDE

RELN	chr7:103629211-103629270	0,026311146	0,03885031	0,04363915	ag_LACat|INSIDE
RET	chr10:43573183-43573227	0,047607586	0,024568722	0,035013135	ag_LACat|INSIDE
RFX7	chr15:56537022-56537080	0,025852697	0,004846196	0,261290734	ag_LACat|PROMOTER
RHOV	chr15:41165228-41165280	0,048100132	0,032378793	0,039676286	ag_LACat|INSIDE
RNF128	chrX:105938312-105938371	0,030856015	0,008245056	0,052299522	ag_LACat|INSIDE
RNF141	chr11:10561173-10561232	0,04112682	0,04889986	0,04958967	ag_LACat|INSIDE
RPL10	chrX:153629011-153629062	0,046325356	0,018454267	0,031670851	ag_LACat|INSIDE
RPL39	chrX:118925837-118925886	0,037560176	0,037726786	0,042973997	ag_LACat|PROMOTER
RPRM	chr2:154334468-154334513	0,037963107	0,024919096	0,03504925	ag_LACat|INSIDE
RPS4Y2	chrY:22917984-22918032	0,04422896	0,025598066	0,035388779	ag_LACat|INSIDE
RPS6KA3	chrX:20168758-20168817	0,039245233	0,008150289	0,054363268	ag_LACat|INSIDE
RPSA	chr3:39448150-39448198	0,032910086	0,026096376	0,035471335	ag_LACat|PROMOTER
RREB1	chr6:7108395-7108439	0,090027176	0,00826639	0,05192577	ag_LACat|INSIDE
RTN4	chr2:55252799-55252858	0,032781992	0,007111824	0,067666911	ag_LACat|INSIDE
RUNDC3B	chr7:87254994-87255053	0,032864895	0,005130726	0,184421096	ag_LACat|PROMOTER
RUNX1	chr21:36260978-36261027	0,015215702	0,017425526	0,03080414	ag_LACat|PROMOTER
RXRA	chr9:137212016-137212067	0,026532928	0,02227228	0,033512012	ag_LACat|PROMOTER
S100A6	chr1:153508705-153508749	0,04529887	0,018573247	0,031457829	ag_LACat|PROMOTER
SACM1L	chr3:45731470-45731529	0,027512234	0,036421794	0,04208018	ag_LACat|INSIDE
SAFB	chr19:5623028-5623072	0,041069675	0,006168202	0,11402362	ag_LACat|INSIDE
SAP30L	chr5:153825479-153825523	0,037789054	0,014401039	0,029485672	ag_LACat|DIVERGENT_PROMOTER
SCAP	chr3:47491917-47491976	0,026993502	0,029840076	0,038155196	ag_LACat|INSIDE
SCML1	chrX:17762846-17762905	0,019517	0,040552955	0,044546285	ag_LACat|INSIDE
SEC31A	chr4:83800153-83800212	0,030882169	0,013681115	0,029604286	ag_LACat|INSIDE
SELK	chr3:53926492-53926547	0,022493204	0,007626611	0,061680216	ag_LACat|PROMOTER
SELM	chr22:31502835-31502879	0,041154936	0,009834589	0,032464179	ag_LACat|INSIDE
SEMA3B	chr3:50297190-50297241	0,029614234	0,034864526	0,041238297	ag_LACat|PROMOTER
SEPT7	chr7:35840384-35840439	0,025873698	0,048992194	0,049605555	ag_LACat|PROMOTER
SETD1A	chr16:30968582-30968626	0,027330268	0,007177766	0,067304555	ag_LACat|PROMOTER
SETD2	chr3:47164182-47164241	0,039610215	0,02106436	0,033240588	ag_LACat|INSIDE
SFMBT1	chr3:52939766-52939825	0,026765142	0,04301526	0,046154019	ag_LACat|INSIDE
SGCZ	chr8:14417279-14417338	0,046842854	0,009107527	0,038766908	ag_LACat|INSIDE
SH3BGRL	chrX:80452708-80452767	0,038318284	0,021834325	0,033317944	ag_LACat|PROMOTER
SH3KBP1	chrX:19664725-19664784	0,034464132	0,011174493	0,030635157	ag_LACat|INSIDE
SH3YL1	chr2:260080-260139	0,025236847	0,008817317	0,041640906	ag_LACat|INSIDE
SHOX	chrX:591633-591683	0,06588942	0,006589475	0,080441322	ag_LACat|INSIDE
SHOX	chrY:541633-541683	0,06588942	0,006589475	0,081988271	ag_LACat|INSIDE
SHROOM2	chrX:9753501-9753546	0,05495067	0,004023687	0,650831372	ag_LACat|PROMOTER
SIRPB1	chr20:1569895-1569954	0,021012329	0,009488036	0,035899177	ag_LACat|INSIDE
SIX1	chr14:61119818-61119877	0,045835834	0,022366988	0,033498707	ag_LACat|PROMOTER
SKOR1	chr15:68112766-68112813	0,032364845	0,013834787	0,029541608	ag_LACat|PROMOTER
SLC12A6	chr15:34629948-34629992	0,03522846	0,026656935	0,03600634	ag_LACat|PROMOTER
SLC16A2	chrX:73642166-73642221	0,032072842	0,009496032	0,035720539	ag_LACat|INSIDE
SLC26A1	chr4:990811-990870	0,024144456	0,012768897	0,029932885	ag_LACat|PROMOTER
SLC26A6	chr3:48671666-48671710	0,046465673	0,03573139	0,041579513	ag_LACat|PROMOTER
SLC2A2	chr3:170746108-170746160	0,033896733	0,010054018	0,032202721	ag_LACat|PROMOTER
SLC35A2	chrX:48762501-48762545	0,032073896	0,028203981	0,037240767	ag_LACat|INSIDE
SLC38A1	chr12:46661084-46661129	0,03165353	0,047828943	0,048964124	ag_LACat|INSIDE
SLC45A4	chr8:142236996-142237055	0,0302609	0,01708846	0,03071176	ag_LACat|INSIDE
SLC46A3	chr13:29293211-29293255	0,07172807	0,008954958	0,039683958	ag_LACat|PROMOTER
SLC7A2	chr8:17355659-17355718	0,037993126	0,011733005	0,030365017	ag_LACat|INSIDE
SLCO4A1	chr20:61267610-61267654	0,03450949	0,010795541	0,031321592	ag_LACat|PROMOTER
SLFN5	chr17:33569000-33569059	0,03866478	0,017764777	0,030897341	ag_LACat|PROMOTER
SLIT2	chr4:20256311-20256355	0,02733586	0,010284121	0,031836489	ag_LACat|INSIDE
SLITRK2	chrX:144897042-144897101	0,036396362	0,040806826	0,044598001	ag_LACat|PROMOTER
SMARCA1	chrX:128658230-128658289	0,055820234	0,008735727	0,042179219	ag_LACat|PROMOTER
SMC1A	chrX:53449475-53449531	0,038034853	0,02826109	0,037240174	ag_LACat|INSIDE
SNAI2	chr8:49834011-49834070	0,020266376	0,009748473	0,033196116	ag_LACat|PROMOTER
SNORA84	chr9:95055312-95055371	0,02910901	0,010753395	0,031339849	ag_LACat|PROMOTER
SNORD37	chr19:3982515-3982572	0,023162028	0,013882035	0,02944812	ag_LACat|INSIDE
SNORD60	chr16:2205034-2205089	0,021538943	0,020879999	0,033439008	ag_LACat|INSIDE
SNTG1	chr8:51307554-51307613	0,044166405	0,01849577	0,031574573	ag_LACat|INSIDE
SNX18	chr5:53813175-53813234	0,046675447	0,010630048	0,031548812	ag_LACat|PROMOTER
SOLH	chr16:602048-602092	0,046895627	0,006194011	0,111320142	ag_LACat|INSIDE
SORBS2	chr4:186733259-186733318	0,083244964	0,011325575	0,03065961	ag_LACat|PROMOTER
SOX1	chr13:112723236-112723289	0,022890668	0,020066217	0,032951377	ag_LACat|INSIDE
SOX3	chrX:139587188-139587232	0,039909057	0,016356293	0,030409545	ag_LACat|INSIDE
SP9	chr2:175196058-175196117	0,0401673	0,03228084	0,039631316	ag_LACat|PROMOTER
SPDEF	chr6:34514613-34514659	0,024504017	0,008721328	0,04242631	ag_LACat|INSIDE

SPON1	chr11:13985014-13985058	0,04899628	0,021269254	0,033239631	ag_LACat|INSIDE
SPRY2	chr13:80914788-80914847	0,024778603	0,025901053	0,035579578	ag_LACat|INSIDE
SPTB	chr14:65236182-65236228	0,028868232	0,020748697	0,033394047	ag_LACat|INSIDE
SRPX2	chrX:99901765-99901824	0,04528841	0,013494631	0,029596699	ag_LACat|INSIDE
SRY	chrY:2653984-2654043	0,035140872	0,012036664	0,029387629	ag_LACat|DOWNSTREAM
SSPN	chr12:26348882-26348932	0,018152997	0,011540667	0,030230006	ag_LACat|INSIDE
ST6GAL1	chr3:186650286-186650345	0,042233117	0,009729988	0,033308477	ag_LACat|INSIDE
ST8SIA2	chr15:92936183-92936242	0,035175394	0,016224777	0,030515787	ag_LACat|PROMOTER
SUB1	chr5:32585458-32585517	0,036677223	0,037852142	0,043041012	ag_LACat|PROMOTER
SUGT1	chr13:53226426-53226473	0,06802917	0,006316404	0,0973027	ag_LACat|PROMOTER
SUPT6H	chr17:26989114-26989158	0,03570167	0,006992758	0,068550222	ag_LACat|DIVERGENT_PROMOTER
SV2B	chr15:91643708-91643761	0,042937387	0,012708928	0,030119693	ag_LACat|INSIDE
SV2C	chr5:75380461-75380505	0,04377516	0,006574479	0,085073758	ag_LACat|INSIDE
TAB3	chrX:30874802-30874861	0,02710094	0,007294133	0,063774379	ag_LACat|INSIDE
TAF10	chr11:6633552-6633596	0,0451474	0,016474515	0,030025384	ag_LACat|PROMOTER
tAKR	chr10:4931549-4931608	0,022338439	0,023955239	0,034673467	ag_LACat|INSIDE
TATDN2	chr3:10289991-10290035	0,032478977	0,021779375	0,033391601	ag_LACat|PROMOTER
TBC1D16	chr17:78009944-78009990	0,016065063	0,01567689	0,030368107	ag_LACat|DIVERGENT_PROMOTER
TBCE	chr1:235531039-235531095	0,04211332	0,015498073	0,03020257	ag_LACat|INSIDE
TBRG1	chr11:124492738-124492782	0,029868077	0,047356963	0,048634849	ag_LACat|INSIDE
TBX5	chr12:114847509-114847566	0,047914334	0,010836107	0,031159828	ag_LACat|PROMOTER
TCEAL4	chrX:102834791-102834850	0,026104428	0,034923688	0,041232894	ag_LACat|PROMOTER
TCEAL8	chrX:102510299-102510353	0,028244384	0,040130995	0,044308454	ag_LACat|PROMOTER
TCF12	chr15:57211493-57211541	0,07186446	0,011195312	0,030562729	ag_LACat|INSIDE
TCF7L2	chr10:114710993-114711039	0,026377382	0,011435253	0,030572763	ag_LACat|INSIDE
TDRD3	chr13:61003629-61003688	0,03284982	0,011757696	0,030307687	ag_LACat|INSIDE
TEAD1	chr11:12694947-12695006	0,024104452	0,014077217	0,029475597	ag_LACat|PROMOTER
TES	chr7:115850285-115850340	0,029914148	0,014615972	0,029644307	ag_LACat|PROMOTER
TEX2	chr17:62340079-62340132	0,026047895	0,011017509	0,030725553	ag_LACat|INSIDE
TGIF1	chr18:3447873-3447926	0,029121622	0,027996551	0,037118378	ag_LACat|PROMOTER
THAP2	chr12:72070884-72070943	0,044565074	0,04394927	0,046615045	ag_LACat|INSIDE
THAP2	chr12:72057290-72057339	0,05650818	0,008290151	0,050601205	ag_LACat|PROMOTER
THNSL1	chr10:25305261-25305320	0,020705601	0,006620149	0,077877022	ag_LACat|DIVERGENT_PROMOTER
THOC2	chrX:122835780-122835835	0,0833315	0,010696397	0,031600771	ag_LACat|INSIDE
TKTL1	chrX:153523954-153524010	0,04798094	0,009695594	0,033908375	ag_LACat|DIVERGENT_PROMOTER
TLL2	chr10:98272702-98272746	0,025654515	0,02382386	0,034638286	ag_LACat|INSIDE
TLX1	chr10:102894072-102894116	0,04618974	0,017405763	0,030853503	ag_LACat|INSIDE
TM2D3	chr15:102192807-102192851	0,021125276	0,02912296	0,037760632	ag_LACat|PROMOTER
TM7SF3	chr12:27166746-27166805	0,047967725	0,030228391	0,038198768	ag_LACat|INSIDE
TMEM91	chr19:41863490-41863541	0,041253652	0,011052349	0,030690428	ag_LACat|PROMOTER
TMPRSS2	chr21:42879497-42879543	0,04779052	0,021887098	0,033241672	ag_LACat|INSIDE
TMSB15B	chrX:103217263-103217307	0,037398778	0,03067636	0,038389951	ag_LACat|INSIDE
TNIK	chr3:171178447-171178499	0,04257725	0,020921197	0,033339937	ag_LACat|PROMOTER
TNNT3	chr11:1959318-1959376	0,04347247	0,014082586	0,02939172	ag_LACat|INSIDE
TNRC18	chr7:5458859-5458908	0,03339152	0,0385332	0,043509564	ag_LACat|INSIDE
TNS3	chr7:47626581-47626640	0,0486528	0,043460194	0,04640057	ag_LACat|PROMOTER
TOP3B	chr22:22313127-22313182	0,025463402	0,014050489	0,029515151	ag_LACat|INSIDE
TOR1AIP1	chr1:179852092-179852136	0,045264427	0,008436222	0,04787926	ag_LACat|INSIDE
TRAF7	chr16:2205475-2205519	0,029541261	0,010530296	0,031688844	ag_LACat|DIVERGENT_PROMOTER
TRIM36	chr5:114515458-114515508	0,022980317	0,015588316	0,030287209	ag_LACat|INSIDE
TRIM67	chr1:231297115-231297163	0,030149156	0,013461391	0,029624217	ag_LACat|PROMOTER
TRIM71	chr3:32858491-32858536	0,07249726	0,007316725	0,06311895	ag_LACat|PROMOTER
TSC22D3	chrX:107018442-107018492	0,027925136	0,013274898	0,029822427	ag_LACat|INSIDE
TSLP	chr5:110408792-110408841	0,022934582	0,01343272	0,029662013	ag_LACat|PROMOTER
TSPYL5	chr8:98290171-98290215	0,035407804	0,025751332	0,035449174	ag_LACat|PROMOTER
TTLL12	chr22:43581819-43581863	0,028496293	0,016387604	0,030293657	ag_LACat|INSIDE
TUBB3	chr16:89988649-89988696	0,036438175	0,020279106	0,033049324	ag_LACat|PROMOTER
TXNRD1	chr12:104682986-104683045	0,02497805	0,011156718	0,030716581	ag_LACat|INSIDE
TYW3	chr1:75196487-75196546	0,040130306	0,009938093	0,032311287	ag_LACat|PROMOTER
UACA	chr15:71054321-71054380	0,03822622	0,024284586	0,034761343	ag_LACat|INSIDE
UBE2A	chrX:118712860-118712919	0,021932403	0,028821157	0,037444355	ag_LACat|PROMOTER
UBLCP1	chr5:158695743-158695802	0,048134066	0,021843478	0,033253483	ag_LACat|INSIDE
UGT3A2	chr5:36066561-36066615	0,042989463	0,032264724	0,039686837	ag_LACat|INSIDE
UNC80	chr2:210628996-210629055	0,035531167	0,039730016	0,044091459	ag_LACat|PROMOTER
UQCR11	chr19:1604945-1605003	0,0188006	0,035026796	0,041204249	ag_LACat|INSIDE
USP9Y	chrY:14804644-14804703	0,084846474	0,009640345	0,034270897	ag_LACat|PROMOTER
UTP3	chr4:71554231-71554275	0,04444203	0,014716647	0,029478856	ag_LACat|INSIDE
VCAN	chr5:82760846-82760905	0,02787024	0,016368812	0,03034562	ag_LACat|PROMOTER
VDAC3	chr8:42249091-42249135	0,029475998	0,045983423	0,047985927	ag_LACat|PROMOTER

VENTXP1	chrX:26567427-26567486	0,031564236	0,043464284	0,046328487	ag_LACat|PROMOTER
VSX2	chr14:74707877-74707936	0,03077397	0,008440001	0,04748418	ag_LACat|INSIDE
WDR44	chrX:117479931-117479975	0,042114615	0,012410741	0,029961752	ag_LACat|PROMOTER
WDR45L	chr17:80605538-80605582	0,044894684	0,018458806	0,031594835	ag_LACat|INSIDE
WNT7B	chr22:46366897-46366956	0,02998653	0,035795595	0,041579443	ag_LACat|INSIDE
XAF1	chr17:6659145-6659189	0,040385637	0,024796298	0,035028831	ag_LACat|INSIDE
XIAP	chrX:123018629-123018688	0,02928918	0,00807319	0,055567595	ag_LACat|INSIDE
XKR8	chr1:28286116-28286170	0,02443053	0,012194552	0,029661184	ag_LACat|PROMOTER
YEATS4	chr12:69753886-69753930	0,025163136	0,022194859	0,033473365	ag_LACat|INSIDE
YY1	chr14:100701286-100701345	0,0477498	0,043399274	0,046412116	ag_LACat|PROMOTER
ZBTB33	chrX:119387101-119387160	0,033793073	0,046930864	0,048660688	ag_LACat|INSIDE
ZC3HAV1	chr7:138793518-138793565	0,044172805	0,011817814	0,030102857	ag_LACat|INSIDE
ZC4H2	chrX:64195826-64195878	0,025375472	0,011926467	0,029340016	ag_LACat|INSIDE
ZDHHC8P1	chr22:23744727-23744774	0,032629296	0,04129398	0,044752437	ag_LACat|INSIDE
ZFHX3	chr16:73082098-73082148	0,043869838	0,011886379	0,02980809	ag_LACat|INSIDE
ZFP161	chr18:5296117-5296161	0,033350457	0,025713217	0,035472178	ag_LACat|PROMOTER
ZFP91	chr11:58346129-58346178	0,041238632	0,009584428	0,03483778	ag_LACat|DIVERGENT_PROMOTER
ZFX	chrX:24165394-24165453	0,02962841	0,013131436	0,03002134	ag_LACat|PROMOTER
ZFY	chrY:2800633-2800692	0,023214763	0,042951163	0,046238606	ag_LACat|PROMOTER
ZMAT1	chrX:101150202-101150261	0,018259967	0,009346876	0,037561669	ag_LACat|PROMOTER
ZNF35	chr3:44690131-44690175	0,039751988	0,04381779	0,046628471	ag_LACat|PROMOTER
ZNF521	chr18:22931432-22931491	0,04149751	0,012027297	0,029475989	ag_LACat|INSIDE
ZNF628	chr19:55988366-55988425	0,022180377	0,009527527	0,03563185	ag_LACat|INSIDE
ZNF697	chr1:120173954-120174003	0,01886032	0,012793585	0,029882489	ag_LACat|INSIDE
ZNF711	chrX:84499393-84499437	0,034435883	0,04437368	0,046988169	ag_LACat|INSIDE
ZNF740	chr12:53575303-53575357	0,040623404	0,011836874	0,030033167	ag_LACat|INSIDE
ZNF771	chr16:30411882-30411929	0,05342023	0,01027767	0,031969483	ag_LACat|PROMOTER
ZNF789	chr7:99070249-99070308	0,03112721	0,034642782	0,041051062	ag_LACat|DIVERGENT_PROMOTER

Supplementary Table S4

Primary Annotation	Name	P-value	P[Xbar]	FDR	Primary Annotation Type
ACAA2	chr18:47339567-47339617	0,09189961	0,010360939	0,046138556	ag_LACat|INSIDE
ACE2	chrX:15624677-15624736	0,046445977	0,03825596	0,042756661	ag_LACat|PROMOTER
ACTR1A	chr10:104262116-104262166	0,039695047	0,014555463	0,034002516	ag_LACat|INSIDE
ALCAM	chr3:105081027-105081086	0,04181391	0,011286816	0,043469494	ag_LACat|PROMOTER
AMELY	chrY:6741252-6741311	0,039029967	0,042921472	0,045305998	ag_LACat|INSIDE
ANO10	chr3:43662282-43662341	0,049730174	0,029638229	0,036409031	ag_LACat|INSIDE
ANXA2	chr15:60678646-60678705	0,042079087	0,016105972	0,032787157	ag_LACat|INSIDE
APLP2	chr11:129940699-129940749	0,04002799	0,041998263	0,044829607	ag_LACat|INSIDE
APOOL	chrX:84298951-84299010	0,038401417	0,015634935	0,032764386	ag_LACat|INSIDE
ARMCX1	chrX:100807734-100807791	0,025040837	0,010222474	0,047760739	ag_LACat|INSIDE
ARMCX4	chrX:100669536-100669595	0,027163275	0,012766846	0,036385511	ag_LACat|PROMOTER
ASPN	chr9:95243272-95243331	0,033466037	0,01174278	0,040321594	ag_LACat|INSIDE
ATF1	chr12:51156641-51156700	0,03184843	0,008201283	0,070829258	ag_LACat|PROMOTER
ATOH8	chr2:85981876-85981932	0,016445182	0,00853567	0,064017525	ag_LACat|INSIDE
ATP6V1A	chr3:113470917-113470976	0,039261844	0,010367967	0,045459548	ag_LACat|INSIDE
ATXN8OS	chr13:70672987-70673046	0,03456721	0,023311399	0,033218744	ag_LACat|PROMOTER
AZI2	chr3:28385590-28385649	0,026234536	0,008391487	0,06833068	ag_LACat|INSIDE
BAG6	chr6:31620215-31620261	0,033520196	0,04100649	0,044606296	ag_LACat|PROMOTER
BARX2	chr11:129244335-129244394	0,018223405	0,03362779	0,039440001	ag_LACat|PROMOTER
BCL11B	chr14:99735676-99735735	0,08645972	0,008702089	0,060490131	ag_LACat|INSIDE
BCL2L11	chr2:111880575-111880619	0,026017241	0,023086203	0,033230141	ag_LACat|INSIDE
BEX5	chrX:101408648-101408707	0,018297896	0,013145644	0,03534442	ag_LACat|DOWNSTREAM
BMX	chrX:15521372-15521431	0,02478177	0,011573859	0,041231873	ag_LACat|PROMOTER
BRIX1	chr5:34915872-34915920	0,033462025	0,011170321	0,044215854	ag_LACat|INSIDE
BRWD3	chrX:80066713-80066772	0,04382708	0,019430319	0,031643662	ag_LACat|PROMOTER
BZW2	chr7:16691509-16691568	0,042790473	0,025644422	0,033993769	ag_LACat|INSIDE
C12orf34	chr12:110174842-110174901	0,03000976	0,024039118	0,034085317	ag_LACat|INSIDE
C13orf15	chr13:42030869-42030928	0,025160104	0,04151091	0,044643809	ag_LACat|PROMOTER
C14orf37	chr14:58618929-58618973	0,031740554	0,013730655	0,035254383	ag_LACat|PROMOTER
C1orf159	chr1:1051804-1051848	0,034885593	0,015842412	0,032956842	ag_LACat|PROMOTER
C20orf177	chr20:58515630-58515680	0,02236737	0,025356105	0,033768645	ag_LACat|INSIDE
C3orf31	chr3:11888188-11888232	0,029523231	0,03812428	0,042777243	ag_LACat|INSIDE
C3orf78	chr3:52570282-52570336	0,034548443	0,015619036	0,03297352	ag_LACat|DIVERGENT_PROMOTER
C4orf33	chr4:130017140-130017199	0,040099725	0,006724293	0,273774786	ag_LACat|PROMOTER
C5orf13	chr5:111090578-111090637	0,039788753	0,009080603	0,058817542	ag_LACat|INSIDE
C7orf58	chr7:120628434-120628491	0,029385995	0,01225808	0,038817253	ag_LACat|PROMOTER
C8orf4	chr8:40012288-40012347	0,056211542	0,009209414	0,055844319	ag_LACat|INSIDE
C9orf150	chr9:12766420-12766479	0,046445966	0,013664292	0,035402938	ag_LACat|PROMOTER
CA5BP1	chrX:15688508-15688567	0,03761104	0,027218772	0,034943018	ag_LACat|DIVERGENT_PROMOTER
CALM1	chr14:90866182-90866241	0,023948248	0,040847674	0,044603782	ag_LACat|INSIDE
CAPZA1	chr1:113179120-113179179	0,03785954	0,021632334	0,032793698	ag_LACat|INSIDE
CCDC117	chr22:29169479-29169538	0,05258169	0,008066371	0,074158572	ag_LACat|INSIDE
CCDC36	chr3:49237200-49237244	0,030817479	0,0230415	0,03333415	ag_LACat|INSIDE
CCDC66	chr3:56591654-56591706	0,036462992	0,011994305	0,038408729	ag_LACat|INSIDE
CCND1	chr11:69453346-69453405	0,034823686	0,007797293	0,079365308	ag_LACat|PROMOTER
CCNI	chr4:77997218-77997262	0,04840225	0,029498396	0,036711978	ag_LACat|PROMOTER
CD200R1	chr3:112699788-112699847	0,03640954	0,007281885	0,129708577	ag_LACat|DIVERGENT_PROMOTER
CDK6	chr7:92467878-92467937	0,04810478	0,018025683	0,032108248	ag_LACat|PROMOTER
CDKL5	chrX:18526330-18526389	0,036067292	0,04684784	0,048027462	ag_LACat|INSIDE
CDX2	chr13:28544461-28544519	0,025901448	0,026855314	0,034789839	ag_LACat|PROMOTER
CEP57	chr11:95523383-95523436	0,029821435	0,006955711	0,180216141	ag_LACat|DIVERGENT_PROMOTER
CEP70	chr3:138313358-138313407	0,025816288	0,024491578	0,033883979	ag_LACat|PROMOTER
CHCHD2	chr7:56174339-56174383	0,06707639	0,010417267	0,044983653	ag_LACat|PROMOTER
CHD5	chr1:6240462-6240506	0,03720255	0,021702651	0,03272622	ag_LACat|PROMOTER
chr13:84944818-84944877	chr13:84944818-84944877	0,013909679	0,019164424	0,031755005	Unknown
chr13:84945079-84945138	chr13:84945079-84945138	0,02879232	0,006237587	0,355542431	Unknown
CNKSR2	chrX:21385025-21385084	0,029523408	0,012720883	0,036994405	ag_LACat|PROMOTER
CXCL12	chr10:44879552-44879596	0,0400737	0,018251656	0,031335674	ag_LACat|INSIDE
CXorf1	chrX:144908261-144908320	0,024393562	0,015315219	0,033575672	ag_LACat|PROMOTER
CXorf38	chrX:40508554-40508613	0,05261678	0,008810517	0,059785651	ag_LACat|PROMOTER
CXorf58	chrX:23926361-23926420	0,036598083	0,03963476	0,043782584	ag_LACat|INSIDE
CYHR1	chr8:145675310-145675368	0,02442018	0,021761341	0,032642012	ag_LACat|INSIDE
DCUN1D5	chr11:102962008-102962067	0,036500655	0,041084476	0,0445212	ag_LACat|INSIDE
DIAPH2	chrX:96140586-96140645	0,04456948	0,010761745	0,043815676	ag_LACat|INSIDE
DNAH14	chr1:225142208-225142267	0,046863735	0,00944737	0,051778855	ag_LACat|INSIDE
DNAJB4	chr1:78471490-78471548	0,036569685	0,030930256	0,037352216	ag_LACat|INSIDE
DNAJB6	chr7:157130341-157130388	0,032980297	0,012773306	0,036043487	ag_LACat|INSIDE
DNAJC25	chr9:114392264-114392323	0,02837767	0,012440574	0,03812434	ag_LACat|PROMOTER
DPP6	chr7:153583993-153584037	0,040819064	0,032225143	0,038267357	ag_LACat|PROMOTER

DTNA	chr18:32291677-32291736	0,027833417	0,024323499	0,034148755	ag_LACat|INSIDE
DTWD2	chr5:118324511-118324570	0,046565156	0,030901108	0,037475812	ag_LACat|PROMOTER
DUSP14	chr17:35848348-35848403	0,024633467	0,027083086	0,034926152	ag_LACat|PROMOTER
EDA2R	chrX:65837060-65837119	0,02605986	0,007690114	0,091320098	ag_LACat|INSIDE
EFNA5	chr5:107009315-107009363	0,034775432	0,013806422	0,034516055	ag_LACat|PROMOTER
EIF4B	chr12:53400209-53400254	0,024110967	0,015392219	0,033486889	ag_LACat|INSIDE
EIF5	chr14:103799974-103800018	0,044624787	0,013187517	0,035125628	ag_LACat|PROMOTER
ELK4	chr1:205600180-205600230	0,038900908	0,011296642	0,04292724	ag_LACat|INSIDE
EMCN	chr4:101447574-101447633	0,028105376	0,01018534	0,049200369	ag_LACat|PROMOTER
ENC1	chr5:73937926-73937985	0,02626888	0,013063366	0,035798647	ag_LACat|PROMOTER
ENOX2	chrX:129842512-129842571	0,033193737	0,025850832	0,034108737	ag_LACat|INSIDE
ENST00000367072:84	chr6:159291032-159291077	0,039890315	0,008811081	0,058399025	ag_LACat|INSIDE
ENST00000382528:-3438	chr7:1737370-1737429	0,041370917	0,018212102	0,03164908	ag_LACat|PROMOTER
ENST00000449698:-5113	chrX:36048770-36048829	0,02739369	0,007521347	0,107179195	ag_LACat|DIVERGENT_PROMOTER
ETS2	chr21:40176755-40176814	0,027392276	0,03349517	0,039446791	ag_LACat|PROMOTER
EYA1	chr8:72265338-72265393	0,042707007	0,009983199	0,050807352	ag_LACat|INSIDE
FAM134C	chr17:40761592-40761640	0,018279143	0,013867378	0,03436698	ag_LACat|PROMOTER
FAM199X	chrX:103410132-103410191	0,040517848	0,043758716	0,045850125	ag_LACat|DIVERGENT_PROMOTER
FCER1A	chr1:159257149-159257208	0,032557722	0,006932142	0,197566038	ag_LACat|PROMOTER
FGG	chr4:155533074-155533133	0,036440786	0,020472573	0,031710235	ag_LACat|INSIDE
FHAD1	chr1:15645289-15645348	0,021213677	0,04124153	0,044522106	ag_LACat|INSIDE
FKBP14	chr7:30060417-30060476	0,024332937	0,010535708	0,044157011	ag_LACat|INSIDE
FLNA	chrX:153598284-153598333	0,040472206	0,02062896	0,031779749	ag_LACat|INSIDE
FMR1	chrX:146994992-146995051	0,028076477	0,020000082	0,032022603	ag_LACat|INSIDE
FNDC3B	chr3:171757831-171757884	0,036292505	0,012317126	0,038575614	ag_LACat|PROMOTER
FOXN2	chr2:48541289-48541333	0,040961187	0,015444768	0,032848947	ag_LACat|PROMOTER
FOXP2	chr7:114068143-114068202	0,04838088	0,04225281	0,044765988	ag_LACat|INSIDE
FSTL5	chr4:163089574-163089633	0,021430688	0,015225348	0,033637397	ag_LACat|PROMOTER
FTMT	chr5:121187337-121187388	0,05595999	0,011748949	0,039862506	ag_LACat|PROMOTER
GABRA6	chr5:161106628-161106687	0,047773816	0,013139174	0,035663472	ag_LACat|PROMOTER
GC	chr4:72652240-72652296	0,07485718	0,01256865	0,03731318	ag_LACat|PROMOTER
GFI1	chr1:92951915-92951972	0,03563422	0,004897409	0,465253827	ag_LACat|PROMOTER
GPR34	chrX:41548410-41548469	0,040773373	0,017333185	0,032714952	ag_LACat|INSIDE
GPR64	chrX:19055024-19055074	0,02998653	0,011799161	0,039561893	ag_LACat|INSIDE
GPR82	chrX:41583077-41583136	0,04088929	0,022016399	0,032851695	ag_LACat|PROMOTER
GRIA3	chrX:122315714-122315773	0,028698878	0,017933654	0,03234868	ag_LACat|PROMOTER
GRPR	chrX:16177313-16177372	0,035454392	0,007761208	0,08507478	ag_LACat|DOWNSTREAM
GYG1	chr3:148708911-148708970	0,033622816	0,042227417	0,044906022	ag_LACat|PROMOTER
HACL1	chr3:15642856-15642901	0,045458954	0,027861033	0,035290642	ag_LACat|INSIDE
HECTD2	chr10:93168493-93168552	0,03596625	0,008427208	0,066715397	ag_LACat|PROMOTER
HES1	chr3:193853157-193853216	0,02261181	0,010317312	0,047426354	ag_LACat|PROMOTER
HFM1	chr1:91868905-91868964	0,036658842	0,02489163	0,033781498	ag_LACat|INSIDE
HHLA2	chr3:108020402-108020461	0,025522297	0,029830024	0,036331439	ag_LACat|PROMOTER
HINT2	chr9:35815195-35815248	0,044822436	0,016538696	0,032961737	ag_LACat|PROMOTER
HMGN5	chrX:80457414-80457473	0,03137343	0,019336266	0,03167147	ag_LACat|PROMOTER
HOXA5	chr7:27183397-27183447	0,044790976	0,019066047	0,031963667	ag_LACat|PROMOTER
HPCAL1	chr2:10444078-10444137	0,04778643	0,04768306	0,048534543	ag_LACat|INSIDE
hsa-mir-1321:-4814	chrX:85085942-85086001	0,06660097	0,009801891	0,050791617	ag_LACat|PROMOTER
HTATSF1	chrX:135578041-135578100	0,03780987	0,007043689	0,154419327	ag_LACat|PROMOTER
HUWE1	chrX:53656548-53656601	0,024196314	0,022501646	0,033227819	ag_LACat|INSIDE
ID2	chr2:8816725-8816769	0,09252049	0,011630022	0,040920448	ag_LACat|PROMOTER
IL13RA1	chrX:117859099-117859158	0,04444812	0,018239088	0,031503879	ag_LACat|PROMOTER
IL1RAPL2	chrX:103811717-103811762	0,043885823	0,011839664	0,038785106	ag_LACat|INSIDE
IMPA1	chr8:82598508-82598552	0,04841216	0,013995618	0,03409189	ag_LACat|INSIDE
ITGA1	chr5:52083791-52083841	0,036610246	0,007056766	0,143655587	ag_LACat|PROMOTER
KIAA0196	chr8:126095250-126095309	0,02075738	0,014584937	0,033794366	ag_LACat|INSIDE
KIAA0564	chr13:42412353-42412412	0,042190548	0,04764918	0,048673894	ag_LACat|INSIDE
KIAA0922	chr4:154387792-154387839	0,031017842	0,01741317	0,032649694	ag_LACat|INSIDE
KIF1B	chr1:10273134-10273193	0,04508527	0,02959754	0,036516445	ag_LACat|INSIDE
KIF26B	chr1:245319622-245319666	0,026004594	0,019708436	0,031733922	ag_LACat|INSIDE
KLRK1	chr12:10546267-10546326	0,026969805	0,011801686	0,039110238	ag_LACat|PROMOTER
L3MBTL4	chr18:6415089-6415136	0,020584455	0,017677814	0,032090299	ag_LACat|PROMOTER
LIMD1	chr3:45634872-45634918	0,030108545	0,023167916	0,033180181	ag_LACat|PROMOTER
LOC100128811	chr10:25468577-25468636	0,04070136	0,024767525	0,033773898	ag_LACat|PROMOTER
LOC100131176	chr7:151096617-151096666	0,039901175	0,028852278	0,036224226	ag_LACat|PROMOTER
LOC100506421	chr2:105468819-105468863	0,048735514	0,024411593	0,033938068	ag_LACat|DIVERGENT_PROMOTER
LOC154860	chr7:121950431-121950483	0,029204305	0,02959259	0,036669079	ag_LACat|DOWNSTREAM
LOC338758	chr12:90096945-90097004	0,0821808	0,014966062	0,033851807	ag_LACat|PROMOTER
LOC646999	chr7:39649165-39649224	0,04968015	0,026702194	0,034749431	ag_LACat|INSIDE
LOC727896	chr18:2949115-2949174	0,023896912	0,011390195	0,042713231	ag_LACat|PROMOTER
LOC730668	chr22:46403119-46403163	0,034700885	0,013755399	0,03469282	ag_LACat|INSIDE

LPAR4	chrX:78007695-78007754	0,022845676	0,049797468	0,049797468	ag_LACat|INSIDE
LPIN1	chr2:11883778-11883829	0,034518003	0,009775233	0,052564932	ag_LACat|PROMOTER
LRRCC1	chr8:86020423-86020482	0,025114134	0,01619735	0,032739324	ag_LACat|INSIDE
LTBP4	chr19:41119512-41119556	0,035482626	0,020459376	0,031862963	ag_LACat|INSIDE
LUC7L2	chr7:139044458-139044502	0,023927497	0,020239752	0,031869223	ag_LACat|PROMOTER
LYSMD3	chr5:89827044-89827103	0,058726724	0,00979264	0,051683378	ag_LACat|PROMOTER
MADD	chr11:47290979-47291030	0,034065735	0,04033892	0,044388387	ag_LACat|PROMOTER
MARCH7	chr2:160569173-160569217	0,042329416	0,034346893	0,039792132	ag_LACat|INSIDE
MBLAC2	chr5:89769902-89769946	0,034345552	0,015436436	0,033328669	ag_LACat|INSIDE
MBTPS2	chrX:21858526-21858585	0,01929005	0,007825501	0,076905786	ag_LACat|INSIDE
MCM3AP	chr21:47704436-47704495	0,031098194	0,006784979	0,241714877	ag_LACat|INSIDE
MESDC2	chr15:81281945-81281993	0,040119883	0,035246707	0,040505288	ag_LACat|INSIDE
METAP1	chr4:99916136-99916195	0,045189515	0,017632104	0,03242032	ag_LACat|PROMOTER
MGST3	chr1:165599935-165599994	0,05765355	0,007976498	0,075776726	ag_LACat|PROMOTER
MIER1	chr1:67396424-67396470	0,022887958	0,016039865	0,032887493	ag_LACat|INSIDE
MINPP1	chr10:89266376-89266435	0,027702656	0,012959867	0,035859826	ag_LACat|PROMOTER
MIR106A	chrX:133309148-133309207	0,028913796	0,016783182	0,032761691	ag_LACat|PROMOTER
MIR1207	chr8:129060662-129060713	0,023939736	0,00714667	0,135786722	ag_LACat|PROMOTER
MIR129-2	chr11:43600437-43600495	0,04445387	0,016861001	0,032468819	ag_LACat|PROMOTER
MIR223	chrX:65235800-65235857	0,025752032	0,009445804	0,052785375	ag_LACat|PROMOTER
MIR2277	chr5:92956523-92956582	0,035777424	0,018787876	0,031683696	ag_LACat|PROMOTER
MIR30C2	chr6:72085603-72085662	0,040924076	0,048959374	0,049305377	ag_LACat|DOWNSTREAM
MIR586	chr6:45168566-45168625	0,027512852	0,010023427	0,050117135	ag_LACat|PROMOTER
MIR943	chr4:1989890-1989937	0,021570824	0,017992781	0,032251211	ag_LACat|PROMOTER
MST4	chrX:131159458-131159517	0,08161232	0,006590629	0,313054854	ag_LACat|INSIDE
MTIF2	chr2:55496160-55496212	0,02983981	0,011252334	0,043930345	ag_LACat|INSIDE
MTIF3	chr13:28017770-28017829	0,037773903	0,006841625	0,216651458	ag_LACat|INSIDE
MYL12A	chr18:3248197-3248252	0,03165288	0,023038553	0,033499937	ag_LACat|INSIDE
NEDD4L	chr18:55854356-55854400	0,041808594	0,012369	0,038317011	ag_LACat|PROMOTER
NEK4	chr3:52745258-52745317	0,031412117	0,008107211	0,072204848	ag_LACat|INSIDE
NEK9	chr14:75595822-75595881	0,03645981	0,018039383	0,031933069	ag_LACat|PROMOTER
NFATC1	chr18:77154879-77154938	0,03961483	0,009222549	0,054758885	ag_LACat|PROMOTER
NLGN4Y	chrY:16732542-16732601	0,034489963	0,02496937	0,0337264	ag_LACat|PROMOTER
NOM1	chr7:156741364-156741408	0,017013544	0,018643603	0,031627541	ag_LACat|PROMOTER
NOP16	chr5:175812709-175812768	0,03543962	0,012654463	0,037180639	ag_LACat|INSIDE
NOXA1	chr9:140324944-140325003	0,025392726	0,03233253	0,038235565	ag_LACat|INSIDE
NOXO1	chr16:2030533-2030579	0,017932685	0,019323403	0,031833352	ag_LACat|INSIDE
NPFFR2	chr4:72889781-72889840	0,08631049	0,013894423	0,03413716	ag_LACat|PROMOTER
NPY5R	chr4:164260776-164260835	0,041848574	0,007786784	0,082193834	ag_LACat|DIVERGENT_PROMOTER
NTPCR	chr1:233087161-233087208	0,045156315	0,017654546	0,032253498	ag_LACat|INSIDE
NUB1	chr7:151038525-151038574	0,030700073	0,021193165	0,032299743	ag_LACat|PROMOTER
NUDT5	chr10:12238110-12238157	0,028561983	0,014349561	0,033798553	ag_LACat|INSIDE
NUP133	chr1:229642967-229643026	0,043267198	0,01966562	0,031844896	ag_LACat|INSIDE
NXT2	chrX:108780691-108780745	0,09473913	0,014104794	0,034066663	ag_LACat|INSIDE
OBP2B	chr9:136082880-136082924	0,041026704	0,03117578	0,037489862	ag_LACat|INSIDE
OFD1	chrX:13752814-13752861	0,031922974	0,032200318	0,038397869	ag_LACat|INSIDE
OMG	chr17:29621640-29621699	0,03259073	0,009261126	0,053865733	ag_LACat|INSIDE
OPTN	chr10:13143484-13143543	0,027907616	0,04790717	0,048589123	ag_LACat|INSIDE
OR6C70	chr12:55862710-55862769	0,022578586	0,012842982	0,035884803	ag_LACat|DOWNSTREAM
PACSIN2	chr22:43356300-43356356	0,06540585	0,010077997	0,049521192	ag_LACat|PROMOTER
PAGE5	chrX:55248922-55248981	0,040450502	0,04382063	0,045579852	ag_LACat|INSIDE
PARP9	chr3:122283333-122283377	0,026593165	0,013417284	0,035406722	ag_LACat|PROMOTER
PCCA	chr13:100955957-100956016	0,06803575	0,00865449	0,061663241	ag_LACat|INSIDE
PCDH9	chr13:67804936-67804982	0,031381756	0,015172694	0,033782951	ag_LACat|PROMOTER
PDCD6IP	chr3:33840712-33840771	0,034284014	0,034731284	0,040074558	ag_LACat|INSIDE
PDE8A	chr15:85523432-85523491	0,02320766	0,021156818	0,032417705	ag_LACat|PROMOTER
PELO	chr5:52083507-52083551	0,04092977	0,04175348	0,044735871	ag_LACat|PROMOTER
PFKFB4	chr3:48594888-48594947	0,019063177	0,007366625	0,116638224	ag_LACat|PROMOTER
PHF6	chrX:133510121-133510180	0,0482987	0,018122202	0,031686059	ag_LACat|INSIDE
PHF7	chr3:52445514-52445573	0,039886955	0,009132391	0,056581118	ag_LACat|INSIDE
PIBF1	chr13:73349641-73349700	0,04377579	0,016400252	0,032915999	ag_LACat|PROMOTER
PIGY	chr4:89445231-89445290	0,039382037	0,027819138	0,035394885	ag_LACat|PROMOTER
PITX1	chr5:134368486-134368545	0,04338398	0,01365971	0,035715756	ag_LACat|INSIDE
PPARGC1A	chr4:23890677-23890729	0,048427943	0,007634236	0,098898051	ag_LACat|INSIDE
PPEF1	chrX:18725447-18725506	0,019384474	0,02041437	0,031967557	ag_LACat|INSIDE
PPP1R3D	chr20:58515175-58515229	0,03001767	0,015442342	0,033090733	ag_LACat|INSIDE
PRPF38B	chr1:109236560-109236619	0,03740504	0,016819054	0,03260837	ag_LACat|INSIDE
PSMD14	chr2:162267401-162267460	0,018937683	0,038449675	0,042805302	ag_LACat|INSIDE
PSMG1	chr21:40556111-40556170	0,03497383	0,012481129	0,037443387	ag_LACat|PROMOTER
PTEN	chr10:89627538-89627597	0,044547364	0,03641891	0,041684295	ag_LACat|INSIDE
PURA	chr5:139486807-139486856	0,040785052	0,013739149	0,034961227	ag_LACat|PROMOTER

PWP2	chr21:45548083-45548137	0,0408783	0,022332545	0,033149871	ag_LACat|INSIDE
RAB11FIP3	chr16:476665-476709	0,038414158	0,032163516	0,038515135	ag_LACat|INSIDE
RAB40AL	chrX:102184715-102184774	0,025571555	0,014620078	0,033602599	ag_LACat|PROMOTER
RAB9A	chrX:13705489-13705548	0,016152173	0,008245861	0,069119717	ag_LACat|PROMOTER
RAD51C	chr17:56769799-56769858	0,027398787	0,008548242	0,062467922	ag_LACat|DIVERGENT_PROMOTER
RAI14	chr5:34686255-34686314	0,02395341	0,010208827	0,048491928	ag_LACat|PROMOTER
RALYL	chr8:85090851-85090910	0,0318493	0,045506068	0,046820323	ag_LACat|PROMOTER
RB1CC1	chr8:53629279-53629338	0,046268154	0,018540544	0,031641048	ag_LACat|PROMOTER
RCOR1	chr14:103058047-103058093	0,0468273	0,024504265	0,033737756	ag_LACat|PROMOTER
RGAG4	chrX:71350706-71350750	0,03282676	0,015874252	0,032783781	ag_LACat|INSIDE
RNF111	chr15:59272956-59273015	0,03363861	0,009268687	0,052831516	ag_LACat|PROMOTER
RNF19A	chr8:101303903-101303962	0,044117544	0,020155847	0,032091712	ag_LACat|INSIDE
ROBO1	chr3:79174076-79174135	0,047780123	0,03426384	0,039857936	ag_LACat|INSIDE
RPS26	chr12:56435614-56435658	0,044526387	0,005893247	0,419893849	ag_LACat|PROMOTER
RPS6KA6	chrX:83447520-83447579	0,046700202	0,01752042	0,032636076	ag_LACat|PROMOTER
RRP12	chr10:99159852-99159911	0,047650125	0,040742073	0,04465958	ag_LACat|INSIDE
S100G	chrX:16670824-16670883	0,056616027	0,007736832	0,088199885	ag_LACat|INSIDE
SAMSN1	chr21:15899171-15899230	0,049066536	0,014349387	0,034079794	ag_LACat|INSIDE
SEMA3A	chr7:83823381-83823440	0,044506874	0,011422304	0,04173534	ag_LACat|INSIDE
SESN1	chr6:109332754-109332813	0,034254167	0,014140284	0,033865386	ag_LACat|PROMOTER
SH3BGRL2	chr6:80339726-80339785	0,033312984	0,04380107	0,045726392	ag_LACat|PROMOTER
SH3RF1	chr4:170192750-170192794	0,07150195	0,010534106	0,044809257	ag_LACat|PROMOTER
SHC2	chr19:447875-447926	0,0885641	0,007315885	0,12264866	ag_LACat|INSIDE
SHOC2	chr10:112682569-112682628	0,02460383	0,017528012	0,032438204	ag_LACat|INSIDE
SIK1	chr21:44852209-44852268	0,02857767	0,022650937	0,033275861	ag_LACat|PROMOTER
SLCO6A1	chr5:101834191-101834249	0,023106633	0,015070592	0,033819832	ag_LACat|INSIDE
SLITRK4	chrX:142730395-142730454	0,042357948	0,024340674	0,034005353	ag_LACat|PROMOTER
SMG5	chr1:156252390-156252434	0,044076733	0,025090888	0,033572315	ag_LACat|INSIDE
SNORA35	chrX:113862309-113862368	0,04723487	0,01670653	0,032836973	ag_LACat|PROMOTER
SOX5	chr12:23741007-23741066	0,05528432	0,011502232	0,041495392	ag_LACat|PROMOTER
SPATA3	chr2:231865149-231865193	0,020655721	0,014662073	0,033429526	ag_LACat|INSIDE
SPATA5	chr4:123844280-123844330	0,025902085	0,045357224	0,047006578	ag_LACat|INSIDE
SPRY1	chr4:124315124-124315183	0,02941629	0,017100822	0,032491562	ag_LACat|PROMOTER
SRP9	chr1:225965969-225966013	0,03110662	0,024050467	0,03393259	ag_LACat|INSIDE
SRSF11	chr1:70688623-70688682	0,038108148	0,01709258	0,032693861	ag_LACat|INSIDE
STAP1	chr4:68425702-68425761	0,08421492	0,00759189	0,103032793	ag_LACat|INSIDE
STATH	chr4:70861857-70861916	0,0377802	0,007484551	0,112268259	ag_LACat|INSIDE
STEAP2	chr7:89838591-89838650	0,043628998	0,010333066	0,046744822	ag_LACat|PROMOTER
TACR2	chr10:71168363-71168412	0,04968087	0,009123244	0,057780545	ag_LACat|INSIDE
TAX1BP1	chr7:27779511-27779570	0,044125628	0,026047533	0,034209894	ag_LACat|PROMOTER
TBC1D5	chr3:17748675-17748734	0,04640004	0,027542628	0,03520022	ag_LACat|PROMOTER
TBX19	chr1:168251893-168251952	0,036500294	0,012443317	0,037727078	ag_LACat|INSIDE
TESK2	chr1:45956222-45956275	0,0300734	0,026690595	0,034893668	ag_LACat|INSIDE
TET2	chr4:106066385-106066444	0,04708505	0,048964776	0,049137187	ag_LACat|PROMOTER
TGFBR1	chr9:101866145-101866204	0,024282573	0,025014518	0,033628008	ag_LACat|PROMOTER
TLE3	chr15:70393444-70393498	0,03343164	0,037741147	0,04268344	ag_LACat|PROMOTER
TMED8	chr14:77843056-77843106	0,03699246	0,048138026	0,048650133	ag_LACat|INSIDE
TMEM146	chr19:5720558-5720602	0,044625	0,006985808	0,16591294	ag_LACat|DIVERGENT_PROMOTER
TMEM180	chr10:104220872-104220931	0,044352725	0,02295242	0,033545845	ag_LACat|DIVERGENT_PROMOTER
TMEM79	chr1:156252766-156252819	0,043907158	0,010569732	0,043657589	ag_LACat|PROMOTER
TMLHE	chrX:154845249-154845308	0,0937705	0,011420756	0,042271629	ag_LACat|PROMOTER
TMSL3	chrX:12992544-12992603	0,029340591	0,0284059	0,0358216	ag_LACat|PROMOTER
TNNT1	chr19:55657518-55657564	0,04093247	0,03759596	0,04268864	ag_LACat|INSIDE
TPR	chr1:186343783-186343842	0,034375094	0,008527536	0,065685075	ag_LACat|INSIDE
TRIM41	chr5:180645198-180645257	0,02117839	0,004590897	0,65420288	ag_LACat|PROMOTER
TRPM6	chr9:77501346-77501405	0,028100982	0,007642831	0,094704641	ag_LACat|INSIDE
TRPS1	chr8:116636913-116636972	0,03674045	0,019073341	0,031788902	ag_LACat|INSIDE
TSPAN32	chr11:2327593-2327652	0,042179383	0,043644328	0,045899017	ag_LACat|INSIDE
TWF1	chr12:44200796-44200855	0,0403923	0,024687666	0,03382685	ag_LACat|PROMOTER
UBE3A	chr15:25686148-25686207	0,061822966	0,01194519	0,038686127	ag_LACat|PROMOTER
UBQLN2	chrX:56587976-56588035	0,0335687	0,045398153	0,046878528	ag_LACat|PROMOTER
UBR4	chr1:19413735-19413784	0,028635649	0,011156054	0,044781344	ag_LACat|INSIDE
UGGT2	chr13:96541417-96541476	0,045535468	0,011704802	0,040681324	ag_LACat|INSIDE
UTY	chrY:15590011-15590070	0,048916467	0,016580574	0,032815719	ag_LACat|INSIDE
VPS37A	chr8:17105230-17105289	0,020188335	0,01812027	0,031878253	ag_LACat|INSIDE
WAPAL	chr10:88279001-88279060	0,03965783	0,020157114	0,031915431	ag_LACat|INSIDE
WIPI1	chr17:66452864-66452911	0,032245547	0,037334103	0,042560877	ag_LACat|INSIDE
WWC2	chr4:184021922-184021981	0,028372785	0,029662456	0,036282403	ag_LACat|INSIDE
YBEY	chr21:47706344-47706388	0,020486282	0,003880443	1,105926341	ag_LACat|INSIDE
ZC3HAV1L	chr7:138720720-138720764	0,038375795	0,012762369	0,036740153	ag_LACat|INSIDE
ZCWPW1	chr7:100026080-100026135	0,041587733	0,038829308	0,043059738	ag_LACat|INSIDE

ZNF277	chr7:111852010-111852069	0,03063503	0,029001636	0,036252045	ag_LACat|INSIDE
ZNF512B	chr20:62600324-62600368	0,045512922	0,037946343	0,04274588	ag_LACat|INSIDE
ZNF746	chr7:149194163-149194207	0,033940524	0,0340478	0,039768947	ag_LACat|INSIDE

Supplementary Table S5

Primary Annotation	Name	P-value	P[Xbar]	FDR	Primary Annotation Type
ABCA3	chr16:2374613-2374659	0,04803557	0,04455205	0,046802154	ag_LACat|INSIDE
ADAMTS8	chr11:130297928-130297972	0,031003352	0,031365566	0,041820755	ag_LACat|INSIDE
ADAT1	chr16:75657031-75657081	0,08851115	0,0103496	0,02690896	ag_LACat|INSIDE
AK094279:-3506	chr10:92923399-92923455	0,039088223	0,03932605	0,045443436	ag_LACat|PROMOTER
ARHGAP22	chr10:49731526-49731570	0,09276065	0,006277597	0,065287005	ag_LACat|INSIDE
ARHGEF3	chr3:56809598-56809653	0,04023816	0,045241274	0,047050925	ag_LACat|INSIDE
ATP6V1B2	chr8:20055053-20055101	0,045496754	0,021678872	0,034686195	ag_LACat|INSIDE
B3GNT2	chr2:62424509-62424568	0,035546344	0,011807266	0,026694688	ag_LACat|INSIDE
BCL2	chr18:60986874-60986930	0,024018427	0,008907365	0,031943654	ag_LACat|PROMOTER
BNC1	chr15:83953895-83953939	0,060343813	0,005696027	0,118477362	ag_LACat|PROMOTER
C11orf73	chr11:86013236-86013281	0,047857974	0,0410926	0,045464153	ag_LACat|PROMOTER
C16orf91	chr16:1479366-1479419	0,04650722	0,03371502	0,042760513	ag_LACat|PROMOTER
C17orf104	chr17:42734022-42734066	0,09013072	0,00830579	0,035991757	ag_LACat|INSIDE
C18orf18	chr18:5237613-5237672	0,039915692	0,007250368	0,047127392	ag_LACat|INSIDE
C20orf7	chr20:13765612-13765662	0,03344415	0,019253675	0,032825938	ag_LACat|DIVERGENT_PROMOTER
CACNA1D	chr3:53530139-53530194	0,041120674	0,024248652	0,036026569	ag_LACat|INSIDE
CCDC12	chr3:47018483-47018533	0,098604895	0,010266527	0,027377405	ag_LACat|DIVERGENT_PROMOTER
CD59	chr11:33746008-33746065	0,044582535	0,03510319	0,04398472	ag_LACat|PROMOTER
CDC42EP5	chr19:54976273-54976317	0,04371079	0,030549303	0,041804309	ag_LACat|INSIDE
CDH8	chr16:62069907-62069959	0,04203058	0,007239659	0,050194971	ag_LACat|INSIDE
CNPY1	chr7:155326394-155326453	0,029927526	0,006046685	0,089836469	ag_LACat|INSIDE
COL14A1	chr8:121137499-121137543	0,0404396	0,02303761	0,034723354	ag_LACat|INSIDE
CXCR3	chrX:70842806-70842850	0,032510165	0,038956314	0,04603928	ag_LACat|PROMOTER
CXorf36	chrX:45059737-45059793	0,030698046	0,033274554	0,042722884	ag_LACat|INSIDE
DOPEY2	chr21:37529681-37529725	0,044461947	0,040637616	0,045938175	ag_LACat|PROMOTER
EIF4EBP1	chr8:37889419-37889473	0,049636595	0,030481648	0,042267885	ag_LACat|INSIDE
ENTPD5	chr14:74485274-74485324	0,0385141	0,039160464	0,045760542	ag_LACat|INSIDE
EPB41L4A	chr5:111755094-111755138	0,09437824	0,009846219	0,028444633	ag_LACat|DIVERGENT_PROMOTER
ESRP2	chr16:68271322-68271366	0,056313027	0,007425144	0,040642893	ag_LACat|DIVERGENT_PROMOTER
FAM123A	chr13:25744623-25744682	0,04515108	0,022997772	0,035173063	ag_LACat|INSIDE
FAM53B	chr10:126432398-126432457	0,039710507	0,0380302	0,045461389	ag_LACat|INSIDE
FAM84B	chr8:127569345-127569389	0,041859593	0,010622143	0,026943972	ag_LACat|INSIDE
FLJ45983	chr10:8091779-8091824	0,08078633	0,007278086	0,04452476	ag_LACat|DOWNSTREAM
GATA4	chr8:11554911-11554968	0,030600473	0,04907969	0,04907969	ag_LACat|PROMOTER
GCAT	chr22:38203618-38203662	0,03988873	0,009902396	0,027833762	ag_LACat|PROMOTER
GFOD1	chr6:13488126-13488173	0,031897347	0,009798369	0,029115154	ag_LACat|PROMOTER
GRPEL1	chr4:7069291-7069335	0,029339923	0,007374064	0,042605701	ag_LACat|INSIDE
GSN	chr9:124061742-124061801	0,024333937	0,008330782	0,034656053	ag_LACat|PROMOTER
GTDC1	chr2:145089435-145089479	0,0660386	0,006974782	0,055798258	ag_LACat|INSIDE
GUCA2A	chr1:42629113-42629158	0,04167965	0,024865657	0,03591706	ag_LACat|INSIDE
H2AFJ	chr12:14932078-14932137	0,034531265	0,035963677	0,044002617	ag_LACat|DOWNSTREAM
HAAO	chr2:43020171-43020215	0,036847025	0,008543624	0,031733461	ag_LACat|PROMOTER
HHAT	chr1:210502816-210502870	0,048918534	0,00767106	0,039889512	ag_LACat|INSIDE
HSPA4L	chr4:128704022-128704066	0,042910542	0,021245915	0,034524612	ag_LACat|INSIDE
IRF2BP2	chr1:234746108-234746152	0,033752356	0,040158525	0,045895457	ag_LACat|PROMOTER
KCNS3	chr2:18060348-18060394	0,03141217	0,006561023	0,06203149	ag_LACat|INSIDE
LOC100132831	chrX:40691467-40691515	0,045099042	0,008353467	0,033413868	ag_LACat|INSIDE
LOC441204	chr7:26438015-26438069	0,044785965	0,027083473	0,038584674	ag_LACat|PROMOTER
LRP1B	chr2:142888254-142888299	0,04392528	0,009556762	0,032061395	ag_LACat|INSIDE
MIR124-3	chr20:61807101-61807145	0,09744925	0,010735963	0,026584289	ag_LACat|PROMOTER
MIR505	chrX:139006244-139006303	0,09687852	0,007738547	0,038324235	ag_LACat|DOWNSTREAM
MIR514-2	chrX:146369750-146369803	0,041718166	0,016685309	0,029918485	ag_LACat|PROMOTER
MSMP	chr9:35756835-35756884	0,033933375	0,009604462	0,030268608	ag_LACat|PROMOTER
NGLY1	chr3:25831465-25831509	0,03799917	0,0187712	0,032536747	ag_LACat|PROMOTER
NINJ1	chr9:95896459-95896503	0,03615347	0,045297984	0,046643469	ag_LACat|INSIDE
NKX2-2	chr20:21494309-21494361	0,0389405	0,008284113	0,037458598	ag_LACat|INSIDE
NLRP12	chr19:54313894-54313946	0,05854107	0,007178996	0,053329683	ag_LACat|PROMOTER
NOL8	chr9:95063683-95063742	0,03981939	0,037158445	0,044935794	ag_LACat|INSIDE
NPR3	chr5:32708234-32708293	0,029073762	0,009478378	0,032858377	ag_LACat|PROMOTER
OGFOD1	chr16:56485368-56485414	0,04980368	0,015061465	0,028479861	ag_LACat|DIVERGENT_PROMOTER
OSBPL5	chr11:3157196-3157240	0,032765612	0,033107284	0,043039469	ag_LACat|INSIDE
OXSM	chr3:25831668-25831717	0,029146226	0,040792126	0,045617001	ag_LACat|INSIDE
PCDH11X	chrY:4868597-4868656	0,03969371	0,029888585	0,042005579	ag_LACat|INSIDE
PCDH11Y	chrY:4868786-4868831	0,026135884	0,006206465	0,071719145	ag_LACat|INSIDE
PCDH17	chr13:58206273-58206332	0,032372884	0,021747086	0,034268136	ag_LACat|INSIDE
PCDH19	chrX:99664127-99664171	0,088905185	0,008493331	0,032715053	ag_LACat|INSIDE
PDZRN3	chr3:73674097-73674141	0,06917291	0,006015661	0,104271457	ag_LACat|PROMOTER
PHACTR4	chr1:28696708-28696757	0,03895352	0,008155284	0,038552249	ag_LACat|INSIDE
PHF12	chr17:27277255-27277299	0,030011617	0,011837066	0,026192657	ag_LACat|INSIDE

PIM3	chr22:50352318-50352366	0,047818005	0,010796173	0,025518227	ag_LACat|PROMOTER
PNMAL2	chr19:46997566-46997610	0,02772622	0,02468036	0,036151513	ag_LACat|INSIDE
PPM1L	chr3:160472735-160472780	0,033354186	0,004123451	0,214419436	ag_LACat|PROMOTER
PRAME	chr22:22899992-22900036	0,04281526	0,014119091	0,027705386	ag_LACat|INSIDE
PRELID2	chr5:145203773-145203832	0,03641192	0,04871617	0,049189143	ag_LACat|INSIDE
PRICKLE3	chrX:49040936-49040980	0,034829427	0,009603377	0,031210974	ag_LACat|INSIDE
PRR7	chr5:176881527-176881571	0,03598398	0,012821981	0,027214	ag_LACat|INSIDE
RAB23	chr6:57086950-57087009	0,03446622	0,013840557	0,028788359	ag_LACat|PROMOTER
RAB33A	chrX:129304032-129304091	0,04046954	0,042121556	0,046112019	ag_LACat|DIVERGENT_PROMOTER
RFX7	chr15:56537022-56537080	0,025852697	0,004846196	0,168001461	ag_LACat|PROMOTER
RUNDC3B	chr7:87254994-87255053	0,032864895	0,005130726	0,133398876	ag_LACat|PROMOTER
S100A6	chr1:153508705-153508749	0,04529887	0,018573247	0,032739283	ag_LACat|PROMOTER
SAFB	chr19:5623028-5623072	0,041069675	0,006168202	0,080186626	ag_LACat|INSIDE
SHROOM2	chrX:9753501-9753546	0,05495067	0,004023687	0,418463448	ag_LACat|PROMOTER
SLC26A6	chr3:48671666-48671710	0,046465673	0,03573139	0,044238864	ag_LACat|PROMOTER
SLC2A2	chr3:170746108-170746160	0,033896733	0,010054018	0,02751626	ag_LACat|PROMOTER
SLC38A1	chr12:46661084-46661129	0,03165353	0,047828943	0,048766765	ag_LACat|INSIDE
SLCO4A1	chr20:61267610-61267654	0,03450949	0,010795541	0,026110146	ag_LACat|PROMOTER
SPTB	chr14:65236182-65236228	0,028868232	0,020748697	0,034251817	ag_LACat|INSIDE
SV2C	chr5:75380461-75380505	0,04377516	0,006574479	0,056978818	ag_LACat|INSIDE
TAF10	chr11:6633552-6633596	0,0451474	0,016474515	0,030058764	ag_LACat|PROMOTER
TCF7L2	chr10:114710993-114711039	0,026377382	0,011435253	0,02642814	ag_LACat|INSIDE
THAP2	chr12:72070884-72070943	0,044565074	0,04394927	0,046640042	ag_LACat|INSIDE
TKTL1	chrX:153523954-153524010	0,04798094	0,009695594	0,029657111	ag_LACat|DIVERGENT_PROMOTER
TMPRSS2	chr21:42879497-42879543	0,04779052	0,021887098	0,033974003	ag_LACat|INSIDE
TMSB15B	chrX:103217263-103217307	0,037398778	0,03067636	0,041433006	ag_LACat|INSIDE
TNNT3	chr11:1959318-1959376	0,04347247	0,014082586	0,028165172	ag_LACat|INSIDE
TNS3	chr7:47626581-47626640	0,0486528	0,043460194	0,047081877	ag_LACat|PROMOTER
TOP3B	chr22:22313127-22313182	0,025463402	0,014050489	0,028651978	ag_LACat|INSIDE
TTLL12	chr22:43581819-43581863	0,028496293	0,016387604	0,030434122	ag_LACat|INSIDE
TUBB3	chr16:89988649-89988696	0,036438175	0,020279106	0,034016565	ag_LACat|PROMOTER
UGT3A2	chr5:36066561-36066615	0,042989463	0,032264724	0,04247508	ag_LACat|INSIDE
UTP3	chr4:71554231-71554275	0,04444203	0,014716647	0,028343172	ag_LACat|INSIDE
ZNF35	chr3:44690131-44690175	0,039751988	0,04381779	0,046979899	ag_LACat|PROMOTER
ZNF521	chr18:22931432-22931491	0,04149751	0,012027297	0,026059144	ag_LACat|INSIDE

Table S6. Lack of SND1 binding to representative target genes in siRNA SND1-treated HepG2 cells

Name/Gene ID	Description	Fold enrichment
ACAA2	acetyl-CoA acyltransferase 2	undetermined
ADAT1	adenosine deaminase, tRNA-specific 1	0.38
ATF1	activating transcription factor 1	0.92
AZI2	5-azacytidine induced 2	undetermined
BRCA2	breast cancer 2, early onset	0.17
CALM1	calmodulin 1 (phosphorylase kinase, delta)	undetermined
CCNI	cyclin I	0.35
CCND1	cyclin D1	0.13
CD36	CD36 molecule (thrombospondin receptor)	0.14
CDKN1B	cyclin-dependent kinase inhibitor 1B (p27, Kip1)	0.27
CHDH	choline dehydrogenase	0.25
CHPT1	choline phosphotransferase 1	0.19
CREM	cAMP responsive element modulator	0.28
EIF4B	eukaryotic translation initiation factor 4B	0.91
FADS2	fatty acid desaturase 2	0.25
FLNA	filamin A, alpha	0.44
GK	glycerol kinase	1.22
HCFC1	host cell factor C1 (VP16-accessory protein)	undetermined
HOXA3	homeobox A3	0.46
HOXB9	homeobox B9	0.20
HOXC9	homeobox C9	0.27
HSD17B2	hydroxysteroid (17-beta) dehydrogenase 2	0.28
IRAK4	interleukin-1 receptor-associated kinase 4	0.22
LPGAT1	lysophosphatidylglycerol acyltransferase 1	0.29
LPIN1	lipin 1	0.35
LRPAP1	low density lipoprotein receptor-related protein associated protein 1	0.10

MADD	MAP-kinase activating death domain	0.15
MBTPS	membrane-bound transcription factor peptidase, site 2	1.37
MGLL	monoglyceride lipase	0.16
NFKB2	nuclear factor of kappa light polypeptide gene enhancer in B-cells 2 (p49/p100)	0.45
PPA2	pyrophosphatase (inorganic) 2	undetermined
PPARGC1A	peroxisome proliferator-activated receptor gamma, coactivator 1 alpha	0.23
PTDSS1	phosphatidylserine synthase 1	0.72
PTEN	phosphatase and tensin homolog	undetermined
RXRA	retinoid X receptor, alpha	undetermined
SCAP	SREBF chaperone	0.01
SETD1A	SET domain containing 1A	0.44
TAF10	TAF10 RNA polymerase II, TATA box binding protein (TBP)-associated factor, 30kDa	0.37
TDRD3	tudor domain containing 3	undetermined
TRAF7	TNF receptor-associated factor 7	0.19
*WNT7B**	wingless-type MMTV integration site family, member 7B	14.16

* Indicates a significant enrichment with a specific/unspecific amplification ratio>2. Amplification was undetermined in 8 genes.
